# The self-organizing fractal theory as a universal discovery method: the phenomenon of life

**DOI:** 10.1186/1742-4682-8-4

**Published:** 2011-03-29

**Authors:** Alexei Kurakin

**Affiliations:** 1 Department of Pathology, Beth Israel Deaconess Medical Center and Harvard Medical School, Boston, MA 02215, USA

## Abstract

A universal discovery method potentially applicable to all disciplines studying organizational phenomena has been developed. This method takes advantage of a new form of global symmetry, namely, scale-invariance of self-organizational dynamics of energy/matter at all levels of organizational hierarchy, from elementary particles through cells and organisms to the Universe as a whole. The method is based on an alternative conceptualization of physical reality postulating that the energy/matter comprising the Universe is far from equilibrium, that it exists as a flow, and that it develops via self-organization in accordance with the empirical laws of nonequilibrium thermodynamics. It is postulated that the energy/matter flowing through and comprising the Universe evolves as a multiscale, self-similar structure-process, i.e., as a self-organizing fractal. This means that certain organizational structures and processes are scale-invariant and are reproduced at all levels of the organizational hierarchy. Being a form of symmetry, scale-invariance naturally lends itself to a new discovery method that allows for the deduction of missing information by comparing scale-invariant organizational patterns across different levels of the organizational hierarchy.

An application of the new discovery method to life sciences reveals that moving electrons represent a keystone physical force (flux) that powers, animates, informs, and binds all living structures-processes into a planetary-wide, multiscale system of electron flow/circulation, and that all living organisms and their larger-scale organizations emerge to function as electron transport networks that are supported by and, at the same time, support the flow of electrons down the Earth's redox gradient maintained along the core-mantle-crust-ocean-atmosphere axis of the planet. The presented findings lead to a radically new perspective on the nature and origin of life, suggesting that living matter is an organizational state/phase of nonliving matter and a natural consequence of the evolution and self-organization of nonliving matter.

The presented paradigm opens doors for explosive advances in many disciplines, by uniting them within a single conceptual framework and providing a discovery method that allows for the systematic generation of knowledge through comparison and complementation of empirical data across different sciences and disciplines.

## Introduction

It is a self-evident fact that life, as we know it, has a natural tendency to expand in space and time and to evolve from simplicity to complexity. Periodic but transient setbacks in the form of mass extinctions notwithstanding, living matter on our planet has been continuously expanding in terms of its size, diversity, complexity, order, and influence on nonliving matter. In other words, living matter as a whole appears to evolve spontaneously from states of relative simplicity and disorder (i.e., high entropy states) to states of relative complexity and order (i.e., low entropy states). Moreover, when considered over macroevolutionary timescales, the expansion and ordering of living matter appears to proceed at an accelerating pace [1,2]. Yet this empirical trend stands in stark contrast with one of the fundamental laws of physics, the second law of thermodynamics, which states that energy/matter can spontaneously evolve only from states of lower entropy (order) to states of higher entropy (disorder), i.e., in the opposite direction. The apparent conflict between theory and empirical reality is normally dismissed by pointing out that the second law does not really contradict biological evolution because local decreases in entropy (i.e., ordering) are possible as long as there are compensating increases in entropy (i.e., disordering) somewhere else, so that net entropy always increases. Albeit, how exactly the apparent decrease of entropy on the planet Earth is compensated by an increase in entropy somewhere else is less clear. Since "somewhere else" can potentially include the whole Universe, the Universe as a whole is believed to undergo natural disorganization on the way to its final destination, i.e., to a state of maximum entropy, where all changes will cease, and disorder and simplicity will prevail forever. A gloomy future indeed, so that one may ask oneself why to bother, to excel, and to create, and why not simply enjoy by destroying, since this is the natural and inevitable order of things anyway? Yet, most of us do bother, excel, and create, for this makes our lives meaningful. A logical conclusion is that either most people are mad, being in denial of reality and behaving irrationally, or that the accepted theory presents us with a false image of reality that conflicts sharply with our deep-seated beliefs, intuition, and common sense.

Revising the basic concepts, assumptions, and postulates placed as keystones in the foundation of classical physics and the corresponding worldview at the very beginning, this work outlines an alternative interpretation/image of reality that brings scientific theory, experimental reality, and our deep-seated beliefs, intuition, and common sense into harmony. Moreover, the proposed interpretation naturally resolves a large variety of paradoxes and reconciles numerous controversies burdening modern sciences.

Let us begin by noting that the apparent conflict between the second law of thermodynamics and biological evolution exists only if one assumes that the energy/matter comprising the Universe is *near equilibrium *and that it evolves *toward an equilibrium state *via disorganization and disordering, obeying the laws of *equilibrium thermodynamics*. The conflict disappears, however, if we postulate that the energy/matter making up the Universe is *far from equilibrium*, that it exists as an evolving flow, and that the energy/matter flowing through and comprising the Universe evolves from simplicity and disorder to complexity and order via self-organization, in accordance with the empirical laws of *nonequilibrium thermodynamics*.

Studies on self-organization in relatively simple nonequilibrium systems show that creating a gradient (e.g., a temperature, concentration, or chemical gradient) within a molecular system of interacting components normally causes a flux of energy/matter in the system and, as a consequence, the emergence of a countervailing gradient, which, in turn, may cause the emergence of another flux and another gradient, and so forth. The resulting complex system of conjugated fluxes and coupled gradients manifests as a spatiotemporal macroscopic order spontaneously emerging in an initially featureless, disordered system, provided the system is driven far enough away from equilibrium [3-5].

**Figure 1:**
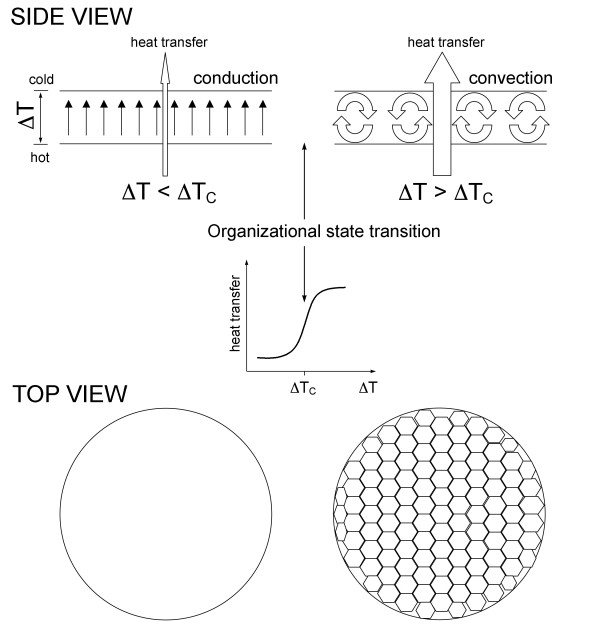
**The Benard instability**. Establishing an increasing vertical temperature gradient (ΔT) across a thin layer of liquid leads to heat transfer through the layer by conduction (organizational state #1). Exceeding a certain critical value of temperature gradient (ΔT_C_) leads to an organizational state transition within the liquid layer. As a result of the transition, conduction is replaced by convection (organizational state #2) and the rate of heat transfer through the layer increases in a stepwise manner. Organizational state #2 (convection) is a more ordered state (higher negative entropy) than organizational state #1 (conduction). The more ordered state requires and, at the same time, supports a higher rate of energy/matter flow through the system. For this reason, the transitions between organizational states in nonequilibrium systems tend to be all-or-none phenomena. As a consequence, nonequilibrium systems are inherently quantal, absorbing and releasing energy/matter as packets. Organizational state #2 (convection) will relax into organizational state #1 (conduction) upon decreasing the temperature gradient (not shown). The Benard instability is an example of a nonequilibrium system illustrating a number of universal self-organizational processes shared by all nonequilibrium systems, including living cells and organisms (see discussion in the text). Reproduced from [8].

One of the classical examples of nonequilibrium systems is the Belousov-Zhabotinsky reaction, in which malonic acid is oxidized by potassium bromate in dilute sulfuric acid in the presence of a catalyst, such as cerium or manganese. By varying experimental conditions, one can generate diverse ordered spatiotemporal patterns of reactants in solution, such as chemical oscillations, stable spatial structures, and concentration waves [4,5]. Another popular example is the Benard instability shown in Figure 1. In this system, a vertical temperature gradient, which is created within a thin horizontal layer of liquid by heating its lower surface, drives an upward heat flux through the liquid layer. When the temperature gradient is relatively weak, heat propagates from the bottom to the top by conduction. Molecules move in a seemingly uncorrelated fashion, and no macro-order is discernable. However, once the imposed temperature gradient reaches a certain threshold value, an abrupt organizational transition takes place within the liquid layer, leading to the emergence of a metastable macroorganization of molecular motion. Molecules start moving coherently, forming hexagonal convection cells of a characteristic size. As a result of the organizational transition, conduction is replaced by convection, and the rate of energy/matter transfer through the layer increases in a stepwise manner.

Several empirical generalizations discovered in studies of far-from-equilibrium systems are especially relevant for the discussion that follows.

First, a sufficiently intense flow of energy/matter through an open physicochemical system of interacting components *naturally and spontaneously *leads to the emergence of interdependent fluxes and gradients within the system, with concomitant dynamic compartmentalization of the components of the system in space and time.

Second, the emergence of macroscopic order is a highly nonlinear, cooperative process. When a critical threshold value of flow rate is exceeded, the system spontaneously self-organizes into interdependent and interconnected macrostructures-processes, in a phase transition-like manner. The macrostructures-processes emerging in far-from-equilibrium conditions are of a steady-state nature. That is, what is actually preserved and evolves over relevant timescales is an *organization *of relationships between interacting components (an organizational form) but not physical components comprising a given macrostructure. Members come and go, but the organization persists. Normally, the same set of interacting microcomponents can generate multiple alternative organizational configurations differing in the organization of energy/matter exchanges transiently maintained among the interacting components that make up and flow through a given configuration. As a consequence, macrostructures-processes emerging in far-from-equilibrium systems are dynamic in two different senses, for they display both configurational dynamics and flow dynamics. Among other things, this means that, within a nonequilibrium system of energy/matter flow/circulation, everything is connected to everything else through shared microcomponents flowing through and mediating the emergence, evolution, and transformation of diverse organizational forms comprising the system.

Third, the degree of complexity and order within a self-organizing nonequilibrium system and the rate of energy/matter passing through the system correlate in a mutually defining manner. A relatively higher degree of complexity and order requires and, at the same time, supports a relatively higher rate of energy/matter flow. Increasing the rate of energy/matter flow normally leads to a stepwise increase in relative complexity and order within an evolving nonequilibrium system. Conversely, decreasing the rate of energy/matter flow results in organizational relaxation via a stepwise decrease in relative complexity and order. The mutually defining relationship between the order within a nonequilibrium system and the rate of energy/matter flow through the system accounts for the inherently quantal nature of nonequilibrium systems, which absorb and release energy/matter in packets (i.e., as quanta).

As the first postulate, let us assume that, at the fundamental level, the energy/matter comprising the Universe is far from equilibrium, that it exists as an evolving flow, and that the energy/matter comprising and flowing through the Universe spontaneously self-organizes on multiple spatiotemporal scales into metastable, interconverting flow/circulation patterns (organizational forms). These forms are manifested at the corresponding levels of the organizational hierarchy as elementary particles, atoms, molecules, cells, organisms, ecosystems (including human organizations and economies), planetary and stellar systems, galaxies, and so forth. All of the scale-specific manifestations/forms of flowing energy/matter are thus interconnected and co-evolve as a nested set of self-organizing and interdependent structures-processes.

As the second postulate, let us assume that, notwithstanding periodic but transient setbacks in the form of organizational relaxations and restructuring (which occur on multiple scales of space and time), the energy/matter comprising the Universe evolves from simplicity and disorder to complexity and order via self-organization, in accordance with the empirical laws of nonequilibrium thermodynamics (NET).

The third postulate pertains to the spatiotemporal organization/structure of evolving energy/matter. Recently, it was proposed that living matter as a whole represents a multiscale structure-process of energy/matter flow/circulation, which obeys the empirical laws of nonequilibrium thermodynamics and which evolves as a self-similar structure (fractal) due to the pressures of economic competition and evolutionary selection [6-9]. According to the self-organizing fractal theory (SOFT) of living matter, certain organizational structures and processes are scale-invariant and occur over and over again on all scales of the biological organizational hierarchy, at the molecular, cellular, organismal, populational, and higher-order levels of biological organization. The SOFT implies the existence of universal principles governing self-organizational dynamics in a scale-invariant manner. As the third postulate, let us assume that the energy/matter flowing through and comprising the Universe spontaneously self-organizes into self-similar (fractal) structures-processes on *all scales *of the organizational hierarchy.

The third postulate is of special importance because, by positing a new form of global symmetry, it provides both a hypothesis and a means to verify this hypothesis. Indeed, the scale-invariance of organizational dynamics allows for the deduction of missing information by comparing scale-invariant organizational patterns across different levels of the organizational hierarchy, and the inferences made from symmetry considerations can be either tested through experimentation or immediately verified with existing experimental data. Because the SOFT-NET theory tacitly implies that most of the accumulated empirical data is correct but misinterpreted, great discoveries can be made simply by reconceptualizing and restructuring existing knowledge.

As a matter of fact, we see not with eyes but with concepts, and, in the same way as the mind of a child matures by acquiring new concepts that allow him/her to see new meanings while looking at the same reality, our collective understanding of the world and our place in it develops through the continuous acquisition of new concepts that reveal an increasingly adequate image of reality.

Since the SOFT-NET interpretation is about an energy/matter flow, and the main focus of this article is the phenomenon of life, let us begin with a review of what is currently known about the propagation of elementary forms of energy/matter such as electrons and protons within living matter.

### Propagation of electrons and protons in biological macromolecules

Water is a relatively unstructured, homogeneous, and isotropic medium. Within such a medium, electron transfer (ET) occurs over short molecular distances and has no preferred pathways or directions. The distances and frequencies of ET in bulk water have Gaussian distribution and decay rapidly for larger values. In contrast, biological macromolecules, such as proteins, nucleic acids, and lipids, together with the ordered molecules of interfacial water, represent dense, structured, highly inhomogeneous, and anisotropic media that have evolved to mediate the efficient transport of electrons over long molecular distances and along preferred pathways and directions.

In the 1960s, it was discovered that electrons move through proteins by means of quantum mechanical tunneling between redox groups [10,11]. The rate of electron tunneling is defined by the difference in redox potentials between donor and acceptor (the driving force), the reorganization energy associated with nuclear rearrangements accompanying charge transfer, and the electronic coupling between donor and acceptor [12,13]. In the late 1980s, Onuchic and Beratan proposed that ET rates in a protein matrix are defined by the strengths of the pathways coupling donors and acceptors, rather than decaying exponentially with the linear distance separating redox centers. Because ET takes place preferentially through covalent and hydrogen bonds, and less frequently, through van der Waals contacts and space, due to the energy penalties associated with the corresponding transfers, the balance between through-bond and through-space contacts between donor and acceptor was proposed to set the coupling strength [14,15]. Such an interpretation implies that electron transfer between redox centers in proteins can occur along multiple, competing tunneling pathways, with the probability of ET along a given pathway being defined by protein structure and dynamics. Since then, the tunneling-pathway model has proven to be one of the most useful methods for estimating distant electronic couplings and ET rates. According to current views, protein structure and dynamics are the key determinants of biological ET rates, as they establish the driving force, the reorganization energy, and the electronic coupling [13].

The propagation of electrons over distances longer than approximately 20 angstroms is believed to take place by multistep tunneling, which involves electron transport through a chain of coupled intermediate redox centers connecting the donor and acceptor. Multistep tunneling is a viable method for delivering charges over long molecular distances, especially if it involves endergonic steps [13]. However, electron transfer over increasingly longer distances requires increasingly greater precision in positioning and structuring and finer control of reaction driving forces. It is reasonable to expect that the distances and frequencies of ET within proteins do not follow Gaussian distribution but are more accurately described by power-law or log-normal distributions. This may mean that the probability of high-frequency and/or long-distance ET through a protein medium is not prohibitively small but remains significant enough to be functionally meaningful, whatever the size of the protein medium may be.

As a biologically relevant case of intermolecular ET, a redox reaction between two soluble proteins involves the following basic steps: i) formation of an active donor-acceptor complex, ii) electron transfer between the donor and acceptor, and iii) dissociation of the oxidized and reduced products [13]. This implies that efficient, long-distance ET within dynamic multiprotein complexes inside living cells would require the formation of short-lived, weak, but specific protein-protein associations, accompanied by specific yet flexible coupling of ET pathways at protein interaction interfaces. Remarkably, virtually everything we know about the physicochemistry of proteins and protein-protein interactions matches these requirements precisely, including such details as the surprisingly weak affinities of the most specific protein-protein interactions driving the assembly of macromolecular complexes in the cell; the dynamic, adaptive, multiconformational nature of proteins [16,17], which may have evolved to balance stability versus flexibility in electronic couplings; the existence of evolutionary conserved pathways of physically and/or thermodynamically linked amino acids that traverse through proteins, coupling interaction interfaces, and active sites [18-22]; the highly inhomogeneous distribution of interaction energy on protein interaction interfaces ("hot spots") [23]; and the specific spatial organization and chemical composition of protein interaction interfaces [24], including the relative abundance of structured water acting to facilitate intermolecular ET [25,26], among others. Altogether, it appears that the physicochemical properties of proteins have been carefully tailored by evolution to support electron transport through proteins and multiprotein complexes.

In fact, the hypothesis of electron flow through proteins, protein complexes, and the intracellular organization as a whole was suggested as early as 1941, by Albert Szent-Gyorgyi, the discoverer of vitamin C and a Nobel laureate, who also felt that the cell represents and functions as an energy continuum [27]. Although, electron conduction in proteins was rejected at the time by physicists on theoretical grounds (like many other physical phenomena, such as high-temperature superconductivity, for example), the experimental demonstration of electron and proton tunneling in proteins later led to the revival of interest in Szent-Gyorgyi's ideas [10,11,28]. Currently, long-range electron and proton transfer in proteins as well as the intimate relationships among electron transfer, hydrogen transfer, enzymatic catalysis, and protein structure and dynamics are the subject of intense research efforts, which are leading to a drastic revision of the classical models of enzymatic catalysis [13,22,29-32]. Briefly, because most, perhaps all, enzymatic reactions involve the transfer of electrons and/or hydrogen (in the form of an atom, proton, or hydride) as an essential step, it has been proposed that the structures and dynamics of enzymes have been shaped by evolution in such a way as to decrease and narrow fluctuating energy barriers within protein matrices in a specific manner, thus enabling electron and hydrogen transfer along preferred trajectories and directions. Indeed, it is now well established that enzymatic catalysis is tightly coupled to intrinsic protein motions that occur in enzymes on microsecond to millisecond timescales in the absence of any substrate [33-35]. In addition, a rapidly increasing number of enzyme-catalyzed reactions are being recognized to involve the formation of transient radical intermediates along electron-conducting pathways in proteins, with radicals playing the role of "stepping stones" for moving electrons [36-38].

The DNA double helix, with its π-stacked array of heterocyclic aromatic base pairs, is another medium capable of supporting efficient long-range charge transport (CT) in the form of moving electrons and holes. Since the first report more than 15 years ago by Barton and colleagues on rapid electron transfer along the DNA helix over a distance greater than 40 angstroms [39], multiple studies from different research groups have confirmed that long-range DNA-mediated CT is efficient over distances of at least 200 angstroms. Charge transfer in DNA is characterized by a very shallow distance dependence and exquisite sensitivity to stacking perturbations, such as mismatched, bulged, or damaged base pairs (see [40,41] and references therein).

It is worth mentioning the remarkable and revealing parallels in the evolution of views on electron transport in proteins and DNA. At first, proteins and DNA were believed to be insulators, until long-range electron tunneling in both media had been experimentally demonstrated. Next, it was assumed that the rate of charge transfer in proteins and DNA decays exponentially with the linear distance separating the electron donor and acceptor, and attempts were made to characterize the corresponding exponents. Having obtained widely varying exponents in the case of both media, the corresponding investigators came to the same conclusion, namely, that the coupling pathway strength, and thus the structure and dynamics of intervening medium, rather than the linear distance between donor and acceptor, is that which defines the rate of charge transfer. Finally, it is currently believed that long-distance charge transfer in proteins and DNA occurs by the same mechanism involving a mixture of unistep superexchange tunneling and thermally activated multistep hopping [13,41-43].

Among the four DNA base pairs, guanine has the lowest oxidation potential [44]. At the same time, GG and GGG sequences have lower oxidation potentials than single guanines [45]. Thus, the electron holes generated in DNA by oxidative species are expected to rapidly migrate over long molecular distances by DNA CT and to equilibrate at guanines in GG islands (on a ps/ns timescale) before the slow, irreversible oxidation process leading to the formation of stable base oxidation products, such as 8-oxo-guanine, takes place (on a ms timescale) [46]. Indeed, using a variety of well-defined oxidants and experimental systems, the accumulation of guanine radicals at the 5'-Gs of GG and GGG sequences through long-range DNA CT has been demonstrated in multiple studies *in vitro*, in the nuclei of living cells, and in mitochondria, both in the presence and absence of DNA-binding proteins [41,47,48]. In fact, 5'-G reactivity at a GG site is now considered to be a hallmark of long-range CT chemistry, whereas nonspecific reaction at guanine bases suggests the involvement of alternative chemistry [41,49]. Because guanine radicals are the first products of oxidative DNA damage in the cell, DNA CT may drive the non-uniform distribution of oxidative DNA lesions. Pertinently, exons have been found to contain approximately 50 times fewer oxidation-prone guanines than introns. This means that coding sequences may be protected from oxidative DNA damage by DNA CT, which funnels guanine radicals out of exons into introns [50,51].

Importantly, DNA-mediated charge transfer enables long-range communication and long-distance redox chemistry both between DNA and proteins and between individual proteins bound to DNA [40,52,53]. DNA-interacting proteins that induce little structural change in DNA upon binding do not interfere with DNA CT [54], whereas proteins that distort base stacking, flip out bases, or induce DNA kinks (as do certain DNA repair enzymes, methylases, and transcription factors) either block or greatly impede charge transfer along DNA [55,56]. Redox-active DNA-binding proteins can be oxidized and reduced from a remote site through DNA CT. As an example, using DNA as a conducting medium and their iron-sulfur clusters ([4Fe-4S]^2+/3+^) as redox-active centers, the base excision repair enzymes MutY and Endonuclease III of *Escherichia coli *can quench emerging guanine radicals from a distance and communicate among each other when bound to DNA [40,52]. As another example, one-electron oxidation of the iron-sulfur cluster ([2Fe-2S]^1+/2+^) in SoxR, a bacterial transcription factor and a sensor of oxidative stress, leads to the activation of SoxR transcriptional activity, which in turn, initiates a cellular response to oxidative stress. The DNA-bound, reduced form of SoxR is transcriptionally inactive but can be activated from a distance through DNA CT. It has been proposed that, upon oxidative stress, emerging guanine radicals rapidly migrate to areas of low oxidative potential, such as guanine multiplets, which are found in abundance near the SoxR binding region [57], and, by oxidizing SoxR, activate cellular defensive responses [58]. The redox-responsive transcription factor p53, a central regulator of cellular responses to genotoxic stress in higher organisms, can be oxidized through DNA CT and induced to dissociate from its binding sites from a distance. p53 contains 10 conserved cysteines in its DNA-binding domain, and in this case, sulfhydryl (-SH) groups play the role of redox-active centers. Interestingly, the DNA-mediated oxidation and ensuing dissociation of p53 appear to be promoter-specific, adding yet another layer of complexity to p53 regulation [53].

Altogether, it appears that genomic DNA may in fact function as a giant sponge that absorbs oxidizing equivalents and redistributes them within the DNA medium in a spatiotemporally organized and sequence-dependent manner. This conclusion is consistent with a recent discovery indicating that genomic DNA is maintained in the cell as a sponge-like fractal globule [59]. As implied in the works of Leonardo da Vinci [60] and Mandelbrot [61], and as suggested explicitly by West, Brown, and Enquist [62,63], fractal geometry is a telltale sign of a distribution system that manages the transport and exchange of energy/matter under the pressure for economic efficiency [8].

Complementing the findings on electron transport within proteins and DNA, studies on proton dynamics at protein-water and lipid-water interfaces demonstrate that the surfaces of proteins and biological membranes, together with the ordered molecules of interfacial water, can act as proton-collecting, -storing, and -conducting media [64-69]. The capture of protons from the bulk aqueous phase and the transport of protons on the surfaces of dense macromolecular media are mediated by the judicial spatiotemporal organization of protonatable groups and ordered molecules of interfacial water. Molecular ordering of water at the surfaces of proteins and lipid membranes facilitates the lateral transfer of protons along the surface, while creating a kinetic barrier for proton exchange between the surface and the bulk phase. As a result, the rates of lateral proton transfer along macromolecular surfaces exceed the rates of proton exchange with the bulk phase by orders of magnitude, enabling the efficient capture and transport of protons on the surfaces of proteins, lipids, and their complexes [64,67,70].

In proteins, negatively charged residues such as that of aspartate and glutamate (p*K*_*a *_in water ~4.0) serve to attract and pass protons along protein surfaces, whereas surface-exposed histidines residing among acidic groups (p*K*_*a *_~ 7.0), which often decorate the orifices of proton-conducting channels/pathways, function to trap and to store protons, feeding them into proton pathways/sinks [64,67]. Similarly, low-p*K*_*a *_head groups of lipids are proposed to mediate the capture and transport of protons on biological membranes, whereas high-p*K*_*a *_lipid groups are used for buffering and guiding proton fluxes into proton sinks [64,66]. Moreover, most biological membranes contain anionic lipids, with phosphate, sulfate, or carboxylate groups forming so-called acid-anions. The physicochemical properties of acid-anions make them an ideal means to capture, store, and transport protons (as well as other ions) on polyanionic surfaces (for details, see [65,66]). Altogether, studies on proton dynamics at lipid-water interfaces suggest that biological membranes can act as efficient proton-collecting and -distributing systems that increase the effective proton (ion) collision cross-section and provide an appropriately structured molecular platform that enables the harvesting, dynamic storage, and organized transport of protons (and other ions) on large macromolecular surfaces. Such an arrangement would be an ideal means to ensure stable yet flexible and adaptive procurement, distribution, and supply of protons (ions) in conditions of the constantly fluctuating and changing demands from proton (ion) consumers such as receptors, channels, enzymes, and other proteins and multiprotein complexes functioning in association with lipid membranes.

It should be pointed out that, within dense media composed of biological macromolecules and interfacial water, electrons and protons rarely, if ever, move independently, meaning that the fluxes of electrons and protons are often, if not always, conjugated. Enzymes, for example, commonly rely on the coupling of electrons and protons to perform chemical transformations. Amino acid radical initiation and propagation, small molecule activation processes, as well as the activation of most substrate bonds at enzyme active sites all involve the coupling of electron transfer to proton transport [37,71]. The tunneling of hydrides or hydrogen atoms is an obvious example of proton-coupled electron transfer (PCET) [72,73]. However, theoretical and experimental studies indicate that, to be coupled, electrons and protons do not necessarily have to move along collinear coordinates. Electron and proton fluxes remain coupled as long as the kinetics and thermodynamics of electron movement is dependent on the position of a specific proton or a group of protons at any given time. Thus, electron transport to and from active sites can occur in concert with protons hopping "orthogonally" to and from active sites along amino acid chains or structured water channels [30,71,74,75]. Redox-driven proton pumps (e.g., cytochrome *c *oxidase), monooxygenases (e.g., cytochrome P450), peroxidases, and hydrogenases are examples of enzymes employing orthogonal PCET [71]. Importantly, proton-coupled electron transfer processes are not limited to proteins and have been observed experimentally and in simulations in DNA and DNA analogs [76-79]. Experimental evidence suggests, for example, that electron transfer in duplex DNA is coupled to interstrand proton transfer between complementary bases [80,81].

To summarize, a large body of experimental evidence demonstrates that proteins, nucleic acids, lipids, and their complexes represent structured macromolecular media that enable and facilitate the capture and directed transport of electrons and protons. Because so many physicochemical properties of proteins, nucleic acids, and lipids appear to have been carefully tailored by evolution to satisfy the requirements of organized electron transport over large molecular distances, it is reasonable to suggest that electron flow may represent a fundamental physical force that sustains, drives, and informs all biological organization and dynamics.

Indeed, from a larger-scale perspective, the structures and dynamics of all aerobic organisms are sustained and fueled by a continuous and rapid flow of electrons and protons passing through their internal structures, with foodstuffs and water serving as sources of electrons and protons, and oxygen and biosynthesis being their major sinks. Using the energy of the sun, photosynthetic organisms drive the flow of electrons and protons in the opposite direction, from reduced oxygen in the form of water and carbon dioxide back into foodstuffs, thus completing and fueling this global reduction-oxidation cycle. Although different organisms (or the same organisms under different circumstances) may use different chemical species as sources of and sinks for electrons and protons, what appears to be always and everywhere present is a continuous and rapid flow of electrons and protons passing through each and every living organism.

Pertinently, hydrogen (i.e., a bound state of a proton and an electron) is the most abundant chemical element in the universe, making up 75% of normal matter by mass and over 90% by number of atoms, while life on Earth has evolved in a continuous flux of cosmic radiation consisting of protons (~90%), alpha particles (i.e., two protons and two neutrons; ~9%), and electrons (~1%) [82,83]. Hydrogen is the third most abundant chemical element on the Earth's surface, captured largely in the form of hydrocarbons and water [83]. Notably, anaerobic chemolithoautotrophs, archaebacteria that obtain energy from inorganic compounds and carbon from CO_2 _and that are believed to be one of the earliest organisms evolved on the planet, acquire their energy either by producing methane (CH_4_) from carbon dioxide and hydrogen or by producing hydrogen sulfide (H_2_S) from sulfur and hydrogen [84,85]. In other words, these microorganisms capture, store, and distribute hydrogen over the planet's surface, for hydrogen as a gas escapes Earth's gravity and is lost to space if not captured in a chemically bound form.

Before further discussion of the experimental evidence demonstrating a key role for electron and proton flow/circulation in biological organization and dynamics, let us pause for a moment and reconsider the aforementioned studies on electron and proton transport in biomolecular media within the framework of nonequilibrium thermodynamics.

### A nonequilibrium model of biological organization and dynamics

Whether explicitly stated or tacitly implied, the phenomena studied in molecular and cell biology are traditionally interpreted and rationalized within the conceptual framework of classical physics, i.e., classical mechanics and *equilibrium *thermodynamics. This tradition is a direct consequence of the institutionalized nature of science, combined with the fact that molecular and cell biology and the corresponding institutions were founded and directed by physicists and biochemists whose mental structures and, thus, habitual interpretations were shaped by their rigorous training in classical physics and engineering. Accordingly, virtually all of the studies mentioned in the previous section were performed and interpreted using the concepts, assumptions, and theories of classical physics, despite the commonly accepted fact that the cell/organism (any living organization, in fact) is an open *nonequilibrium *system, which exists and functions only because of the incessant flow of energy/matter passing through it. Therefore, it is reasonable to suggest that the aforementioned studies, which have been performed by taking a nonequilibrium system of conjugated fluxes and gradients, destroying fluxes and gradients, isolating individual components, placing them in equilibrium conditions, making averaging measurements, and inferring the original state of the system with the help of the theories and assumptions pertaining to the equilibrium state, may interpret reality neither accurately nor completely. Indeed, the reinterpretation of these studies and their conclusions within the framework of nonequilibrium thermodynamics reveals a qualitatively different image of reality.

**Figure 2:**
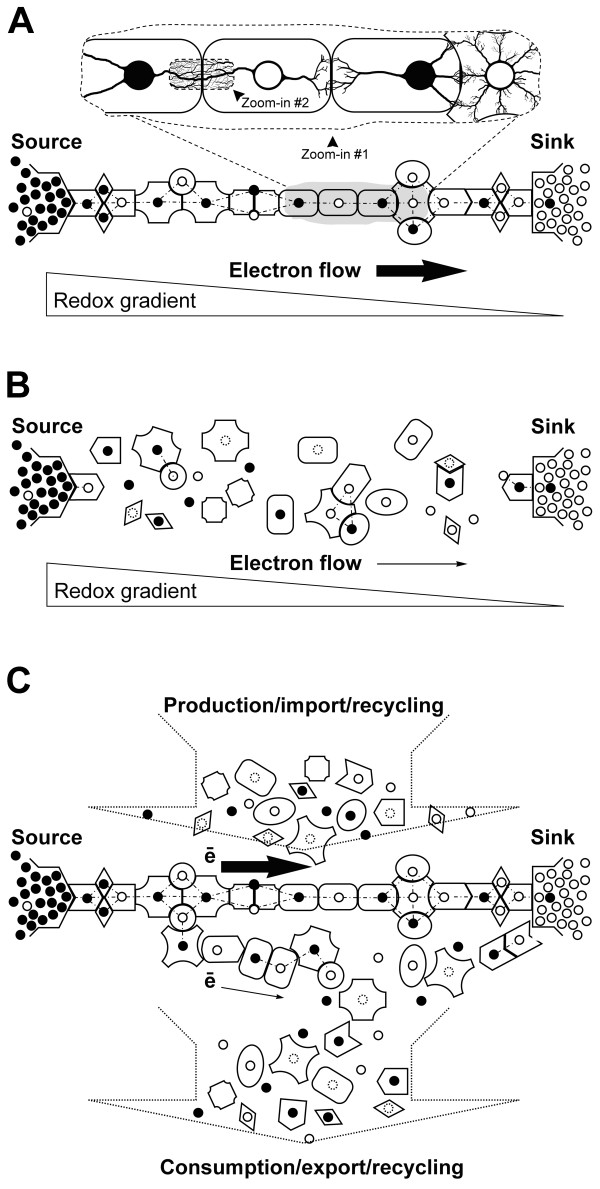
**A linear, nonequilibrium model of biological organization and dynamics**. The SOFT-NET theory conceptualizes biological organization and dynamics in terms of nonequilibrium electron transport chains that support and, at the same time, are supported by electrons moving between redox centers along electron gradients. Electron flow/circulation is organized by and, at the same time, organizes intervening macromolecular media residing in an aqueous environment (see details in the text). Two major forms of electron transport and the corresponding organization of a linear electron transport chain are shown: **A**) fast electron transport through and by means of highly organized macromolecular media (e.g., proteins, lipids, nucleic acids, and their complexes) and **B**) relatively slow electron transport by means of the same disorganized chain components diffusing in the aqueous phase. Two consecutive "zoom-ins" (in **A**) reveal the multiscale complexity of alternative and, thus, competing pathways of electron flow. The apparent complexity is greatly simplified, however, by the fact that electron flow is organized in a self-similar (i.e., scale-invariant) manner, with pathways making up higher-order pathways making up yet higher-order pathways and so forth. Within each hierarchical level in the organization of electron flow, individual pathways are similarly clustered into families of related pathways, with the overall electron flow being dynamically, competitively, and highly unevenly partitioned among alternative pathways and pathway families. **C**) The model is brought closer to reality by introducing orthogonal flow of chain components passing through a steady-state organization of the electron transport chain. Filled (●) and empty (○) circles denote redox centers with excess and deficit of electrons, correspondingly. Dotted line (-·-·-·) denotes electron transfer. Geometrical shapes with complementary features are animate media.

As a simple nonequilibrium model of biological organization and dynamics, consider a linear electron transport chain made of redox-active centers connected via what can be called "environmentally responsive, structurally adaptive, and proactive media" or, simply, "animate media" (Figure 2). The term "animate media" is meant to signify that a medium connecting redox centers can adopt multiple alternative conformations, with each conformation having multiple electron-conducting pathways, and that alternative conformations interconvert under the combined influence of the environment and the internal state of the medium. Redox centers and intervening animate media reside in an aqueous environment, sandwiched between a source of and a sink for electrons. In far-from-equilibrium conditions, a given electron-conducting chain made of redox-active centers and animate media mediates and, at the same time, is stabilized/sustained by the flux of electrons moving from the source through the chain into the sink.

One can immediately appreciate that, in far-from-equilibrium conditions, in addition to or even instead of the difference in redox potentials between individual redox centers, the electron gradient becomes a key force driving electron flow. When large enough, an electron gradient may drive electron transport through a chain of equipotential redox centers and even through energetic "bumps" along an electron transport pathway. In other words, the requirements for fine control of reaction driving forces and the precise structuring of the intervening medium can be relaxed in far-from-equilibrium conditions, as compared to the equilibrium state, given the existence of appropriate gradients and fluxes. In addition, thermally driven electron transfer is expected to be much more efficient in far-from-equilibrium conditions, where vibrational modes of a conducting medium can be highly structured and coordinated. Consequently, large-scale, organized electron flow becomes much more feasible in far-from-equilibrium conditions, as compared to the equilibrium state.

It should be kept in mind that, in far-from-equilibrium conditions, there is a never-ending competition between alternative electron-conducting pathways and alternative conformations within each of the intervening animate media. Which pathways and conformations are actually preferred (i.e., selected and stabilized) within individual animate media will depend both on the environment and on the internal state of a given conducting medium. It is fair to assume that, in a stable environment, those pathways and conformations that are optimized in terms of ET efficiency will tend to prevail and to persist longer than their less efficient alternatives.

Let us next consider the relationship between the degree of order within a nonequilibrium electron transport chain and the rate of energy/matter flux passing through the chain. On one hand, efficient and rapid flow of energy/matter through a semi-structured adaptive medium requires an adequate and stable spatiotemporal ordering of the medium, which can be achieved, for example, by stabilizing one or a few appropriate conformations selected from multiple competing alternatives. On the other hand, the higher the degree of spatiotemporal order in a medium, the more energy required to sustain this order, i.e., the faster the energy/matter flux needed. As a result, in far-from-equilibrium conditions, the rate of energy/matter flux passing through a structurally adaptive medium and the degree of spatiotemporal order of the medium are co-defining and will tend to change in parallel, in an all-or-none manner in the form of organizational state transitions. Therefore, it is reasonable to suggest that the different mechanisms invoked to explain the seemingly conflicting experimental data on electron transfer in proteins and DNA (see discussions of the corresponding controversies in [32,41]) can be readily reconciled as complements within the framework of nonequilibrium thermodynamics. In other words, different mechanisms of electron transfer are not mutually exclusive in nonequilibrium conditions but, instead, may co-exist, compete, cooperate, or be suppressed or enhanced, depending on the circumstances.

For example, relatively slow and unorganized electron transport inside living cells may take place simply via free diffusion of soluble electron donors and acceptors, such as reactive species of oxygen, nitrogen, carbon, sulfur, and other chemical elements, as well as NAD(P)H, glutathione, iron, hydrogen, sulfate, nitrate, fumarate, redox-active proteins, and many other species. Moderately fast and organized electron transport, which requires and, at the same time, supports a moderately organized medium, may take place in the form of electrons hopping along preferred electron-conducting pathways between redox-active centers embedded within dense macromolecular structures. A supercurrent, i.e., an even faster and more organized electron flow, will require and, at the same time, sustain a yet greater degree of spatiotemporal molecular order. In other words, in nonequilibrium conditions, the same set of redox centers and intervening animate media can mediate electron flow by a variety of mechanisms. Consequently, the electron transport chain shown in Figure 2, as well as macromolecular complexes, sub-cellular structures, and the cell as a whole, can behave as insulators, as semiconductors, and perhaps as high-temperature superconductors, depending on the degree and adequacy of spatiotemporal order in their internal organization and dynamics. Such an interpretation of cellular organization and dynamics may explain the paradoxically high densities of biological macromolecules maintained in living cells (estimated 300-400 mg/ml of proteins and RNA alone [86]). One can also infer that the sub-cellular structures and organelles containing relatively higher densities of proteins, nucleic acids, and/or lipids, such as lipid rafts, post-synaptic densities, mitochondria, and the nucleus, are the areas of relatively higher electron densities, faster electron fluxes, and higher degrees of molecular coordination and order. Of note, such structures tend to have higher affinities for osmium tetroxide, a powerful and highly toxic oxidant widely used for cross-linking and staining of biological specimens for transmission electron microscopy. Consequently, many such structures appear as dark, electron-dense regions on electron micrographs.

It is worth pointing out that because the degree of order and the rate of energy/matter flow are co-defining in far-from-equilibrium systems, large-scale conductivity is an emergent property of organization (an ordered whole) rather than of component parts. Properties of parts are only compatible with and, in fact, are often selected and/or reinforced by the emergent properties of the organizational whole. Needless to say, many essential properties of both parts and the whole disappear every time an organization is destroyed due to the isolation and characterization of its individual components. In addition, any living organization is more than a simple sum of its components, and the properties and capabilities of the whole are defined not only by the properties and capabilities of its parts but also by a particular *organization *of relationships maintained between constituent parts. Consequently, the same set of parts can and generally will give rise to a diverse set of alternative organizational wholes that may differ widely in their individual properties, attributes, and capabilities. Multiplicity of alternative organizational wholes made of the same parts may explain the rapid divergence of individual properties, attributes, and behaviors commonly observed in isogenic populations of proteins, cells, and organisms, and discussed in biological literature under the term "stochasticity" [87,88].

Next, let us assume that the electron transport chain in our model is maintained in one of its highly conductive, and thus highly ordered, states and begin to gradually slow down the flow of electrons (energy/matter, generally speaking) passing through the chain. When the rate of flux reaches a certain threshold value, the most costly animate medium (i.e., the least adequate under the given circumstances) in the chain will relax to its less conductive state. There are two most likely outcomes of such a failure. Because the impaired conductivity of a part impairs the overall flow through the system, a failure of one part may precipitate an avalanche of structural relaxations in other parts, bringing the whole chain down to an organizational state of a lower degree of order and, thus, of conductivity. Alternatively, having transiently acquired greater conformational flexibility, a relaxed part may quickly find and adopt a less costly conformation (i.e., one more economically efficient and more adequate under the circumstances), thus keeping pace with and sustaining the energy/matter flow or perhaps even improving it. In other words, the chain as a whole and each of its parts are adaptive to some degree and will generally tolerate fluctuations to some extent. Although small fluctuations may precipitate great avalanches, most of the time small fluctuations will cause only local relaxations and restructuring. Whereas large fluctuations can be tolerated, the most likely outcome of a large fluctuation will be a large-scale relaxation and restructuring. Note that the adaptability of the whole is built upon and depends upon the adaptability of its individual parts and that the adaptations of a part or the whole invariably involve local or global organizational relaxation and restructuring, caused by and, at the same time, causing fluctuations or changes in the overall energy/matter flow.

There is a special situation in the dynamics of an electron transport chain that should be emphasized, as it is of special importance for biological organizational dynamics in general: the case when all or most of the individual animate media comprising the chain approach their relaxation thresholds more or less simultaneously (i.e., all or most of animate media are synchronized). The whole system is then poised at the threshold of a large-scale organizational transition; the system becomes critical. When a system is critical, infinitesimally small fluctuations, either external or internal, may trigger an all-or-none cooperative response of the whole in the form of a large-scale organizational transition. Notice that the same is also true if we approach the critical state from the opposite direction, i.e., if instead of slowing down energy/matter flow, we accelerate it, forcing all or most of the animate media into their more organized and thus more conductive states. In other words, independent of the direction from which a system approaches its critical state, the system becomes most sensitive and powerful when it is critical, behaving and responding as one.

In the preceding example, we ignored environmental influences and were changing the internal state of the electron transport chain by changing the intensity (the flow rate) of the energy/matter flux passing through it. Let us now fix the rate of energy/matter flow and vary environmental conditions instead. Due to the reciprocal relationship between the rate of energy/matter flow and the degree of order in far-from-equilibrium systems, the responses of individual animate media and the chain as a whole to environmental fluctuations will be similar to those described above for internal fluctuations. The environmental changes that bring about conformational transitions impairing conductivity will either lead to rapid structural adaptation of the affected animate media or to cascading failures within the chain. Analogously, if and when the electron transport chain becomes critical, it becomes exceptionally sensitive to external influences, behaving as one and responding to the environment by cooperative organizational transitions in an all-or-none manner.

Next, let us consider the situation when, due to a major external or internal perturbation, an electron transport chain dissociates into its individual components (Figure 2B). Immediately after dissociation, reduced and oxidized redox centers, whether alone or in cooperation with animate media, will continue to transport electrons down the electron gradient in a relatively slow and disorganized manner, via random collisions, electron transfer, and diffusion. However, a new electron transport chain(s) will soon emerge, enforced by the electron gradient, facilitated by the electrostatic attraction between charge transfer complexes, and shaped by the competition for electron flow between alternative arrangements of the electron transport chain. The waiting time can be significantly shortened if individual components making up the chain have specific structures that allow them to recognize and bind each other in an ordered manner. Adding scaffolds that facilitate a proper spatial arrangement of individual components into a functional chain can shorten the waiting time even more.

Finally, in analogy to the incessant synthesis/import and degradation/export of cellular constituents in a living cell, let us include into our model orthogonal flow of individual chain components that continuously pass through the system (Figure 2C). Let us also assume that the probability of the elimination of a free, unattached component (e.g., due to degradation and/or export) is significantly higher, and thus its life expectancy within the system is considerably lower than the corresponding probability of the same component when it is employed within an active electron transport chain.

It is not difficult to see that the outlined nonequilibrium model of electron flow through a structurally adaptive medium that continuously turns over will have the following basic properties. Different environments will favor different electron transport chains. Environmental and internal changes and fluctuations in energy/matter flow will drive the emergence, evolution, and adaptation of electron transport chains. Upon local relaxation events, individual chain components will compete for transiently opened vacancies within existing chains, consequently improving or undermining the chains they join. Following global relaxation, multiple alternative electron transport chains will initially compete, until one or a few chains, which are optimized for rapid and efficient electron transport under the given environmental circumstances, take over the electron transport and win the competition. As a whole, the system of electron transport will co-evolve with its environment. Generally speaking, the electron transport chains that form fastest and that are flexible and adaptive, yet stable and efficient, will persist longer, thus giving rise to the increasingly stable electron-conducting chains that maximize electron flux, while minimizing the energy expenditure required for their maintenance. Because the life expectancy of chain components is coupled to the life expectancy and stability of their host chains, both the life expectancies of dominating chains and the life expectancies of their components will tend to increase in parallel in stable environments. If, with time, a given environmental niche becomes homeostatic as, for example, do many intracellular and intraorganismal environments, the economic efficiency of an electron transport chain operating in a stable environment can be improved by synthesizing only those chain components that have proven to perform adequately in a given environment, while suppressing the synthesis of irrelevant components, in a manner of the processes underlying cell differentiation and, in fact, any functional specialization.

Altogether, it is evident that even an extremely simplified nonequilibrium model of electron transport through a structurally adaptive, multicomponent medium faithfully captures most of the basic features of biological organization and dynamics. Of course, the model can be further improved and brought closer to reality by considering three-dimensional networks of electron transport; multiple sources of and sinks for electrons; competition and cooperation among networks, chains, pathways, sources, and sinks; by introducing and considering various conjugated fluxes, such as those of protons, ions, phosphate, ATP, metabolites, and other species; by introducing energy/matter transformations, and so forth. However, the main goal of our discussion is not to attempt to model the living organism in the conventional, mechanistic sense, trying to account for the infinity of the continuously changing interdependent parts, influences, and processes that make up the living organism, but instead to re-focus our attention on the scale-invariant organizational principles, concepts, and processes that, by virtue of their scale-invariance, collapse the infinity of largely irrelevant details into a manageable number of essential categories, variables, and their relationships.

The reinterpretation of biological organization and dynamics in terms of electron transport chains and networks that support and, at the same time, are supported by electron flow (as outlined in Figure 2) suggests, for example, that it may be worth identifying those constituents of living cells that fall into the conceptual categories of redox-active centers, electron transport pathways, animate media, and electron sources and sinks. In addition, it may be also informative and revealing to identify and analyze physical manifestations of local and global organizational relaxations and transitions in living cells as well as their effects on the energy/matter flow through biological media.

### Redox centers and electron relays in living cells

Because of their specific physicochemical and structural properties, as discussed above, proteins, nucleic acids, and lipids are obvious candidates for the role of the adaptive, animate media that enable, mediate, and organize electron transport between redox-active centers. Let us therefore consider the chemical species that are either known to function or can potentially function as redox-active centers and/or components of electron relays within the macromolecular organization of the cell.

*Transition metals*, which can readily alternate between different oxidation states by donating and accepting electrons, are well-known redox-active centers in the cell. Transition metals are actively imported and retained by all living cells. Studies on transition metals in bacteria show that individual bacteria accumulate transition metals at concentrations that are several orders of magnitude higher than that found in growth media. The typical concentrations of transition metals in *E. coli *are estimated to be approximately 0.1 mM for Zn and Fe; 10 μM for Cu, Mn, and Mo; and lower values for V, Co, and Ni [89]. Importantly, the abundance of transition metals in the cell is matched by the abundance of metalloproteins, which comprise about a third of all structurally characterized proteins.

In their free form, transition metals such as iron and copper can be readily oxidized and reduced in the cytoplasm by a variety of species, thus enabling diffusion-driven electron transport (e.g., via production of diffusible free radicals and other reactive species). The Fenton reaction and associated reactions are examples of redox reactions mediated by iron in aqueous solutions [90]:

(1) Fe^2+ ^+ H_2_O_2 _→ Fe^3+ ^+ •OH + OH^-^

(2) Fe^3+ ^+ H_2_O_2 _→ Fe^2+ ^+ O_2_•^- ^+ H^+^

(3) 2O_2_•^- ^+ 2H^+ ^→ H_2_O_2 _+ O_2_

(4) •OH + H_2_O_2 _→ H_2_O + H^+ ^+ O_2_•^-^

(5) O_2_•^- ^+ Fe^3+ ^→ Fe^2+ ^+ O_2_

(6) •OH + Fe^2+ ^→ Fe^3+ ^+ OH^-^,

where •OH is a hydroxyl radical and O_2_•^- ^is a superoxide anion.

Most of the time, however, transition metals are transported and incorporated into proteins in an organized manner, as redox-active elements of iron-sulfur clusters, heme groups, and other cofactors. Redox-active prosthetic groups with multiple oxidation states are present in a wide variety of enzymes and proteins, such as ferredoxins, dehydrogenases, hydrogenases, oxidases, reductases, oxygenases, cytochromes, and blue copper proteins. In fact, clusters of nonheme iron and inorganic [Fe-S] clusters are some of the most ubiquitous and functionally versatile prosthetic groups in nature. More than 120 distinct types of enzymes are known to contain [Fe-S] clusters [91]. The ability to delocalize electron density over both Fe and S atoms makes [Fe-S] clusters ideally suited for mediating electron transport [91,92]. Another popular arrangement used in biomolecular electron relays is a conjugated, often ring-based, system of covalent bonds with delocalized electrons, which is frequently positioned near a metal ion(s) and acts as a complex redox-active center and/or an electron relay element with multiple oxidation states.

*Riboflavin *(vitamin B_2_) is an essential micronutrient that plays a key role in energy metabolism. It is required for the metabolism of fatty acids, ketone bodies, carbohydrates, and proteins. Riboflavin is the central component of the cofactors flavin adenine dinucleotide (FAD) and flavin mononucleotide (FMN) and is therefore required by all flavoproteins. Flavins can act as oxidizing agents through their ability to accept a pair of hydrogen atoms. Reduction of the isoalloxazine ring yields the reduced forms of flavoproteins (FADH_2 _and FMNH_2_). Flavoproteins exhibit a wide range of redox potentials and play various roles in intermediary metabolism [93].

In fact, a large variety of *prosthetic groups *and *cofactors*, which are produced from *vitamins*, *micronutrients*, and *metabolites*, are known to mediate electron transfer reactions. Examples include, but are not limited to, iron-sulfur clusters, heme groups, NAD(P)^+ ^(niacin/vitamin B_3_), lipoamide (lipoic acid), cobalamins (vitamin B_12_), menaquinone (vitamin K), ascorbic acid (vitamin C), α-tocopherol (vitamin E), retinol (vitamin A), coenzyme A, coenzyme Q, *S*-adenosylmethionine, and pterins. In addition, such biologically ubiquitous and abundant families of chemical species as *porphyrins*, *quinones*, *polyphenols*, and *pigments *commonly function as redox-active centers and/or components of electron relays. Porphyrin is a large heterocyclic organic ring and a central functional element of hemoproteins. The delocalized π-electrons of porphyrin endow it with the ability to mediate electron transfer. Oxidized and reduced quinones are universally used for shuttling electrons in electron transport chains and are common constituents of biologically active molecules, both natural and synthetic.

Biological pigments such as *chlorophylls*, *melanins*, *carotenoids*, and *flavanoids *are a special case, because, in addition to mediating redox reactions, pigments can capture and convert radiation energy into charge movement. Consider melanin, an ancient pigment found in all biological kingdoms, as an example [94]. In humans, melanin is present in skin, hair, the brain, the nervous system, the eye, the adrenal gland, and the inner ear. A heterogeneous aggregate of π-stacked oligomers made of indolequinone units, melanin has a number of remarkable and poorly understood physicochemical properties. Melanin acts as an amorphous semiconductor. It has broad-band monotonic absorption from the far UV into the infrared, atypical for organic chromophores. Melanin gives a persistent electron spin resonance (ESR) signal, a clear indication of free radical centers present in the material. Melanin can quench radical species as well as produce them. Melanin dissipates all sorts of absorbed radiation in a non-radiative manner through an efficient but rather mysterious process (for reviews on melanin, see [94-96]). Melanin participates in electron transfer reactions, reducing and oxidizing other molecules. A key monomer of melanin has been reported to perform photon-driven proton transfer cycles [97]. Melanin exhibits strong electron-phonon coupling and is one of the best sound-absorbing materials known [98]. Melanized fungi, which thrive in such extreme environments as the damaged reactor in Chernobyl and orbiting spacecraft, are actually stimulated to grow by ionizing radiation and exhibit "radiotropism," i.e., directional growth towards sources of ionizing radiation. Interestingly, many fungal fossils appear to be melanized [99]. Consequently, it has been proposed that melanin may function as a broad-band radiation energy harvester, in a manner similar to chlorophyll [100].

Two points should be emphasized here. First, the involvement of many, perhaps most, prosthetic groups and cofactors in electron transfer reactions has been discovered and elucidated fortuitously, since the conventional biological paradigm provides no rationale for systematic investigation of the redox (electronic) properties of cellular constituents. Second, in the same way as the p*K*_*a *_of an isolated amino acid and the p*K*_*a *_of the same amino acid embedded within protein matrix may differ dramatically, the redox behavior of chemical species isolated in the test tube may differ drastically from the redox properties of the same species in their natural microenvironments, as electronic configurations of the same species are generally different in different microenvironments. In other words, the fact that "well-studied" chemical species and macromolecules are regarded as redox inactive may simply mean that the corresponding measurements were performed using inappropriate experimental conditions. A characteristic example is the MutY and Endonuclease III glycosylases from *E. coli*. Their iron-sulfur clusters had been assumed to be redox inactive until researchers decided to test their behavior in DNA-bound enzymes [40]. Therefore, it is reasonable to suggest that most, perhaps all, prosthetic groups and cofactors function in reality as essential components of redox-active centers and/or electron relays and that the main reason why all organisms continuously ingest vitamins and micronutrients is to ensure an incessant supply of electronically active chemical species that are required for the production, maintenance, and turnover of redox-active centers and electron relays within the steady-state molecular organization of the cell/organism.

Whereas many characterized redox-active proteins contain cofactors such as metals, NAD^+^, and FAD, *thiol-based redox systems *are perhaps the most common and versatile mediators of electron and proton flow in proteins, thanks to the remarkable chemical versatility of sulfur, which can participate in several mechanistically distinct redox reactions, such as nucleophilic attack, and electron, hydrogen (proton, atom, and hydride), and oxygen atom transfers. Sulfur occurs in up to ten different oxidation states *in vivo*, most often in such forms as thiols (-SH), thiolates (-S^-^), thiyl radicals (-S•), disulfides (-S-S-), sulfenic (-SOH), sulfinic (-SO_2_H), and sulfonic acids (-SO_3_H), disulfide-*S*-oxides (-SOS-), and other species. Accordingly, sulfur-containing compounds mediate diverse cellular processes related to electron/proton transfer and storage. The amino acid *cysteine*, for example, performs a wide variety of tasks in proteins, such as disulfide formation, metal binding, electron donation, hydrolysis, and redox catalysis (for reviews, see [101-103]). One well-known redox reaction involving cysteine is reversible disulfide formation. The low redox potential of cysteine allows for efficient electron transfer from cysteine, leading to thiyl radical and disulfide formation. The reverse reaction involves disulfide bond reduction to two cysteine thiols by the transfer of electrons from electron donors such as NADPH, FADH_2_, and glutathione. Sulfhydryl groups (-SH) in proteins can thus function as donors (reduced state) and acceptors (oxidized state) of electrons and protons, i.e., as redox-active centers and/or constituents of electron and/or proton relays.

*Glutathione *(γ-glutamyl-cysteinyl-glycine, GSH) is the most abundant low-molecular-weight thiol in animal cells, and GSH and glutathione disulfide (GSSG) constitute a major redox couple. Under normal physiological conditions, animal cells typically contain 0.5 to 10 mM GSH, with the GSH/GSSG ratio > 10 [104,105]. Whereas the reduction of disulfide bonds in proteins by free GSH and other reducing equivalents occurs in a relatively slow and undirected manner, thioredoxin, glutaredoxin, and other enzyme-based redox systems provide speed and direction to thiol-mediated redox reactions, thereby accelerating and structuring electron and proton fluxes in the cell (for reviews, see [106,107]). Indeed, a large body of experimental evidence indicates that thiol-mediated redox reactions and the corresponding electron and proton fluxes are highly structured and compartmentalized in living cells. For example, oxidation of OxyR, a transcription factor responsible for the expression of antioxidative genes in *E. coli*, occurs in response to as little as 5 μM hydrogen peroxide, whereas the redox state of glutathione remains unchanged in cells challenged with 200 μM H_2_O_2 _[108]. As another example, low-intensity light triggers oxidation of the chloroplast RB60 protein, a constituent part of a photoresponsive complex regulating translation, whereas other proteins with reactive thiols remain unaffected [109].

*Radicals *and *antioxidants *are chemical species that can act as redox-active centers and/or constituents of electron relays in living cells and organisms. Broadly defined, radicals are any species with unpaired electrons, whereas antioxidants are any species that act as electron donors for oxidizing species, yielding less reactive products in the process. Both radicals and antioxidants can be useful and harmful for the cell, depending on the circumstances. In organized molecular contexts, radicals such as reactive oxygen species, reactive nitrogen species, amino acid radicals in proteins, base radicals in nucleic acids, and carbon and hydroperoxyl radicals in lipids may play positive roles as electron donors, acceptors, and transporters. In conditions of disorganization, when for example, a macromolecular complex/structure mediating organized electron transport suddenly relaxes and/or dissociates into individual components, "freed" radicals may exert their reactivity in an uncontrolled, undirected, and thus, disruptive manner. In the same way, but at the human scale, proactive members of an organization invigorate and drive the organization. As a part of a mob, they inflame chaos and destruction. It is important to realize, however, that in the context of organizational adaptation and evolution, "disruptive" and "destruction" are not necessarily negative terms. When applied to irrelevant, inadequate, or obsolete structures, destruction can be a creative and revitalizing force. In fact, self-destruction and recreation is the only way to keep on living. This fact is reflected in the myth of the Phoenix firebird, a symbol of life. Pertinently, conventional fire results from the rapid oxidation of a fuel (usually a hydrocarbon) by atmospheric oxygen in an uncontrolled reaction mediated by radical intermediates. The physicochemistry of combustion and flames may thus help to explain both the positive and negative aspects of antioxidants. In the same way that both excessive and insufficient quenching of a fire are counterproductive, as the former chokes it and the latter leads to a runaway fire, too much and too little of antioxidants are detrimental for the living fire that, in the form of organized electron flow, animates and powers cells and organisms.

It should be emphasized that electron transport inside living cells takes place in diverse forms that may differ drastically in their degrees of organization and order, and the spatiotemporal scales on which they operate. As an example, let us compare a relatively disorganized and slow propagation of electrons by means of diffusible reactive oxygen species in the bulk phase of the cytoplasm and the exquisitely structured and fast electron transport via transient amino acid radicals in a protein medium.

The use of oxygen as an electron acceptor in living cells is associated with the production of reactive oxygen species (ROS), which exist in many forms. Different ROS vary in their physicochemical properties and lifetimes and often readily interconvert by reacting with other chemical species, including water. The hydroxyl radical (•OH), superoxide anion (O_2_•^-^), singlet oxygen (^1^O_2_*), and hydrogen peroxide (H_2_O_2_) are perhaps the best-studied forms of ROS in living cells [90]. Hydrogen peroxide is not a radical but can easily convert into one (e.g., Eqs. 1, 2, and 4). Hydroxyl radical (•OH) is highly reactive and will react with virtually any molecule in the cell in the immediate vicinity of the site where it is produced. Superoxide (O_2_•^-^) and hydrogen peroxide (H_2_O_2_) are relatively less reactive and, thus, longer-lived. They can diffuse away from the sites of their production and react at distant locales. Under normal conditions, O_2_•^- ^diffuses over short distances only (approximately 0.5 μm, as estimated in [110]), before its dismutation to H_2_O_2 _(Eq. 3). Whereas H_2_O_2 _can cross cell membranes, O_2_•^- ^cannot, unless it passes through a specific channel [90,111]. It should be noted that, although oxygen has a high oxidation potential, ground-state oxygen is a sluggish oxidant, requiring activation (i.e., input of energy) to realize its oxidative potential. Molecules that have their electrons ripped off upon encountering oxidizing radicals often turn into radicals themselves. Such a "contagious" radicalization may lead to radical chain reactions and, as a consequence, propagation of electrons and electron holes. Radical chain reactions are especially likely and rapid in dense macromolecular media such as proteins, nucleic acids, lipids, and their complexes. For example, any species reactive enough to abstract a hydrogen atom may initiate lipid peroxidation through radical chain reactions mediated by carbon and hydroperoxyl radicals in polyunsaturated fatty acids [90]. Pertinently, illumination of unsaturated fatty acids in the presence of chemical species promoting the formation of singlet oxygen, such as chlorophylls, porphyrins, bilirubin, or retinal, initiates rapid lipid peroxidation, and such reactions occur *in vivo *in the mammalian eye and in patients suffering from porphyrias [90,112]. Thus, pigments and vitamins can capture radiation energy and use it to activate oxygen, which then acts as a sink for the electrons moving via transient radicals in dense macromolecular media. If electron propagation is disordered and stochastic, it will be disruptive for existing macromolecular structures and dynamics. If electrons move along organized and structured pathways, they will sustain and power macromolecular organization and dynamics.

Altogether, diffusible forms of reactive chemical species, such as ROS, reactive nitrogen species (RNS), reactive sulfur species (RSS), and other oxidizing and reducing equivalents, represent a relatively unorganized form of electron transport that populates the bulk phase of the cytoplasm and operates on relatively large and slow spatiotemporal scales. Electron transport by means of diffusible electron donors and acceptors is structured and fast insofar as the system of intracellular circulation is structured and fast (see a discussion on the intracellular circulation system in [8] and references therein). Upon encountering dense macromolecular structures (e.g., proteins, nucleic acids, lipids, and their complexes), diffusible radicals and redox-active species may donate to and/or accept electrons from a highly organized and fast electron transport system that exists in the cell in the form of the cytomatrix, the dynamic, sponge-like totality of steady-state macromolecular complexes and subcellular structures in the cell (see a description and discussion of the cytomatrix in [8] and references therein). Since the cytomatrix is essentially a three-dimensional version of the linear electron transport chain shown in Figure 2., the two systems of electron transport in the cell share many of the same components but differ drastically in their respective degrees of order and the characteristic spatiotemporal scales on which they operate. Whereas electron transport in the liquid phase via diffusible species operates mostly on micrometer lengths and on the timescales of microseconds to minutes, electron transport via macromolecular media operates mainly on nanometer lengths and on the femto- to millisecond timescales. As an example of exquisitely structured and fast electron transport in a dense macromolecular medium, consider the propagation of electrons in proteins via transient amino acid radicals.

**Figure 3:**
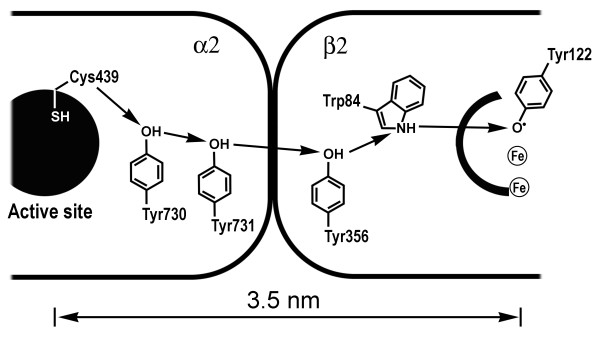
**Electron relay in the class I ribonucleotide reductase (RNR)**. Each enzymatic turnover of RNR is accompanied by the transfer of an electron from the active site cysteine (Cys439) to a long-lived tyrosyl radical (Tyr122) stabilized by a diiron center. Cys439 and Tyr122 reside in different subunits of the enzyme and are separated by a formidable distance of approximately 3.5 nm. Intervening residues (Tyr730, Tyr731, Tyr356, and Trp48) relay the electron by forming transient amino acid radicals and thus function as "stepping stones" for a tunneling electron. The electron relay chain greatly outperforms unistep tunneling in terms of the rate of electron flow it can support. If unistep tunneling alone were responsible for electron transfer from Cys439 to Tyr122, the estimated waiting time for a single ET event would be hours or years. However, a single turnover actually occurs in approximately 200 ms [270].

Studies on electron transfer between experimentally controlled electron donors and acceptors in various peptides and proteins have recently led to the realization that electron transfer over long molecular distances in proteins is often mediated by short- and long-lived radical intermediates, in much the same way as was previously discovered for the DNA medium. Several amino acids, tyrosine and tryptophan in particular, can form relatively stable radicals under physiological conditions. Using spectroscopic methods, the formation of side chain radical intermediates during ET has been documented for a number of proteins and peptides (for a review, see [36]). One well-studied example is the class I ribonucleotide reductase (RNR), in which a long-lived tyrosyl radical (Tyr122) stabilized by complexation with a diiron center is used as a storable oxidizing equivalent (an electron hole) (Figure 3). The active site thiyl radical, situated in one of the RNR subunits, is formed by long-range ET from the active site Cys439 to the tyrosyl radical Tyr122 positioned some 35 angstroms apart in another RNR subunit. A tryptophan (Trp84) and three tyrosines (Tyr356, Tyr730, and Tyr731) situated on a pathway connecting the electron donor and acceptor are oxidized, presumably sequentially, during the ET process [36,113-115]. Importantly, the formation of a long-lived tyrosyl radical requires the presence of molecular oxygen as an electron acceptor, and the same organism (*E. coli*) under anaerobic conditions expresses RNR of a different class, in which a long-lived glycyl radical (Gly681), stabilized by other means, serves as an acceptor for the electron arriving from the active site cysteine (Cys439). The product-derived radical cleaves the sulfur-hydrogen bond of the reduced Cys439 to regenerate thiyl radical in the active site of RNR, thus allowing for enzymatic turnover, redox cycling, and electron transport. The formation of long- and/or short-lived on-pathway radicals during ET has been documented or suggested for a great variety of enzymes. Examples include, but are not limited to, cyclooxygenase [116], galactose oxidase [117], DNA photolyase [118], cytochrome *c *peroxidase [72], pyruvate formate lyase, glycerol dehydratase, and benzyl-succinate synthase [119].

Because any metabolic conversion catalyzed by an enzyme is a segment of a metabolic pathway, which in turn is a segment of a metabolic network, intermediary metabolism as a whole represents and functions essentially as a structured electron transport network, regardless of whether a particular metabolic segment is performed by a multienzyme complex within the cytomatrix or by soluble enzymes in the bulk phase of the cytoplasm. Moreover, considering the nonequilibrium model of biological organization and dynamics shown in Figure 2, it is easy to make a case that, in reality, enzymes catalyze chemical conversions not because they have been designed to do so but because by catalyzing chemical transformations and exchanging the products of these transformations, enzymes obtain and secure a flow of energy/matter - in the form of propagating electrons, protons, electronic and vibrational excitations, and other basic energy/matter forms - that passes through, sustains, and animates their internal organization, and, as a consequences, allows them to survive and to prosper, both as individuals and as complexes and networks (i.e., as organizations). In other words, cellular metabolism can be seen as a self-organizational phenomenon conceptually analogous to the economy at the human scale. This analogy implies that the life of enzymes inside living cells involves an incessant search for and consumption of energy/matter resources as well as choice, competition, cooperation, organization, and economic imperatives, as defined by the struggle for survival, prosperity, and influence. Pertinently, such an image of metabolism immediately explains a panoply of otherwise paradoxical discoveries and observations, including catalytic and substrate promiscuity of metabolic enzymes, alternative metabolic pathways [120-123], the probabilistic nature of metabolism [124,125], moonlighting enzymes [122,126,127], and others (for a review, see [8]).

To summarize, it appears that a great variety of chemical species in the cell can donate and accept electrons and thus perform as redox-active centers and/or components of electron relays. Notably, most, if not all, of the micronutrients and vitamins that are actively and continuously sought by living cells and organisms and assimilated from the environment represent sources of redox-active chemical species. Generally speaking, there are two major forms of electron transport that can be clearly distinguished in the cell. One resides in the bulk phase of the cytoplasm and is based on diffusible electron donors, acceptors, and redox-active electron shuttles. The other is mediated by the cytomatrix, a sponge-like skeleton made of metastable macromolecular complexes and sub-cellular structures. In reality, since the bulk phase continuously circulates through the sponge of the cytomatrix, while the cytomatrix itself is continuously remodeled in response to changing environmental and internal conditions, the cell as a whole represents a multiscale system of structured electron flow/circulation, where electrons move at blazing speed within the dense, structured media of macromolecules, relatively slow via diffusible electron carriers in the circulating bulk phase and at highly varied rates within steady-state, metastable macromolecular complexes. In other words, the two forms of electron transport discussed above should not be seen as reflecting a bimodal distribution of the characteristic lengths and times on which electron transport in the cell operates but, rather, as two opposite ends of a power-law distribution. It is also important to keep in mind that all biological systems continuously fluctuate and change in time and space in response to challenges and opportunities while developing, evolving, and adapting. This continuous change means that, under certain circumstances (when, for example, the cytomatrix is transiently destabilized and disorganized due to internal and/or external stresses), electron transport via diffusion in the liquid phase may transiently prevail over other forms of electron propagation, thus making the cell as a whole relatively less "conductive." On the other hand, when the internal and external organizational dynamics of energy/matter are in harmony (i.e., well matched and correlated), electron conduction through the cytomatrix may become the predominant form of electron transport, thus rendering the cell as a whole more "conductive." Overall, all else being equal, a cell with a relatively developed and well-organized cytomatrix is expected to be relatively more "conductive" but relatively less mobile, flexible, and adaptive, whereas a cell with a relatively disordered and stochastic cytomatrix is expected to be relatively less "conductive", but relatively more motile, flexible, and adaptive. Perhaps not coincidentally, the properties of the former are reminiscent of the properties of mature, differentiated cells, whereas the properties of the latter are reminiscent of the properties of young, differentiating cells, cancer cells, and stem cells.

Concluding this section, a note should be made of a distinct trend that can be discerned in the behavior of electrons within living matter: namely, their apparent tendency to move not only between redox centers along redox gradients but also from less defined and stable occupations/states to more defined and stable ones. Due to its dual wave-particle nature, a globally delocalized electron cannot be detected as an individual entity. As an electron is gradually localized, moving from less stable and undefined occupations/states to more stable ones, it becomes increasingly amenable to detection through such measurable manifestations as short- and long-lived radicals, redox centers of low and high oxidation potentials, and chemical bonds of varying strengths. Covalent bonds, for example, represent a popular and thus highly populated form of long-lived electronic states within living matter. It should be pointed out that radicals, redox centers, and covalent bonds are functional equivalents from the perspective of electron dynamics in the sense that they are simply different classes of the energy/matter arrangements allowing for electronic localization and the persistence of localized electronic states. It is also worth pointing out that the life expectancy of electrons within radicals, redox centers, and covalent bonds varies widely both within and between these classes of energy/matter arrangements. Radicals are associated with the shortest life expectancies of electron localization, and covalent bonds are associated with the longest, hence the tendency of electrons to move from radicals to redox centers and into covalent bonds.

Perhaps not coincidentally, the behavior of protons within living matter follows the same general pattern; i.e., protons tend to move from relatively unstable occupations/states to relatively stable ones, ultimately being captured and stabilized in bioorganic compounds and macromolecular structures in chemically bound forms. Therefore, biosynthesis can be viewed as the accumulation of energy/matter in a structured format, where electrons are captured and stored in long-lived states such as covalent bonds. Notice that chemical bonds play a dual role in biological organization and dynamics. They define a specific biological structure/dynamics (an identity), and at the same time, they define the pathways of electron flow within the biological structure. By releasing electrons from chemical bonds, catabolic reactions destroy both existing molecular structures and the existing patterns of electron flow they mediate, while liberating accumulated energy/matter for work and the creation of new structures and thus new patterns of energy/matter flow. Moving from relatively undefined and unstable occupations/states to increasingly defined and stable occupations/states, electrons pass through a living system/organization, animating, sustaining, and structuring it. Superficially, the process is reminiscent of an electrical discharge passing through and animating living matter. Some of the electrons released by catabolic reactions are recaptured via anabolic reactions, recreating the same structures or producing alternative structures and, correspondingly, the old or new patterns of energy/matter flow/circulation. Such continuous renewal and turnover ensure the maintenance, development, adaptation, and reproduction of useful structures and behaviors, while helping to eliminate obsolete, superfluous, irrelevant, and maladaptive structures and behaviors. Other electrons leave the living system/organization altogether and flow into external electron sinks, thus coupling the living system/organization to its environment. In addition, through the external work performed by a living system/organization on its environment, the flow of electrons passing through the living system/organization structures and animates the environment. In fact, electrons never stop moving, for even covalent bonds represent metastable states (flow/circulation patterns) that undergo spontaneous breakdown and disorganization. Structured macromolecular media such as proteins, DNA, lipids, and their complexes only accelerate, direct, and organize the interminable flow of electrons. As a result of molecular self-organization, driven and sustained by electron flow, various living structures, cells, organisms, and ecosystems continuously emerge, metamorphose, and transform one into another in an eternal process of transformation of forms, which unfolds simultaneously on multiple scales of space and time within the multiscale whole of the planetary life, held together and integrated by the invisible threads of moving electrons. As a consequence of such an arrangement, *molecules*, *cells, organisms, and ecosystems function as scale-specific constituents of one multiscale whole of energy/matter flow/circulation, where they represent both means and ends, at one and the same time.*

Notice that, since the Earth's biomass has been continuously increasing over macroevolutionary time at an accelerating pace [1], it appears that living matter as a whole grows by extracting and assimilating electrons and protons from nonliving matter at an accelerating rate. If we assume that the main difference between living matter and nonliving matter is that of organization, then life is a natural consequence of the evolution of nonliving matter. In other words, life is likely to be a rule rather than an exception in the Universe at large. This may mean that the living Earth grows and develops in an invisible competition with a great variety of alien life forms, which grow and develop throughout the Universe, feeding on the (same) flow of energy/matter that makes up the Universe.

### Pathways of electron flow and electronic coupling

The SOFT-NET interpretation posits that, in order to persist and grow in size and complexity, any living organization must be coupled to a source of and a sink for energy/matter. Moreover, to survive and prosper in conditions of continuous competition with other living organizations and life forms for energy/matter flow, any living organism/organization will strive to couple to its environment, both physical and social, in such a way as to maximize the rate of energy/matter flow through its internal organization, while minimizing the cost of maintaining and managing this flow. The one-dimensional model of the living organism shown in Figure 2 implies that the terminal point of an electron transport chain representing a living system/organization should always be coupled to a sink for electrons (an electron acceptor). If the sink is a chemical species, the reduced form of the corresponding species must be rapidly removed from contact with the terminal link (e.g., excreted) and replaced by its oxidized form in a cyclical manner. The rate of electron consumption by the sink (i.e., the rate of redox cycling in the sink) is a critical parameter, as it defines the overall rate of electron flow through the chain and, thus, the structure, behavior, and very existence of the chain. Note that the inherently flow-like nature of an electron transport chain allows for a great deal of flexibility and adaptation in response to both misfortune and opportunity. If a given sink becomes too slow, or too costly to be coupled to, or unavailable, a troubled electron transport chain may restructure and switch to an alternative sink that is more advantageous in terms of the rate of electron consumption and/or the cost of coupling. Likewise, an electron transport chain can explore both its environment and its internal organizational structure in a search for more profitable connections and forms of coupling. Even the terminal link of an electron transport chain may become a sink for electrons, provided that its oxidized form is rapidly delivered (e.g., produced, recycled, or imported) and its reduced form is promptly removed (e.g., consumed, recycled, or exported) from the chain. In fact, any link in the chain may potentially become a sink for electrons, thus allowing for a great deal of flexibility and complexity in the structure and dynamics of an evolving chain, including pathway branching and the formation of complex networks made of competing and cooperating electron transport pathways.

One of the well-known global sinks for electrons on Earth is oxygen. Aerobic organisms use oxygen as a terminal electron acceptor, actively circulating it through their internal organization and exporting reduced oxygen into the environment in such forms as CO_2 _and H_2_O. In the case of anaerobic respiration, on the other hand, a wide variety of substances are used as electron acceptors. It is worth pointing out that the term "anaerobic respiration" is an oxymoron because one is forced to say that bacteria "breathe" chemicals or "respire" minerals without even being in physical contact with them. Thus, it seems appropriate to replace the term "anaerobic respiration" with the concept of electronic coupling.

All microorganisms are coupled to their social and/or physical environments through the continuous export of electrons, performed either in an organized manner (via exchange) or unorganized manner (by dumping waste). Whereas many electron acceptors that are commonly used by microorganisms are soluble before and after reduction (e.g., oxygen, sulfate, nitrate, and carbon dioxide), the most abundant alternative electron acceptors in sedimentary environments are insoluble iron and manganese oxides in the form of Fe(III)- and Mn(IV)-bearing oxyhydroxide minerals. In fact, it has been suggested that an Fe(III)-reducing microorganism was the last common ancestor of extant life and that Fe(III) reduction was one of the earliest, if not the first, form of microbial "respiration" [128,129]. Metal-reducing bacteria employ a remarkable variety of strategies to couple their electron transport systems to insoluble minerals. Examples of coupling strategies include the following: i) shuttling electrons to mineral surfaces, by employing metabolites from terminal points of electron transport chains and/or diffusible redox couples as electron carriers [130,131]; ii) direct transfer of electrons to minerals from *c*-type cytochromes embedded in the bacterial outer membrane [132,133]; and, as recently discovered, iii) touching mineral surfaces with electrically conductive pili [134,135]. The use of electrically conductive pili for electron transport is not restricted to metal-reducing bacteria, as such pili have also been found in an oxygenic photosynthetic cyanobacterium and a fermentative thermophile, which produce pili when their primary electron acceptors (O_2 _or CO_2_) are in deficit [135]. Importantly, all of the strategies for electronic coupling between living cells and inorganic matter mentioned above are also employed for electron transfer, and thus electronic coupling, between microorganisms.

Both as a class and as individuals, microorganisms are extremely flexible and adaptive in terms of their electronic coupling to the environment. Whereas a wide variety of bacteria are able to reduce Fe(III) [136,137], a variety of metals, including Mn(IV), U(VI), Cr(VI), and Co(III), can be reduced by the same metal-reducer [138]. A bacterium may use electronically conductive pili to export its electrons to insoluble minerals, yet the same bacterium lacking a pilin gene can grow using fumarate or other metabolites as electron acceptors [134]. Alternatively, a bacterium may attach to a mineral surface and transfer electrons directly from its membrane-associated multiheme cytochromes. Alternatively, in the presence of metal chelators and/or diffusible electron shuttles, such as humics or quinone-based species, the same bacterium can grow without attaching to surfaces. One type of bacteria may use fumarate, Fe(III), elemental sulfur, or malate as preferred terminal electron acceptors, whereas another bacterial type may use nitrate, sulfate, or thiosulfate for the same purpose. In the absence of appropriate electron acceptors, electrons can be transferred to a partner microorganism [139]. Microbes may use oxygen or carbon dioxide as electron sinks, but when their preferred electron acceptors become depleted or temporarily unavailable, they switch to alternative sinks for electrons [135]. All of this evidence suggests that maintaining a fast and continuous outflow of electrons (i.e., an efficient electronic coupling) is more important than a concrete form of coupling. As all bacteria survive and grow by continuously competing and cooperating with one another in conditions of limited availability of energy/matter resources, the choice of a specific form of electronic coupling is likely defined by the interplay among the evolutionary history, habits, environmental niche, and continuously changing economic imperatives of the microbe in question. Indeed, when co-cultured with methanogens consuming hydrogen as electron donor, many fermentative bacteria switch their metabolism in favor of hydrogen production. It has been hypothesized that the energetic advantage of such a metabolic shift is mediated by hydrogenases that reduce protons with electrons derived from the electron-transport chains of fermenting bacteria, thus generating a high-demand product (hydrogen) that is rapidly consumed by the methanogens [136]. It is worth mentioning that, as a family, hydrogen-consuming methanogens do not really "care" where hydrogen comes from. It can be obtained from fermenting bacteria or from geothermal sources where hydrogen is produced from water reacting with basalt [140].

Microorganisms rarely, if ever, live as solitary individuals. Instead, through exchanges of various energy/matter forms with other organisms and nonliving matter, they are organized into microbial communities. On a larger spatiotemporal scale, these communities are organized into microbial ecosystems, which in turn, function as integral parts of the planetary-wide system of energy/matter transformation, exchange, and circulation known as global biogeochemical cycles. In methanogenic environments such as swamps, peat land, tundra, and the intestinal tracts of animals and insects, methanogenic archae team up with fermentative bacteria to digest complex organic matter. In a sequence of reactions, fermenting bacteria degrade polysaccharides, proteins, lipids, and other complex organics into lactate, ethanol, propionate, butyrate, and other simple organics. These products are degraded by proton-reducing acetogenic bacteria into methanogenic substances such as acetate and hydrogen, which are consumed by methanogens [136]. Because a secreted product (a sink for electrons) of one microbial species is a consumable (a source of electrons) for another species, electron transfer and, thus, electronic coupling among microorganisms occur in the form of the chemical substances they consume, excrete, and/or exchange. Thus, in essence, microbial communities and ecosystems act as electron transport chains and networks, much like metabolic enzymes inside the cell, but on a larger spatiotemporal scale. And like metabolic enzymes, microorganisms exchange electrons via two major routes: by means of diffusible chemical species and/or through direct contact, via macromolecular structures. Indeed, it was recently discovered that stratified microbial systems in marine sediments function as large-scale electron transport networks that connect spatially separated processes of oxygen reduction at the sediment surface to oxidation of hydrogen sulfide and organic carbon deep within the anoxic layers of the sediment. The organisms that have access to oxygen at the sediment surface perform the oxygen reduction for all organisms connected in the network, whereas the organisms that have access to electron donors within the sulfidic zone, deep in the sediment, perform the oxidation steps for all connected organisms within the microbial network [141,142]. It is believed that electrons flow from anoxic zones to oxygenated layers through a conductive network made of microorganisms and inorganic environmental elements, such as metal-containing minerals. Electron transport within such networks takes place in two major forms: through direct physical contacts, e.g., via electron-conducting pili and/or outer-membrane cytochromes, and by means of diffusible electron shuttles [142].

Remarkably, many exocellular chemical species that are used as electron donors, acceptors, and/or shuttles at the scale of microbial communities are *the same chemical species *that play *the same roles *at the scale of metabolic enzymes inside the cell. These chemical species can be conditionally divided into distinct, albeit overlapping, classes such as metabolites, inorganic substances, and redox-active centers. Examples of the metabolites that serve as electron donors and/or acceptors at the scale of microorganisms and at the scale of enzymes include lactate, fumarate, acetate, malate, and succinate. Examples of the inorganic substances used as electron donors and/or acceptors at both scales include water, hydrogen, protons, oxygen, sulfur, transition metals, carbon dioxide, sulfate, sulfide, nitrate, and nitrite. Examples of the redox-active centers operating at both scales as electron shuttles and/or electron relay elements include transition metals, riboflavin, melanin, and redox couples such as quinone-hydroquinone, cysteine-cystine, and sulfur-sulfide.

In addition to using the same chemical species for the same purposes, enzymes and microorganisms display conspicuously similar self-organizational dynamics. For example, even though microorganisms in microbial communities and metabolic enzymes inside the cell may function in solution as independent individuals, both tend to form aggregates, compartments, and networks at their respective scales, especially in conditions of limited availability of energy/matter resources. Such aggregates/compartments/networks mediate and, at the same time, are sustained by high rates of energy/matter flow passing through them. As an example, anaerobic bacteria and methanogenic archae form compact microbial granules that operate like an organ rather than a group of microorganisms functioning independently. Physical disruption of such granules results in severe impairment of methane production, indicating that the specific organization of these granules is required for maintaining high rates of metabolic fluxes in the microbial community [143]. As an example of self-organization at the subcellular scale, all six enzymes of the *de novo *purine biosynthetic pathway reversibly co-cluster in human cultured cells under purine-depleted conditions. It is thought that the enzymes form multiprotein complexes to ensure a high rate of *de novo *purine production to satisfy internal demand for purines in the absence of purine import, since the same enzymes remain disorganized in a purine-rich medium [144]. As another example, low CO_2 _levels induce the self-organization of bacterial carboxysomes, polyhedral organelles consisting of metabolic enzymes encased in a multiprotein shell. The carboxysome improves the efficiency of carbon fixation by concentrating carbon dioxide and directing it to ribulose bisphosphate carboxylase/oxygenase, which resides in the lumen of the organelle and catalyzes the CO_2 _fixation step of the Calvin cycle [145].

Importantly, self-organizational dynamics at the scale of microorganisms and at the scale of enzymes conform faithfully to the self-organizational dynamics expected of far-from-equilibrium systems. A fast flux of energy/matter through an open nonequilibrium system of energy/matter exchanges is accompanied by a high degree of organization and complexity, while low rates of energy/matter flux are associated with relatively disorganized systems. From symmetry considerations, it is not difficult to infer that electron flow acts as a keystone flux (a limited and limiting resource) that promotes self-organization at both scales.

According to the SOFT-NET interpretation, the recurrence of certain self-organizational patterns at different levels of the organizational hierarchy may mean that the recurring patterns are scale-invariant. Therefore, they can be used as conceptual guides or structural templates to infer organizational dynamics at all other levels of the organizational hierarchy. Indeed, it is not difficult to see, for example, that at the scale of multicellular organisms, the organ is an organizational replica of a microbial community. Akin to specialized microbial species comprising a microbial community, specialized cell types comprising an organ incessantly consume, secrete, and/or exchange electron donors, acceptors, and/or shuttles that circulate locally, within the organ/community, and systemically within a larger scale system/organization, (i.e., the body). Since a secreted product of one cell (or cell type) is a consumable for another cell (or cell type), the fluxes of circulating chemical substances integrate cells and coordinate their activities both within a given organ and within the organism as a whole. Driven by competition and cooperation between different cells and cell types, the flow of energy/matter within an organ (and an organism) is continuously organized and reorganized to the common benefit of all, as if by an "invisible hand", in a process of unsupervised economic optimization driven by the same principles and processes that shape and organize the market economy. Therefore, cells within a multicellular organism incessantly consume, produce, and exchange certain chemical substances not because they have been designed to do so but because their specific choices, habits, specializations, productive activities, and consumption patterns provide them with the continuous flow of energy/matter they require to live long and prosper, both as individuals (hence competition) and as an organ/organism/organization (hence cooperation). Indeed, as an example, consider a simplified outline of the metabolic relationships maintained among neurons, glia, and the vasculature in the brain. The neurotransmitter glutamate released by neurons at synaptic sites during neurotransmission is taken up into astrocytes, where it is metabolized, stimulating aerobic glycolysis and, as a consequence, the uptake of glucose from circulating blood. Lactate generated by astrocytes as a result of aerobic glycolysis is secreted and taken up by neurons to fuel neuronal metabolism. In addition, astrocytes produce glutamine, which is shuttled back to neurons, where it is converted to glutamate to regenerate the neurotransmitter pool. As astrocytic processes envelop both synapses and capillaries, multiple cell types as well as local and global circulation of energy/matter are physically, functionally, and metabolically integrated within the neuron-glia-vascular unit [146-148]. Of note, the organization and dynamics of the neuron-glia-vascular unit appear to be driven by continuous competition and cooperation among synapses, astrocytic processes, and different cells and cell types, whereas the incessant cycling and recycling of metabolites apparently serves to mediate electron transport between different cells and cell types.

From symmetry considerations, one can infer that electrons are transferred between individual cells within any organ via two major routes: by means of chemical substances circulating in the liquid phase (tissue microcirculation and central circulation) and via a putative, organ-wide, electron-conducting matrix made of dense macromolecular media. The latter may include the cytomatrices of the cells comprising the organ, the extracellular matrix, and cell-matrix and cell-cell contacts. Indeed, most organs are maintained as continuously remodeling sponges composed of cells and extracellular matrix elements, which are immersed in a liquid phase circulating locally and globally.

Symmetrically, at the scale of the whole organism, individual organs are integrated into one functional whole via circulating chemical substances, which are constantly produced, consumed, and/or exchanged by different organs. As an example, exercising skeletal muscles and other glycolytic tissues secrete lactate into central circulation, while the liver consumes lactate and generates glucose, which in turn is secreted back into central circulation for consumption by glycolytic tissues (the Cori cycle) [149]. As another example, upon prolonged oxygen deficit, a variety of mammalian tissues are thought to switch to fumarate "respiration." As a consequence, succinate, the end product of fumarate "respiration," is secreted into circulation and delivered to the lungs, where it is reoxidized to fumarate and malate, which in turn are recycled back to the fumarate-"respiring" tissues via central circulation [150-152]. From symmetry considerations, it is not difficult to infer that the circulating lactate, glucose, succinate, fumarate, and malate act as electron acceptors, donors, and shuttles that integrate metabolism within and across multiple organizational levels into one multiscale whole, the organism. Since electrons and protons constitute a cargo carried by all substances circulating within the organism, with covalent bonds and hydrogen atoms being major forms of transported electrons and protons, it is fair to suggest that the transport of electrons and protons is a key function of all circulating substances. At the same time, the multiplicity and chemical heterogeneity of circulating substances allows for the establishment of diverse and specific functional connections among a variety of differentiated/specialized enzymes, cell, tissues, and organs, all of which use one and the same circulation system for transport in the liquid phase. Indeed, in addition to electrons and protons, substances circulating within the organism in the liquid phase are made of (and thus mediate the transport, exchange, and circulation of) various chemical elements, including carbon, oxygen, nitrogen, sulfur, phosphorus, iron, and others. In fact, the organism as a whole can be seen as a system of interdependent and interconnected cycles of chemical elements, a system that is organized and functions not unlike the system of biogeochemical cycles of the planet and which, indeed, may simply be a scale-specific constituent thereof.

As discussed in greater detail elsewhere [8], and as implied in the image outlined above, central circulation, tissue microcirculation, and intracellular circulation are not separate and independent phenomena but scale-specific components of one and the same organism-wide system of liquid circulation. From symmetry considerations, one can infer the existence of an organism-wide electron-conducting matrix made of dense macromolecular media that spans, integrates, informs, and animates the whole of the organism. The existence of structured, organism-wide energy/matter flow/circulation via interconnected intra- and extracellular macromolecular structures would either immediately explain or significantly clarify a great variety of unexplained, mysterious, and/or gravely misunderstood phenomena, including consciousness, sleep, memory, anesthesia, pain, and the true mechanisms of action of many conventional and alternative medicines.

The principle of scale symmetry also allows one to predict that, when a preferred electron acceptor, such as oxygen, becomes suddenly unavailable or in deficit, a hypoxic cell will be forced to switch to and rely on alternative electron acceptors to maintain the high rate of electron flow required to sustain its internal organization and dynamics. Consequently, a hypoxic cell will unavoidably undergo organizational relaxation (disorganization) and restructuring to a smaller or larger extent, depending on its success in restoring and maintaining the energy/matter flux required to sustain its internal organization. Indeed, upon hypoxia, both prokaryotic and eukaryotic cells undergo metabolic rearrangements and reorganization of their electron transport pathways. As an example, by employing appropriate terminal oxido-reductases, the same microorganism (*E. coli*) can either adapt to a lower oxygen tension or recouple to qualitatively different electron acceptors, such as nitrogen oxides or fumarate [133]. As another example, by employing appropriate sets of proteins, sulfur-dependent archaebacteria can grow equally well in anaerobic and aerobic conditions, either by reducing elemental sulfur to H_2_S (in the absence of oxygen) or by oxidizing elemental sulfur to H_2_SO_4 _(in the presence of oxygen) [153]. In higher organisms, HIF-1α, a transcription factor and oxygen sensor, triggers a metabolic rearrangement that replaces respiration with glycolysis and, consequently, CO_2 _generation with lactate production [154,155]. In conditions of prolonged oxygen deficit, cardiomyocytes, kidney cells, and certain other cell types can switch to fumarate "respiration," as such a metabolic rearrangement allows them to anaerobically maintain electron flux, mitochondrial membrane polarization, and ATP synthesis [150-152].

In reality, at any given moment, any given cell is most likely relying simultaneously on several (competing and cooperating) alternative sources of and sinks for electrons, with the total electron flow being distributed highly unevenly between alternative sources, sinks, and pathways connecting them. It is fair to suggest that, as the environment and/or the internal state of a cell changes, the cell (whether animal or microbial) reorganizes its preferences in terms of sources, sinks, and pathways of electron flow, in accordance with changing economic realities and imperatives, by making choices, learning, forming memories, and engaging in habitual behaviors. Cellular choices are manifested as changes in gene expression profiles, metabolic rearrangements, and restructuring of the cytomatrix. Cellular memory is manifested as preferred (i.e., habitual and thus economically least costly) gene expression profiles, metabolic states, and spatiotemporal organization of the cytomatrix. Oxygen is preferred as a terminal electron acceptor for several reasons, one of which is its abundance and accessibility in most surface environments. It is reasonable to suggest that the emergence of complex life forms on the planet coincided with the emergence of oxygenated atmosphere mainly because oxygen acts as a vast, deep, and readily accessible electron sink that can support much higher rates of electron flow passing through biological structures-processes compared to alternative electron sinks, and thus, it can support much higher degrees of biological diversity, complexity, and order. Since ontogeny recapitulates phylogeny, it is hardly coincidental that oxygen has been found to function as a key regulator of ontogeny [154]. Before the circulatory system is established, mammalian development takes place in a relative hypoxic environment. The increasing availability and consumption of oxygen correlates with the progress in differentiation, accompanied by increasing complexity and order within the developing embryo, whereas hypoxia promotes de-differentiation and the undifferentiated state of stem cell and precursor cell populations (see [154] and references therein).

The image outlined above greatly simplifies the understanding of many biological phenomena. One can infer, for example, that upon hypoxia, affected cells in animal tissues start secreting lactate, vascular endothelial growth factor (VEGF), and other metabolites, growth factors and cytokines, simply because they use secreted molecules as electron acceptors that proved to be useful in the past. Of note, VEGF and related growth factors fold into an unusual cyclic structure called a "cystine knot," which may have nontrivial electronic properties [156]. By the same token, vascular endothelial cells are stimulated to proliferate and to move toward the source of VEGF and other molecules secreted by hypoxic cells because they use secreted VEGF, metabolites, and other molecular species directly or indirectly as electron donors and/or acceptors. Cellular dynamics within a tissue subjected to hypoxia can be thus seen as a relaxation and reorganization of a metastable electron transport network made up of differentiated/specialized cells engaged in market-type exchanges of energy/matter forms, where individual cells continuously struggle to survive and succeed, seeking economic advantages and exploring opportunities. Note that, according to the principle of scale symmetry, a similar process simultaneously takes place inside each hypoxic cell, at the subcellular scale. Indeed, being a structured electron transport network, the cytomatrix is necessarily forced into relaxation and reorganization upon hypoxia, which cuts off the internal organization of the cell from its primary terminal electron acceptor and thus undermines established pathways and configurations of electron transport. The specialized enzymes and proteins making up the cytomatrix and electron transport pathways will necessarily disorganize, dissociate, and restructure upon exposure to conditions of hypoxia because the rate of electron flow through their internal structures and the multiprotein complexes they comprise becomes too low to sustain the pre-existing level of order. In other words, hypoxia forces the reorganization of preferences in terms of sources, sinks, and pathways of electron flow on multiple organizational levels simultaneously. Consistent with this scenario, glycolytic enzymes in plant cells have been shown to reversibly partition from a soluble pool to a mitochondria-bound pool upon increased respiration and back into the soluble pool upon inhibition of respiration. Mitochondrially-associated enzymes form a functional glycolytic sequence, presumably in the form of a multiprotein complex, that supports mitochondrial respiration through substrate channeling, as revealed by NMR spectroscopy tracing of ^13^C-labeled precursors [157]. Notably, the increased demand for pyruvate consumption by respiring mitochondria is met through reversible partitioning and compartmentalization of glycolytic enzymes, rather than through the changes in their abundance [157]. In this context, it is worth pointing out that lactate and pyruvate constitute a major redox pair operating at multiple organizational levels in living cells and organisms. The reduction of pyruvate to lactate occurs under anaerobic conditions and involves the transfer of electrons from NADH to pyruvate, regenerating NAD^+^, which is then used in glycolysis as an electron acceptor. Consequently, pyruvate acts as a major electron sink in the glycolytic cells and tissues that secrete lactate into environment. At the same time, those cells and tissues that have easy access to activated oxygen can use lactate as an electron donor, converting it to pyruvate, which is then consumed by respiring mitochondria. It goes without saying that dumping electrons into pyruvate and exporting resulting lactate is a popular but not the only strategy for coping with hypoxia. For example, reactive oxygen species, and decomposing peroxides and endoperoxides constitute usable and abundant sources of activated oxygen, i.e., electron sinks (see, for example, [158]). Fittingly, the binding of a variety of growth factors and cytokines to their cognate receptors leads to the production of hydrogen peroxide and other reactive oxygen species by ligand-receptor complexes [159], presumably through the water oxidation pathway [160,161]. In addition, a variety of enzymes, such as NAD(P)H oxidases and cyclooxygenases, actively generate reactive oxygen species and various peroxides and endoperoxides both inside and outside living cells. Because activated oxygen is a familiar and attractive electron sink, the choice as to whether to use the activated oxygen delivered systemically by hemoglobin or that produced locally from ROS, peroxides, and/or endoperoxides is simply a "business decision" from the perspective of any given cell. Obviously, the same logic applies to the subcellular scale, where enzymes, multiprotein complexes, and macromolecular structures use reactive oxygen species, peroxides, and endoperoxides as acceptors, donors, and shuttles of electrons. Notice that a "business decision" at one level of organizational hierarchy (e.g., at the scale of the cell as a whole) is simply a consequence and a manifestation of decisions and self-organizational dynamics taking place on underlying levels of organizational hierarchy (e.g., at the molecular and subcellular scales). Importantly, hydrogen peroxide, endoperoxides, and many other metastable reactive species can function as both acceptors and donors of electrons, which can be either exported into or imported from environment, depending on the need. Such an interpretation may help to explain a great variety of otherwise surprising experimental observations and findings, including a key role of hydrogen peroxide in a great variety of seemingly disparate phenomena, such as lymphocyte activation [162], chemotaxis [163], paracrine signaling [164], innate immunity [165,166], angiogenesis [167], the activation and signaling of cell surface receptors, transcription, proliferation, metabolism, and signal transduction [168].

The continuous deposition, digestion, and remodeling of the extracellular matrix (including endo- and exoskeletons) by cells can be interpreted in the same terms. Namely, the cells secreting extracellular matrix elements use secreted (macro)molecules as electron acceptors, while the cells that digest extracellular matrix elements use them as electron donors. Moreover, a properly deposited and organized extracellular matrix may also function as a structured electron-conducting medium. The great abundance of disulfides in extracellular matrix elements is traditionally interpreted as a means to stabilize secreted macromolecules. However, it can also be interpreted as a means to endow secreted molecules with specific electron-conducting properties and organization. Fittingly, scanning electrochemical microscopy imaging of single cells shows that the oxygen respiration profiles of cells do not mimic the cell topography, as one would expect. Instead, oxygen is preferentially depleted at the points where the cell makes contacts with the substratum [169]. These observations suggest that, although oxygen can reach terminal points of electron transport chains inside respiring mitochondria by free diffusion, it may not need to do so in the context of organized cells and tissues, provided electrons can flow unimpeded through the cytomatrix and cell adhesion complexes to intermediate electron sinks, such as the extracellular matrix and/or neighboring cells, and through the latter, to terminal electron sink(s) (e.g., activated oxygen) situated elsewhere. In other words, within the organized environment of a mature healthy tissue, the well-coupled and appropriately structured (i.e., normal, healthy, and differentiated) cells may find it economically more efficient to reduce oxygen at great distances from the terminal points of their mitochondrial electron transport chains. Similar to microorganisms within stratified microbial communities in marine sediments [141,142], animal cells within tissues can potentially use their own cytomatrix, extracellular matrix, and the cytomatrices of cooperating neighbors as structured macromolecular media for rapid and efficient electron transport. Of note, such a process would support tissue organization and, at the same time, be supported by it. In other words, the tissue disorganization identified by a pathologist in a tissue biopsy may in fact signify a disorganization of electron transport networks and, thus, be a sign of impaired flow/circulation of energy/matter within and through an affected tissue. Pertinently, rapid tissue/organism-scale electron transport via macromolecular structures would immediately explain a great variety of paradoxes and puzzles, including the longstanding mystery of oxygen delivery to cytochrome *c *oxidase in high workload states [170] and the synchronization and coordination of cellular activities at the scales of tissues and whole organisms, among many others. The suggested image does not exclude or contradict the textbook scenario of ground-state oxygen reaching cytochrome *c *oxidase by free diffusion and being activated inside mitochondria. However, since the textbook scenario requires neither a structured cytomatrix nor structured tissue, which are routinely destroyed upon the isolation of mitochondria for biochemical analysis, it is the only mechanism of "respiration" (electronic coupling, in fact) that can be discovered by the reductionist biochemistry operating within the interpretational framework of equilibrium thermodynamics.

For the same reason, we have only a rudimentary understanding of the true biological role(s) and relevant physicochemical properties of the extracellular matrix/environment within the organism. Yet it is obvious that the extracellular matrix/environment is a key factor defining cellular structure and behavior, which continuously co-evolves with cells in all phases of the organismal life cycle, from conception through development and maturation to aging and death. Cells continuously shape their environments, adapting environments to their ends, while environments continuously shape cells adapting to them. Among other ramifications, this interplay suggests that one of the key mechanisms facilitating the self-organization of cells within developing and/or adapting tissues and organisms is a process called *stigmergy*. "Stigmergy is a mechanism of spontaneous, indirect coordination between agents or actions, where the trace left in the environment by an action stimulates the performance of a subsequent action, by the same or a different agent. Stigmergy is a form of self-organization. It produces complex, apparently intelligent structures, without need for any planning, control, or even communication between the agents." [171]. First discovered in studies on social insects, such as ants and bees, stigmergy, i.e., spontaneous coordination via environmental imprinting, appears to be a scale-invariant phenomenon. Certainly, humans (their preferences, values, and habitual thought and behavior patterns) are incessantly and often ruthlessly shaped by the institutional/cultural environments within which they are born, develop, and live, even though institutions are created and shaped to serve human ends. Herein, incidentally, resides one of the main sources of the interminable conflict between the young and the old. Existing institutions, and thus the ideas and conventions they represent, invariably reflect the interests of the old, who brought them forth in the past and benefit from them in the present, while the maturing youth always face the choice between meek submission, which reproduces, reinforces, and, thus, is rewarded by the old, and revolt, i.e., the active efforts of breaking free from conventions, destroying the old, and bringing forth the new, to continue with life and development and avoid aging, degeneration, and death.

Returning to the main topic of our discussion, potentially all chemical substances secreted and/or consumed by cells within the organism are or can be used as electron acceptors, donors, and/or electron transport media. Since cell differentiation/specialization normally entails the creation and maintenance of a high degree of spatiotemporal order, which requires a stable and rapid flow-through of energy/matter, complex differentiated cells are expected to exhibit relatively well-defined and, thus, relatively inflexible preferences in terms of electron donors, acceptors, and the pathways of electron flow and to inhabit relatively stable, well-structured, and protected environments with rapid and efficient circulation. This may explain, for example, the disproportionately high rate of oxygen consumption by the brain, as compared to other tissues, and the high sensitivity of neurons to ischemia and/or circulation disturbances. On the other hand, undifferentiated and dedifferentiated cells, such as stem cells and cancer cells, which, correspondingly, are not yet and no longer burdened by a high degree of internal order, are expected to have relatively undefined and flexible preferences in terms of electron donors, acceptors, shuttles, and the pathways of electron flow. Such properties make these cells highly adaptive and flexible, giving them competitive advantages in hypoxic niches with impaired circulation. This may explains why cancer, neurodegenerative disorders, and cardiovascular disease have reached epidemic proportions in industrialized countries, where sedentary lives (causing hypoxia and slow circulation) are spent in chemically polluted environments (causing chemical hypoxia and further impairment of circulation) and why vascular (i.e., circulation) disorders are implicated as causative conditions in a variety of pathologies, ranging from chest pain and heart attacks to strokes and neurodegeneration. In fact, most likely, the roots of *all diseases *can be traced either to prolonged impairment of the circulation/flow of fluids or to prolonged impairment of energy/matter flow/circulation via dense macromolecular structures, with the two forms of energy/matter flow/circulation being inherently interdependent and co-defining. One cannot help noticing the uncanny parallels between the outlined image and the traditional Eastern notion that all diseases are caused by disturbances and imbalances in the flow of energy that sustains all living beings (e.g., see the Chinese concept of *Qi *[172]).

Generalizing, the cells that succeed in becoming well coupled to their physical and social environments in terms of the inflow and outflow of electrons (and thus other forms of energy/matter) will tend to increase in size and/or number and to become relatively more complex, specialized/organized, long-lived, and efficient in terms of energy/matter propagation, transformation, and exchange. However, such cells will tend to become relatively less mobile and less flexible/adaptive, as compared to weakly coupled cells (not unlike human individuals and their organizations). Any intervention or stress that significantly impairs electron coupling and/or electron transport within and/or between cells (such as hypoxia, starvation, loss of cell-to-cell or cell-to-matrix contacts, impairment of circulation due to mechanical, heat, chemical, or oxidative stresses, deleterious mutations, environmental toxins, and other internal and external stressors) will lead to disorganization, restructuring, and adaptation of the affected subcellular structures, cells, and tissues, manifested as a relative loss of complexity, dedifferentiation, structural relaxation, increased motility, displasia, and metaplasia. When self-contained in space and time, due to ensuing self-organization of the same or alternative structures, these changes represent a normal physiological response to stress (i.e., adaptation through relaxation, turnover, and reorganization [6]), which is often beneficial, allowing for learning, innovation, and advances in structure and dynamics, hence the hormesis effect [173] and the adage "what does not kill you makes you stronger." A runaway response, either due to chronic impairment of circulation or severe acute stress, will lead to irreversible pathological changes, degeneration, and death. Chronic impairment of circulation is much more insidious than acute stress because degradation is gradual and often imperceptible until it is too late for intervention.

Of note, the aforementioned generalizations imply that all stressed cells should display common changes in certain physical characteristics, which are largely independent of cell type, the nature of stress, and the scale of response. Indeed, universal and scale-independent changes in such general physical characteristics as turbidity, viscosity, and membrane potential were observed in a variety of living cells reacting to a variety of stresses and studied by early cytologists in the 1950s and 1960s before the rise and ensuing totalitarianism of molecular genetics [174,175].

To conclude this section, any molecular structure can potentially serve as a sink for and/or as a source of electrons. When a molecular structure is synthesized and electrons are captured in the covalent bonds defining the structure, the structure serves as a sink for electrons and, at the same time, as a structure-process mediating the capture, transformation, transport, and/or storage of energy/matter. The synthesized structures can be exported, or retained and stored for future use, and/or used as a means for the procurement, transformation, and/or transport of energy/matter. A molecular structure serves as a source of electrons when it is catabolized. When a molecular structure is catabolized within an organized context, most of the released electrons are routed along organized pathways of electron flow and used for animation, work, and (re)creation of structures and behaviors, both old and new. When a molecular structure-process is catabolized within a disorganized context, most of the released energy is dissipated, often promoting further disorganization. Biosynthesis of molecular structures represents an accumulation of energy/matter in long-lived states in a structured format. Catabolism of molecular structures represents an organized release of accumulated energy/matter.

Because the release of electrons from such stable states as covalent bonds requires activation energy and because all nonequilibrium systems, by definition, always dissipate, all living organizations require a continuous input of excitation energy in order to keep "the fire within" alive.

### On the role of water

Electron flow mediated by redox reactions and transient radical intermediates implies the initiating events of charge separation that generate unpaired electrons and holes. The planet-wide expansion of living matter in size and complexity over macroevolutionary timescales also implies the existence of an inexhaustible source of such initiating events. Moreover, the flow nature of living organizations implies that the *rate *of *de novo *free radical generation is a critical parameter, for it defines the rate of electron flow through the living organism and, thus, its ability to persist, grow, and prosper. Since life is invariably associated with water, water is a prime suspect for the source of free radicals generated *de novo*. Indeed, consider the following examples of splitting and recombining water molecules [176]:

(7) H• + •OH ↔ H_2_O

(8) HO• + •OH ↔ H_2_O_2_

(9) H• + •H ↔ H_2_

(10) H• + O_2 _↔ HOO•

(11) HOO• + HOO• ↔ H_2_O_2 _+ O_2_

At thermodynamic equilibrium in standard conditions, H_2_O is by far the most stable molecular arrangement, and thus, it will greatly predominate over other species. Other possible arrangements, including highly reactive radicals, do arise spontaneously due to energy fluctuations and molecular recombination. However, in the absence of external energy input, their average lifetimes are short, and the frequencies of their spontaneous emergence are low. The unstable reactive species arising spontaneously can be thought of as packets of transiently captured energy/matter that require little or no activation to use. In principle, being adaptive nonequilibrium systems that feed on energy/matter, cells/organisms can survive and prosper by harvesting rare events, moving to and populating those environments where such events are relatively frequent, and/or co-structuring themselves and their environments in such a manner as to increase the consumption of energy/matter in the form of metastable reactive species. Water-born radicals can be passively harvested or actively procured. Although passive harvesting requires less organization and order, and thus is less costly, the life of a passive harvester depends on uncontrolled and often unpredictable external conditions. Sooner or later, the competition between passive harvesters sharing the same environmental niche would necessarily lead to the emergence of structures and processes allowing for the active procurement of water-born radicals. One of the obvious ways to accelerate the harvesting of water-born radicals is to develop structures/systems that allow for swimming through water and/or for pumping water through internal organization. The rate of swimming and/or pumping will correlate with the rate of energy/matter harvesting and, thus, will be self-regulated by the balance between the cost of swimming or pumping and the benefits of active energy/matter procurement. Such an interpretation may explain the all-pervasiveness and all-importance of self-regulated mechanisms enabling movement and water circulation on all levels of the biological organizational hierarchy. In addition, since both passive and active harvesting require the creation and maintenance of economically efficient systems mediating the capture, transport, storage, and exchange of energy/matter within living cells and organisms, the aforementioned interpretation may also explain the universal, sponge-like, fractal organization pervading all levels of the biological organizational hierarchy, from organisms and tissues down to cells, genomic DNA, and proteins. As suggested by West, Brown, and Enquist, the physical (living) systems/structures that mediate the transport, distribution, and exchange of energy/matter within living cells and organisms are forced to adopt fractal geometry by the evolutionary pressure for metabolic efficiency, because fractal geometry is an economically optimal solution that maximizes the area of exchange while minimizing the costs of transport through and maintenance of a fractal distribution system that fills and services a three-dimensional volume [8,60,62]. The image outlined above is also consistent with the hypothesis put forward earlier in our discussion that genomic DNA may function primarily as a macromolecular structure/sponge mediating the harvesting, transport, storage, and distribution of electrons, electron holes, and other basic energy/matter forms. Of note, such a hypothesis immediately explains i) why most of genomic DNA in most organisms is noncoding, ii) why there is no correlation between genome size and the complexity of an organism, and iii) why the largest genomes are found in some of the most primitive organisms that live in liquid water exposed to solar radiation, such as protists and algae [177]. But can water be a significant source of activation energy?

The splitting and recombination of water molecules can be experimentally evaluated by analyzing the *de novo *formation of hydrogen peroxide (H_2_O_2_), one of the relatively stable products of water recombination. Studies on water splitting show that virtually any form of energy supplied to water leads to the production of reactive oxygen species, albeit with relatively low efficiencies, thus making ROS a readily available, yet limited and limiting resource. Examples of water treatments that result in water splitting include (but are not limited to) electrolysis, sonolysis, photolysis, thermolysis, chemolysis, and mechanolysis as well as freeze-thawing and evaporation-condensation cycles (see [176] and references therein). The presence of ions and oxygen in water significantly augments the efficiency of water splitting, as does the presence of transition metals [176]. It was recently pointed out, for example, that sterile culture media left on the bench under normal room lighting can be an unrecognized source of oxidative stress in lab experiments, as photochemical mechanisms can generate 1-20 μM H_2_O_2 _in such media [178].

From a larger scale perspective, experiments on water dissociation performed in the 1950s and 1960s show that electrical discharge through water vapor yields large amounts of hydrogen peroxide and molecular oxygen [179]. Consequently, a hypothesis of the abiotic origin of oxygen via water splitting and hydrogen outgassing was once entertained [180]. In fact, it is currently believed that the early Earth may have lost over a third of its oceans due to photolysis of water vapor and outgassing of hydrogen, while Venus may have lost all its water in this manner [181,182].

Earlier studies on water splitting and recombination have been recently buttressed by the dramatic discovery of the so-called water oxidation pathway. Briefly, it has been found that antibodies, regardless of their source or specificity, catalyze the production of H_2_O_2 _from water and singlet oxygen (^1^O_2_*) [160,161]. The source of activated oxygen seems to be unimportant. Oxygen can be activated chemically (e.g., by thermal decomposition of endoperoxides) or photochemically (e.g., by light irradiation in the presence of oxygen photosensitizers such as hematoporphyrins or riboflavin) [160,183,184]. Isotopic labeling experiments and quantum chemical methods suggest that water is oxidized by ^1^O_2_* to hydrogen peroxide through the formation of hydrogen polyoxides, such as HOOOH and HOOOOH, and their radicals, HOOO• and HOOOO•, as intermediates [161,185]. Polyoxides are compounds of the general formula RO_n_R, where R is hydrogen or other atoms or groups, and n is greater than or equal to 3. Polyoxides are believed to be key intermediates in atmospheric chemistry and the chemistry of combustion and flames [186]. Thus, hydrogen polyoxides and their radicals appear to mediate oxidation (combustion) processes spanning atmospheric, environmental, and biological systems. More recently, it was found that four amino acids (tryptophan, methionine, cysteine, and histidine), by themselves, are able to catalyze the production of H_2_O_2 _and an oxidant with the chemical signature of ozone, presumably via the water oxidation pathway [187]. In the latter experiments, UV irradiation and 6-formylpterin were used for the activation of oxygen. Since catalysts do not invent chemical transformations but only accelerate them, the diverse and facile reactions of water splitting, recombination, oxidation, and reduction, accompanied by the production of atomic and molecular oxygen and hydrogen, hydrogen peroxide (HOOH), hydroperoxyl (HOO•) and hydroxyl (•OH) radicals, the superoxide anion (O_2_•^-^), singlet oxygen (^1^O_2_*), ozone (O_3_), the ozonide radical anion (O_3_•^-^), dihydrogen trioxide (HOOOH) and its radical (HOOO•), and other reactive oxygen species, are inherent propensities of water, which can be readily augmented by energy input and simple and ubiquitous organic and inorganic catalysts.

It should be pointed out that, in addition to the propensity of bulk water to capture energy in the form of relatively long-lived reactive species, which then can be transported and distributed via passive diffusion and/or active circulation in the liquid phase, ordered molecules of interfacial water lining macromolecular surfaces, act as a polymer-like medium that enables and mediates the capture and long-range transport of such energy/matter forms as electronic and vibrational excitations and electrons and protons [67,176,188-190]. Because reorientation of water molecules in the bulk phase requires concerted hydrogen bond rearrangement [191,192], the presence of an interface *per se *induces the relative ordering of interfacial water, whereas specific order and its extent appear to be defined by the chemical composition and structure of a given interface [193,194]. Moreover, it is reasonable to assume that, in nonequilibrium conditions, an even more specific structure(s) of interfacial water will be selected from available competing alternatives and stabilized by the flow of energy/matter it supports and, at the same time, is supported by. In other words, interfacial water behaves essentially as a structurally adaptive, animate medium. Due to the exceptionally high densities of macromolecules in the cell (300-400 mg/ml of proteins and RNA alone [86]), a large fraction, if not most, of cellular water is expected to be interfacial. Therefore, water can mediate the transport of electrons, protons, and other basic energy/matter forms inside the cell in two different ways: by means of diffusible species in the bulk phase and via structured water pathways on macromolecular surfaces. Since, as discussed earlier, the bulk phase continuously circulates through the sponge of the cytomatrix, while the cytomatrix itself is continuously remodeled on multiple timescales, water dynamics inside the cell will necessarily take place on multiple timescales simultaneously. Assuming, from symmetry considerations, a power-law distribution of characteristic timescales in the molecular dynamics of the cytomatrix, approximately 80% of interfacial water will exhibit relatively fast dynamics, i.e., will be perceived as having a bulk-like character, while only about 20% of interfacial water will appear as relatively slow, i.e., structured. Since most of the experimental techniques used to study molecular dynamics measure averages, averaging a power-law distribution will give a misleading impression that most cellular water is free, since the measured (averaged) mobility of water in the cell will be only a few times slower than its mobility in the bulk phase. In reality, although most of water in the cell is dynamic, it is not exactly "free" because the dynamics of cellular water is, to a large degree, a reflection of the (animated) dynamics of the cytomatrix, with both dynamics being mutually co-defining. In a conceptually analogous way, at the human scale, employees moving around during working hours appear to be "free," when in fact most of them are not (most of the time). Here, too, the dynamics of employees define the dynamics of their organization, while at the same time, the dynamics of the organization define the dynamics of its employees. Such an interpretation of cellular water is consistent with and reconciles a variety of reported estimates of water mobility within the cell [86,195], and thus it may help to resolve the age-old controversy over structured water in biology. The outlined image of cellular water dynamics also allows one to explain why relatively less structured cells (e.g., cancerous, dedifferentiated, and dividing cells) exhibit increased mobility of their internal water [196,197] and why the differences in structured water between cancerous and normal cell populations are difficult to exploit. Notice that, once again, the SOFT-NET interpretation suggests that what are perceived as competing alternatives within the conceptual framework of classical physics are, in fact, cooperating complements when empirical data are reinterpreted using a more adequate conceptual framework.

Concluding this section, the remarkable proficiency of water in capturing virtually any form of energy and transforming it into reactive oxygen species of various lifetimes and reactivities makes water an inexhaustible and versatile source of activation energy. However, due to the high thermodynamic stability of the water molecule, water-derived activation energy is always a limited and limiting resource. It is reasonable to hypothesize that water, by capturing external energy, converting it to highly reactive radicals, and activating a variety of chemical species in contact with water, may have enabled and facilitated versatile combinatorial chemistry and spontaneous synthesis, degradation, and recombination of organic and inorganic molecules on primeval Earth. Because spontaneous synthesis, degradation, and recombination of molecules proceeded in conditions of limited supplies of activation energy, catalysts, and building material, geochemical evolution in appropriate environments may have become possible. It is fair to suggest that those molecular species that were least costly in terms of their own synthesis and maintenance and that, at the same time, excelled at enhancing such properties of water as the capture, transformation, transport, exchange, and ordering of usable energy/matter forms would multiply, persist, and prosper. Such a positive-feedback process of cooperation with water in consuming, transforming, structuring, transporting, and accumulating energy/matter would allow for the emergence of efficient, self-promoting, and self-sustaining cycles of energy/matter capture and chemical synthesis in primordial conditions, thus driving geochemical evolution accompanied by the accelerating consumption of energy/matter from the environment. In this regard, it should be pointed out that the productive activities of all living organisms and organizations, whether extant or extinct, can be boiled down to the consumption, transformation, ordering, transport, accumulation, and exchange of energy/matter forms, with the intent of persisting in time and expanding in space by means of creating the increasingly advanced structures and processes that facilitate the consumption, transformation, ordering, transport, accumulation, and exchange of energy/matter forms, and so forth, with the ultimate purpose being the perpetuation and expansion of living matter as a whole.

### On the origin of life

Addressing the question of the origin of life, it is useful to begin with the role of water in the formation of stars and planetary systems. Upon star formation, following the initial gravitational collapse of dense molecular clouds, with strong shock waves rapidly heating up the circumstellar envelope of a newborn star, all of the available atomic oxygen is promptly transformed into water, making gaseous water the most abundant species after molecular hydrogen in star-forming regions [198]. In the course of protostellar evolution and the formation of a circumstellar disk, a predecessor of solid planetary bodies, newly formed water is increasingly subjected to high-energy stellar radiation, which promotes reverse reactions of water photodissociation into atomic oxygen, OH, and hydrogen [198]. However, recent theoretical studies suggest that, similar to the ozone layer shielding the Earth's surface from solar UV radiation, water created *in situ *at the surface of the circumstellar disk will protect any water vapor produced via gas-phase reactions and evaporation of icy planetesimals, thus leading to water self-shielding and a runaway feedback process promoting the rapid accumulation of water and OH in the protoplanetary disks. Moreover, water will protect any molecular species generated by gas-phase chemistry, allowing for a rich organic chemistry to persist even as the dust grains evolve towards planets [199]. In addition, water molecules themselves can act as molecular catalysts in radical-mediated gas-phase reactions, facilitating and accelerating molecular recombination processes [200]. Laboratory experiments and modeling of photoexcited water suggest that as much as 50% to 70% of the photon energy will be deposited locally in the disk atmosphere as heat, with the products of photodissociated water (OH, O, and H) being the primary heating agents [199,201,202]. In other words, it appears that the primordial organic "soup" is "cooked" in the gas phase whenever and wherever planets form, being a natural consequence of the evolution of circumstellar disks. Indeed, gas-phase reactions, solid-state chemistry, and gas-grain interactions involving interstellar material can produce a variety of complex organic molecules [203]. Emission spectra from regions of planet formation confirm a high abundance of simple organic precursors such as HCN, C_2_H_2_, CO_2_, water vapor, and OH throughout young circumstellar disks, indicating that the protoplanetary disk indeed supports active organic chemistry [204]. Laboratory experiments imitating conditions in star-forming region show that water-assisted photochemistry can generate a bewildering diversity of organic molecules from simple starting mixtures, including self-assembling amphiphilic molecules and photoactive organics [205]. The chemical composition of comets and meteorites, which is thought to reflect the physicochemical processes operating before and during the birth and evolution of the solar system, is also consistent with the above-mentioned scenario [206,207].

Notice that, in essence, the water formed in star-forming regions represents energy/matter captured in a long-lived form in a structured format. Once formed, gaseous water, together with the mineral solids of the protoplanetary disk, acts as a "condensed" medium that continues capturing the energy/matter comprising and flowing through the Universe by enabling and facilitating gas-phase and solid-state organic synthesis (i.e., the consumption, transformation, ordering, transport, and accumulation of energy/matter). Energy/matter is captured by water vapor within the evolving protoplanetary disk in many different forms, including gravitational energy, heat, radiation, and a variety of chemical species, and is transformed into different forms, notably, into locally deposited heat, secondary photon emission, reactive radicals, and diversifying and accumulating organic and inorganic materials. In other words, water vapor acts as a protective, enabling, and transforming medium that absorbs and retains energy/matter from the external environment and channels it to stimulate molecular recombination and active combinatorial chemistry, thus promoting the accumulation, diversification, and evolution of organic materials within the protoplanetary disk. It is reasonable to suggest that various chemistries and diverse molecular species populating the protective milieu of the evolving protoplanetary disk will compete and cooperate for the limited resources of usable energy, reactants, and catalysts available within the disk. Those chemical species and reactions that are least costly in terms of their reproduction and maintenance and that, at the same time, excel at enhancing such properties of water vapor as the consumption, transformation, structuring, transport, and accumulation of usable energy/matter forms will persist, expand, and prosper.

Such a scenario is consistent with the composition of organic material found in carbonaceous chondrites, one of the most primitive meteorite classes, the formation of which is thought to coincide or even predate the emergence of the solar system. As exemplified by the well-studied Murchison meteorite, the organic material found in carbonaceous chondrites can be roughly divided into two categories, a minor one consisting of soluble "free" molecules, with general composition grossly similar to terrestrial petroleum, and a major insoluble part made of a highly cross-linked network of complex macromolecular material comprising diverse polycyclic aromatic hydrocarbons [208-211]. Notably, many classes of compounds found in carbonaceous chondrites are present in living organisms. These include, but are not limited to, amino acids, carboxylic acids, hydroxyacids, sugar-related compounds, amines, amides, phosphorus-containing compounds, nitrogen heterocycles (purines, pyrimidines, quinolines/isoquinolines, and pyridines), sulfur heterocycles, aromatic and aliphatic hydrocarbons, porphyrins, and terpenoids [209]. However, unlike their organismal counterparts, organic compounds found in meteorites tend to exhibit a broad structural diversity within their corresponding classes (74 different amino acids were found in the Murchison meteorite alone) and a rapid decline in abundance with increasing molecular size. Such patterns suggest relatively random combinatorial synthesis and degradation involving small free-radical initiators and intermediates [208,210]. At the same time, apparent biases, such as a small but significant excess of L- over D-amino acids and stable isotope fractionation are also present, indicating that selective pressures and disequilibrium operate in interstellar chemistries [207,209].

A number of physicochemical processes have been proposed to account for the abiotic genesis of the organic material found in meteorites, and some of these processes have been confirmed as plausible in laboratory experiments modeling the corresponding environments [203,205,212-214]. One of the major classes of chemical reactions that may operate in space is the Fischer-Tropsch-type (FTT) reactions. The Fischer-Tropsch process [215,216] converts CO and H_2 _into liquid hydrocarbons in the presence of minerals and transition metal catalysts (Ni, Co, Fe) under conditions of high pressure and temperature:

(12) nCO + (2n + 1)H_2 _→ C_n_H_(2n + 2) _+ nH_2_O

The Fischer-Tropsch process involves a variety of competing reactions and usually leads to the synthesis of linear-chain alkanes of various lengths as a major product and alkenes, alcohols, and other oxygenated hydrocarbons as byproducts. The Fischer-Tropsch process is used industrially for the production of synthetic fuel and other petroleum products. Higher pressures and temperatures favor the production of longer alkane chains, whereas lowering the pressure at high temperatures leads to thermal decomposition of heavier alkanes to lighter products and methane. Different catalysts, reactants, and conditions favor different reaction routes, and the composition and distribution of the products of FTT reactions can be highly reminiscent of natural petroleum and the petroleum-like fraction of the organic material present in carbonaceous chondrites. Overall, laboratory experiments suggest that FTT reactions account for most principal features of meteorite organic matter, including higher hydrocarbons, nitrogen bases, amino acids, terpenoids, and porphyrin-like pigments [212,217,218].

The Fischer-Tropsch process is of special importance because it is proposed to be a major mechanism responsible for the genesis of petroleum according to the theory of the abiotic origin of petroleum [214,219,220]. It is commonly, but by no means universally, believed that petroleum is a "fossil" fuel in the sense that it originates from the biological conversion of sedimented detritus taking place on geological timescales. The proponents of the abiotic origin of petroleum point out, however, the difficulty of explaining how and why a highly reduced and complex mixture of hydrocarbons of high chemical potential (i.e., natural petroleum) is produced biologically from oxidized detritus of low chemical potential and preserved over extended periods of time in contact with living organisms. For living organisms are exceedingly adept at discovering and consuming energy/matter resources but are unlikely, for thermodynamic reasons, to account for the production of complex reduced hydrocarbons of high chemical potential [214,219]. Instead, it has been proposed that petroleum originates from primordial deposits and/or is produced in appropriate environments in the depths of the Earth by FTT processes in conditions of high pressure and temperature. Indeed, it was recently shown that a mixture consisting solely of solid iron oxide (FeO) and marble (CaCO_3_), 99.9% pure, and wet with triple-distilled water, evolves at 5 GPa and 1,500°C to yield methane, ethane, *n*-propane, 2-methylpropane, 2,2-dimethylpropane, *n*-butane, 2-methylbutane, *n*-pentane, 2-methylpentane, *n*-hexane, and *n*-alkanes through C_10_H_22_, ethene, *n*-propene, *n*-butene, and *n*-pentene in distributions characteristic of natural petroleum [214].

It is reasonable to speculate that, during the emergence and evolution of the solar system, fluctuations in pressure, temperature, and radiation taking place on multiple scales of space and time may have created conditions and environments permissive for Fischer-Tropsch-type reactions in space (e.g., see [221]). FTT reactions, in cooperation and competition with alternative chemistries such as photochemistry [205,222] may have enabled and shaped the evolution and expansion of diverse organic materials within the evolving circumstellar and, later, protoplanetary disks. As planets formed by collapse-like accretion of planetesimals, the FTT reactions, reactants, and products captured in supporting environments may have survived, adapted, and persisted. The FTT reactants and products ejected into environments with low pressure and high temperature may have evaporated or decomposed to methane and other light hydrocarbons, whereas the FTT products and reactants ejected into high-pressure and low-temperature environments may have been preserved as deposits. Abundant deposits of methane clathrates in seabeds and permafrost on the Earth, in the outer regions of the solar system, and, possibly, on Mars are consistent with this scenario. Moreover, recent analysis of the stable carbon and hydrogen isotopic composition of hydrocarbons in exhalates of the Lost City hydrothermal field indicates that hydrocarbons are produced abiotically in this type of submarine vents, most likely by FTT reactions. The latter discovery may mean that the abiotic synthesis of hydrocarbons by FTT processes is a previously unrecognized, constitutively active, and widespread source of life-essential building blocks in ocean-floor environments or wherever warm ultramafic rocks (i.e., rocks with low silica and high magnesium and iron content, such as basalt and the Earth's mantle) are in contact with water [223].

It should be pointed out that the proposed co-evolution of space chemistries and stellar/planetary systems is most likely a very general phenomenon and that the gravitational, thermal, radiation, and chemical processes accompanying stellar-planetary evolution are not independent phenomena but an intimately intertwined mix of interdependent forces that drive the self-organization of energy/matter into stellar and planetary systems. With this in mind, let us briefly review the geophysical history of our planet.

According to currently accepted views (for reviews, see [180,181]), terrestrial planets formed by the accretion of solid material that condensed from solar nebula. The Earth received its atmosphere from volatile compounds present within the planetesimals from which it was formed. The accretionary phase of the Earth's formation ended approximately 4.5 billion years ago (4.5 Ga) and was followed by the heavy bombardment period, during which Earth was repeatedly hit by meteorites of varying sizes, including large planetesimals (>100 km in diameter). The heavy bombardment ended about 3.8 Ga, and life was probably widespread by 3.5 Ga. During the accretionary phase, Earth's volatiles may have formed a transient steam atmosphere, which later rained down to form the ocean. The heavy bombardment period most likely witnessed repeated evaporation and condensation of Earth's water on various scales, including complete evaporation of the ocean and melting of the Earth's crust upon impact of especially large planetesimals. The early atmosphere may have been dominated by H_2_, which however was rapidly escaping to space, because hydrogen as a gas is too light to be significantly retained by Earth's gravity. The young Earth may have lost about a third of its ocean due to water dissociation and hydrogen outgassing [182]. Following the relative stabilization of the ocean-atmosphere system, Earth's atmosphere may have been dominated by carbon- and nitrogen-containing compounds, mainly CO_2_, CO, and N_2_, as is currently believed [180]. The presence of methane (CH_4_) and ammonia (NH_3_) in the atmosphere of early Earth would explain a number of geophysical observations and provide more support to the "primeval soup" theory of the origin of life, making the classic Miller-Urey experiments and follow-up work more meaningful [224,225]. However, whether these highly reducing gases were present in significant amounts in the early atmosphere is not clear, largely because of the apparent absence of active sources of CH_4 _and NH_3 _before the origin of life and the instability of methane and ammonia under UV irradiation.

On the other hand, if the aforementioned hypothesis of co-evolving chemistries and stellar/planetary systems is correct, the very early Earth may have indeed been highly reduced, being dominated not just by H_2 _but also by reduced forms of C, N, S, and other abundant elements, water being a reduced form of oxygen. This hypothetical, highly reduced Earth would have been very unstable (far from thermodynamic equilibrium) and subject to rapid at first and subsequently more gradual oxidation driven by the photodissociation of reduced chemical species (e.g., methane, ammonia, hydrogen sulfide, and water), molecular recombination, gravitational sorting, and the loss of hydrogen to space. In this scenario, molecular recombination processes, driven by solar radiation, mediated by small free radicals, and catalyzed by water, would lead to the formation and accumulation of increasingly stable oxidized molecular combinations, such as N_2_, O_2_, (H_y_)NO_x_, (H_y_)CO_x_, (H_y_)SO_x_, in the atmosphere and oceans of early Earth. These compounds would expand from the upper atmosphere to the lower atmosphere, the ocean, and the Earth's crust and upper mantle. Gradual oxidation of the Earth's atmosphere and upper mantle is indeed supported by some geological evidence, albeit it appears to be in conflict with other geological evidence [180,226]. It is possible, however, that the presumed conflict is imaginary, being a consequence of the habitual error of interpreting geophysical phenomena in terms of equilibrium thermodynamics and, thus, imagining a homogeneous, unstructured, and concentration- and diffusion-driven oxidation of the Earth (as the meaning of the word "gradual" conventionally implies). In reality, as discussed below, the planet Earth was, is, and always will be a multiscale system of structured energy/matter circulation driven by interacting gravitational, thermal, chemical, redox, and other gradients. Therefore, oxidation and reduction processes on the Earth have always been taking place in a non-uniform and relatively structured manner (through multiscale, turbulence-like convection), allowing for the spatiotemporal co-existence and high heterogeneity of reductive and oxidative compartments, fluxes, and processes. This notion may reconcile a great deal of the seemingly conflicting geophysical evidence and theories, which are perceived as competing alternatives within the interpretational framework of equilibrium thermodynamics, but which appear as cooperating complements when planetary phenomena are reinterpreted in terms of nonequilibrium thermodynamics.

Let us, therefore, leave the conventional interpretational framework and reconceptualize stellar and planetary evolution in the framework of the SOFT-NET interpretation. According to the SOFT-NET theory, the Universe as a whole represents a nonequilibrium process of energy/matter flow, and the emergence of a planet (or a star or galaxy) represents an organizational state transition within this flow. In the process and as a result of such an organizational transition, energy/matter is captured and transformed, via condensation and ordering, into a planetary (or stellar or galactic) structure-process. The latter emerges and evolves as a relatively condensed, structured flow/circulation pattern of interconverting energy/matter forms, which evolves in accordance with the empirical laws of nonequilibrium thermodynamics. The solar system as a whole and the planet Earth as its part formed via condensation of the interstellar medium of specific (inherited) chemical composition, where hydrogen was by far the most abundant species. Since this condensation occurred under conditions of high pressure, temperature, and radiation and in the presence of abundant small radicals promoting molecular recombination, the overall redox state of the original condensed material within the solar system was likely highly reduced. Indeed, according to a prominent astrophysicist, "hydrocarbons clearly are plentiful not only on all the gaseous major planets but also on the solid bodies (the large satellites, numerous asteroids, the planet Pluto, comets and meteorites); and there is every reason to believe that hydrocarbon compounds were incorporated in all of the planetary bodies at their formation" [227]. What is true for C and O is also likely to be true for other abundant elements, such as N and S. Next, it is reasonable to suggest that the differentiation of the newborn Earth into a structured core-mantle-crust-ocean-atmosphere system is just another organizational state transition in an ongoing self-organizational process of stellar/planetary evolution, where thermal, gravitational, chemical, and redox gradients act as major driving and shaping forces. Once formed, the differentiated planetary structure continues to be an evolving nonequilibrium process, which is driven by interdependent gradients and conjugated fluxes of interconverting energy/matter forms.

Currently, it is estimated that the Earth's temperature changes from 6,000°C (approximately the temperature of the Sun's outer layer) at the inner core to -100°C in the mesosphere in a matter of 6,000 km. The heat sustaining this thermal gradient originates in part from the primordial impact heat of accretion and in part from radioactive decay within the Earth's interior [180,181]. This thermal gradient, together with the Earth's gravitational gradient, together with the physical forces originating from the Earth's rotation and its movement in the gravitational fields of the Moon, the Sun, and other planets, together with solar radiation, Earth's biogeochemical gradients, and other internal and external influences, drive plate tectonics, volcanism, tides, winds, upwelling, and other interdependent convection and circulation processes taking place on multiple spatiotemporal scales in the Earth's mantle, crust, oceans, and atmosphere. In other words, the planet as a whole exists and evolves as a highly dynamic structure-process of energy/matter flow/circulation, where energy/matter, in its various forms, circulates on multiple scales of time and space simultaneously, from attoseconds and femtometers to billions of years and thousands of kilometers. Whereas the thermal and gravitational gradients, once established, have been changing slowly over macroevolutionary timescales, the Earth's redox gradient along the core-mantle-crust-ocean-atmosphere axis has increased dramatically, initially due to the photodissociation of reduced chemical species, gravitational sorting, and the loss of hydrogen to space and later due to photosynthesis. It is fair to suggest that the global redox gradient has increased over macroevolutionary timescales because it is coupled to and fueled by the flux of solar radiation. Photolysis of water (i.e., reduced oxygen) and other reduced species during the early stages of Earth's evolution and, later on, photosynthesis (which, in essence, is an advanced technology for photolysis of water and other reduced species) represent the two major mechanisms responsible for the dramatic increase of the redox gradient along the Earth's core-mantle-crust-ocean-atmosphere axis.

Geochemical cycles of carbon, nitrogen, sulfur, oxygen, and other elements can thus be seen as intertwined circulatory processes that mediate energy/matter flow/circulation within an evolving nonequilibrium system of interdependent gradients and conjugated fluxes of interconverting energy/matter forms (i.e., the planet). In the course of Earth's evolution, living organisms and their larger scale communities (conceptualized as self-organizing, nonequilibrium electron transport networks that support and, at the same time, are supported by electron flow) emerged to accelerate electron flow down the increasing Earth's redox gradient. Once emerged, living organisms and their larger scale organizations have been replacing the pre-existing, poorly structured, and inefficient geochemical cycles of oxidation and reduction with organized and vastly more efficient biogeochemical pathways of electron flow/circulation. In a conceptually analogous way, Benard cells emerge via self-organization to accelerate heat flow down an increasing temperature gradient, replacing relatively unstructured conduction with organized convection (Figure 1). Once emerged, living organisms and their communities have been co-evolving with the planet, accelerating and structuring the geochemical-turned-biogeochemical cycles. Consequently, most if not all of the structures and processes comprising living organisms and their larger-scale structures/organizations mediate, support and at the same time, are supported by moving electrons flowing down the Earth's redox gradient, which in turn is powered by solar radiation and other forces. In the course of biogeochemical evolution, the planetary-wide structure-process of electron flow has acquired an integrated, hierarchical, multiscale organization comprising molecules, cells, organisms, and ecosystems. This integrated system/organization/organism of structured electron flow/circulation also includes humans and their organizations, who are powered by the combustion (i.e., oxidation) of hydrocarbons extracted from the Earth and who are integrated into economies and societies by means of electrons continuously flowing and circulating through their energy grids, communication networks, and the Internet (and who, incidentally, plan to create a hydrogen economy, as if it were something new). The flow and circulation of electrons are driven by human technologies that exploit a variety of pre-existing energy/matter gradients, including solar radiation, the primordial energy of nucleosynthesis, and the Earth's gravitational, thermal, chemical, redox, and other gradients.

The outlined conceptualization of the planet is consistent with the current views on the organization of extant biogeochemical cycles (for reviews, see [228-230]). As an example, consider carbon and nitrogen fluxes. It is estimated that, in the contemporary ocean, photosynthetic carbon fixation (i.e., carbon reduction) by marine phytoplankton leads to the formation of 45-50 gigatons of organic C per annum (which is about a half of the total annual C fixation on the planet). Approximately two thirds of the newly fixed carbon is funneled into food chains, and about a third of it is exported to the ocean interior for biological oxidation. Remarkably, such a massive carbon flux is driven by a phytoplankton biomass of about 1 gigaton of C, which constitutes only 0.2% of the photosynthetically active C biomass on Earth [228,229]. This is achieved by means of a very rapid turnover of the phytoplankton biomass, with the average turnover time being on the order of a week or less. In this way, CO_2 _is rapidly and actively pumped from the atmosphere by radiation-driven photosynthesis and funneled into the interlocked system of reduction-oxidation cycles manifested as food chains and interconnected ecosystems exchanging and interconverting various energy/matter forms. These interlocked food chains and ecosystems, which include both living and nonliving matter, are sandwiched between and connect the reduced (core-mantle-crust) and oxidized (mantle-crust-ocean-atmosphere) parts of the planet. The reduced core-mantle-crust system is the ultimate source of reductive power, whereas the oxidized mantle-crust-ocean-atmosphere system and the increasing biomass of the planet act as sinks for electrons. As an example, consider denitrification, defined as the consecutive reduction of oxidized nitrogen from nitrate (NO_3_^-^) to N_2 _and/or ammonia (NH_3_) through intermediate oxidative states of nitrogen, and nitrification, defined as the consecutive oxidation of reduced nitrogen from ammonia (NH_3_) and/or N_2 _to nitrate (NO_3_^-^) through intermediate oxidative states of nitrogen. Combined with biotic and abiotic nitrogen fixation, denitrification and nitrification constitute, in essence, an advanced biogeochemical technology that mediates electron flow from the reduced core-mantle-crust system to the oxidized mantle-crust-ocean-atmosphere system and into biomass by means of nitrogen redox cycling. The role of living organisms and their higher order organizations within this biogeochemical technology is to structure and accelerate the electron and proton fluxes associated with nitrogen flow/circulation [230]. Notice that such a role of living organisms is conceptually analogous to the role of convection cells in the Benard instability example. Pertinently, even the physical transport of carbon and nitrogen is organized and facilitated to a large degree by the efforts of living organisms. As an example, oceanic diatoms undergo cyclic vertical migrations at great depths: down, to reach nutrient-rich waters, and up, to exploit the photoenergy-rich surface. As a result of this cyclic migration, nitrate is actively pumped from the ocean depths into the surface water, while surface-derived carbon is respired during migration, with the overall process accelerating the flux of nitrogen into the surface layer and the flux of carbon out of the surface layer [231].

The spatiotemporal organization, heterogeneity, and intimate interdependence of the living and nonliving processes and the energy/matter fluxes mediating electron flow along the Earth's redox gradient can be appreciated from the following examples. The nitrogen cycle controls the availability of nitrogenous nutrients and biological productivity in marine systems and, thus, is linked to the fixation of atmospheric carbon dioxide and the export of carbon from the ocean's surface [228,230]. Net primary production, defined as the photosynthetically fixed carbon available for other trophic levels, is distributed highly unevenly in the ocean, with large regions of low production (e.g., central ocean gyres) and smaller areas of high production (e.g., estuarine and upwelling regions). Satellite measurements indicate that chlorophyll concentrations qualitatively correlate with oceanic circulation and mesoscale physical processes such as coastal upwelling, eddies, and wind or convective mixing, as these influence the fluxes of essential nutrients from the subsurface nutrient reservoir into the photosynthesizing zone. Based on the highly conserved mean elemental composition of marine organic particles (106C/16N/1P), Alfred Redfield suggested in the 1950s that phosphate (P) may limit primary production in the ocean assuming that N_2 _fixation keeps pace with the photoautotrophic demand, because P has no biological or atmospheric source and is supplied to the ocean largely from fluvial sources (i.e., by rivers and streams). Redfield proposed that the maximum concentration of fixed N in the ocean is determined by the availability of P [228,232]. Recently, it has become apparent that N_2 _fixation itself is likely limited by availability of Fe [233]. Before the emergence of oxygenic photosynthesis, the ocean was presumably anoxic and mildly reducing, with estimated Fe concentrations of 25 mM [234]. As reduced (ferrous) Fe is soluble, whereas oxidized (ferric) Fe is virtually insoluble, concentrations of soluble Fe in the contemporary ocean rarely exceed the nanomolar range. Wind-blown, terrestrial dust is currently a major source of Fe for the ocean [228]. This wind- and soil-dependent flux limits phytoplankton carbon fixation throughout much of the contemporary Pacific Ocean [235]. The aeolian flux of Fe to the ocean is influenced by the water cycle, which in turn, is strongly influenced by atmospheric radiative forcing, i.e., by the balance between absorbed and radiated energy [228]. The transport of aeolian Fe is also related to wind speed and direction, which are related to the temperature contrast between the continents and the ocean. Silica supply, essential for diatom blooms, is largely dependent on riverine fluxes and upwelling from the ocean interior. Food-web structure, a strong determinant of how organic matter flux partitions in oceans among the microbial loop, the grazing food chain, sinking fluxes, and stored, dissolved organic matter [236], is critically dependent on mesoscale geophysical processes. Last but not least, humans have become a major transforming force on the planet. Powered by the oxidation of Earth-derived hydrocarbons, the growing (in size and complexity) economies of the industrial world represent, in essence, self-organizing nonequilibrium structures-processes that are supported by and, at the same time, support and accelerate electron flow down the Earth's redox gradient. Extraction and exploitation of natural resources and the dumping of waste into the environment represent the energy/matter exchanges that couple human society to the planetary-wide system of energy/matter circulation.

To summarize, the planet Earth represents a self-organizing, nonequilibrium, multiscale structure-process of energy/matter flow/circulation, which is driven by interdependent and ultimately inseparable nonliving and living processes, where living matter plays an increasingly influential organizing role. In turn, the planet Earth is an integral and inseparable part of the self-organizing and evolving Universe. According to the SOFT-NET theory, the process of self-organization is scale-invariant and proceeds through sequential organizational state transitions, in a manner characteristic of far-from-equilibrium systems, with macrostructures-processes emerging via condensation and self-organization of microstructures-processes. Once they have emerged as a result of an organizational transition, newborn structures-processes strive to persist and expand, growing in size/number, diversity, complexity, and order, while feeding on pre-existing energy/matter gradients. Economic competition among alternatively organized structures-processes feeding on the same energy/mater gradients leads to the elimination of economically deficient or inferior structure-processes and the improvement, diversification, and specialization of survivors, who are forced to fill and exploit all the available resource niches (the Darwinian phase of self-organization [6]). Promoted by mutually profitable exchanges of energy/matter, the self-organization of specializing survivors (structures-processes) into larger scale structures-processes transforms (mostly) competing alternatives into (mostly) cooperating complements. As a result, Darwinian competition is transferred onto a larger spatiotemporal scale, where it commences among alternative organizations of self-organized survivors (the organizational phase [6]). Such an economy-driven, scale-invariant process of self-organization leads to the emergence of increasingly long-lived, multiscale, hierarchical organizations (structures-processes) that expand over increasingly larger scales of space and time, feeding on available energy/matter gradients and eventually destroying them. Yet because energy/matter exists as a nonequilibrium system of interdependent gradients and conjugated fluxes of interconverting energy/matter forms, new gradients and fluxes are created and become dominant as old gradients and fluxes are consumed and destroyed. Such processes are responsible for the continuous birth, death, and transformation of energy/matter forms. Notice an uncanny conceptual analogy with the image of the world painted by great Eastern religions and philosophies such as Hinduism, Buddhism, and Taoism and by great Western philosophers such as Heraclitus and Hegel. Because the self-organizing, nonequilibrium Universe as a whole appears to grow in size and complexity over time, the SOFT-NET theory predicts the existence of a source(s) of and sink(s) for the energy/matter that flows through, supports, and is supported by the self-organizing Universe. The SOFT-NET interpretation also implies that there is no divide between living and nonliving matter and that the adjectives "living" and "nonliving" refer only to a difference in the organizational state of energy/matter. Because emerging macrostructures-processes inherit pre-existing microstructures-processes, metabolism (defined as a structured process of energy/matter flow/circulation realized via consumption, transformation, and exchange of energy/matter forms) is the true essence of inheritance. Genes are likely only a tool, an advanced technology for the preservation of useful forms, one which may have emerged relatively late in the evolution of life.

Concluding this section, the quest for an origin-of-life theory may have been misguided from the very beginning. According to the SOFT-NET interpretation, the origin of life coincides or precedes the emergence of the Universe, which has been evolving since its birth via condensation and self-organization of microstructures-processes into macrostructures-processes in a scale-free and self-similar manner. What is conventionally recognized as living matter appears to be a special organizational state of nonliving matter, which emerges under certain circumstances as a result of the evolution and self-organization of nonliving matter. Therefore, let us reformulate the question of the origin of life by asking the following. What is the nature of this special organizational state that endows energy/matter with the properties of the living? And under which circumstances is this state (i.e., living matter) likely to emerge?

The term 'emergence' implies a spontaneous and relatively rapid event of self-organization, i.e., an organizational state transition, which is a discontinuous event. Unfortunately, modern biology, being rooted in the interpretational frameworks of classical physics, has no conceptual handle on discontinuous evolutionary phenomena. Discontinuous evolutionary phenomena can be defined as self-organizational events in which multiple components are brought together to form a complex functional system, in which the interdependencies among components are such that no evolutionary advantage is achievable if individual components are introduced into an emerging system in a sequential manner (i.e., gradually) but, instead, only if they condense together in an all-or-none, phase transition-like manner. Discontinuous evolutionary phenomena require no or very few intermediate forms and thus leave paradoxical "gaps" in evolutionary records. One such discontinuous evolutionary phenomenon is the spontaneous emergence of the first living cell. As a rule, existing origin-of-life theories do not address or even mention this problem, as it seems so impenetrable within the conceptual frameworks of equilibrium thermodynamics and classical mechanics. On the contrary, all-or-none organizational state/phase transitions leading to the spontaneous emergence of complex, multicomponent structures-processes are an inherent and essential part of self-organizational dynamics in nonequilibrium physicochemical systems.

According to the empirical laws of nonequilibrium thermodynamics, organizational state/phase transitions naturally take place when an increasing energy/matter gradient exceeds a certain threshold value. Taking into account the key role of electron flow in living matter and looking at the conceptual model of the living organization outlined in Figure 2, one can infer that the environments conducive to the spontaneous emergence of the living cell, conceptualized as a persistent multicomponent electron transport network that supports and, at the same time, is supported by electron flow, should include the following features: i) a steep and increasing redox gradient as part of a system of intertwined energy/matter gradients and fluxes, ii) high densities of highly diverse redox-active molecular species and animate media (i.e., multiconformational, electron-conducting, and electron-storing molecular structures), iii) chemistries that would continuously generate and destroy electronically active molecular species and animate media, thus allowing for the recombination, selection, and evolution of diverse molecular structures and processes, and iv) water. It is not difficult to see that such environments are likely to be found at the interface between environments of high pressure, temperature, and radiation and environments of low pressure, temperature, and radiation, i.e., within the Earth's mantle-crust-ocean layer, where the reduced core-mantle-crust system acts as a source of electrons, the oxidized mantle-crust-ocean-atmosphere serves as a sink for electrons, and where high-pressure, high-temperature, and high-radiation chemistries (exemplified by FTT processes and photochemistry) create, support, and shape evolving molecular diversities under steady-state, flow-through conditions.

### "The deep, hot biosphere"

In the early 1990s, Thomas Gold suggested the existence of widespread microbial life populating the Earth's depths, in mass and volume comparable to or even greatly exceeding the whole of surface life [227]. According to Gold's hypothesis, the energy and matter sustaining what he called the "deep, hot biosphere" are derived from chemical processes by combining the liquids and gases that continuously seep upward from the Earth's interior through pores, cracks, and crevasses in the crust with substances available in local rocks and seawater [227]. This chemolithoautotrophic life presumably feeds on the chemical disequilibrium maintained due to the high chemical potential of the materials moving from the Earth's interior into new chemical environments. In support of his hypothesis, Gold pointed out the then-recently discovered submarine hydrothermal vents harboring rich and complex microbial and invertebrate ecosystems, which are rooted in chemolithoautotrophy and which are independent of solar energy and surface circumstances. The fact that hydrocarbons and bacteria are ubiquitous in the Earth's crust, as seen in numerous drilling, mining, and tunneling operations, including deep-drilling projects, and the phylogenetic evidence suggesting that the most primitive and ancient bacteria were thermophiles prompted Gold to propose that life may have originated somewhere in the depth of the Earth and then spread laterally and vertically, adapting to and populating new environments. Such a scenario would account for the presence of biological molecules in all carbonaceous materials in the outer crust, implying that natural petroleum may indeed have abiotic origins.

There have been advances and discoveries since the time Gold put forward his hypothesis. Studies of submarine hydrothermal vents revealed a vast and previously unknown domain of chemistry on Earth. Vent systems are widespread and represent primordial, chemically active environments, abundant in diverse reactive fluids, gases, and dissolved elements, where multiscale thermal, pH, and chemical gradients support sustained prebiotic synthesis [237]. Some of the discovered vents have been active for thousands of years. Others are relatively young. There are also examples of ancient, fossilized vents containing microfossils, some as old as 3.2 billion years of age [238]. Serpentinization, a geochemical process by which seawater and dissolved carbonates and sulfates invading warm or hot oceanic crust are converted into hydrogen, hydrocarbons, and hydrogen sulfide by reacting with Fe^2+^-containing rocks, has been proposed to be one of the major chemistries continuously delivering energy/matter for primary production in submarine ecosystems [237,239]. The discovery of an obligately photosynthetic bacterial anaerobe from a deep-sea hydrothermal vent suggests that volcanic or hydrothermal light can be harvested to drive photosynthetic reactions and that hydrothermal radiation can support microbial life in the absence of sunlight [240]. Moreover, it has been proposed that oxygenic photosynthesis may have hydrothermal origins, as the absorption spectra of bacteriochlorophylls closely match the calculated spectrum of thermal emission in the vicinity of hydrothermal vents [241]. The discovery of red-shifted chlorophylls in free-living and widely distributed organisms suggests that infrared radiation can be and is used for photosynthesis [242,243]. Another relevant example is the recent discovery of a novel metabolic pathway allowing for methane oxidation by oxygen in the absence of free oxygen, as oxygen is produced as an intermediate from nitrogen oxides [244]. This discovery is consistent with the hypothesis that nitrogen oxides may have been a major electron sink on the early anoxic Earth [245]. The existence of multiple biological pathways for the production of oxygen, such as detoxification of reactive oxygen species, chlorate respiration, and the production of oxygen from nitrogen oxides, may mean that oxygen was used in microbial metabolism long before the evolution of oxygenic photosynthesis. In other words, oxidation of substrates and photosynthesis do not require free oxygen and solar radiation as necessary prerequisites, for the point is to move electrons and protons, and this can be and is achieved by a variety of means. Indeed, a great variety of oxides, including nitrogen oxides, carbon oxides, sulfur oxides, iron oxides, and water (hydrogen oxide), are either known to serve or can potentially serve as electron sinks. By analogy, one may assume the existence of an equally great variety of mechanisms for harnessing diverse types of radiation energy to move electrons and protons. Some are already known, and others have yet to be described. The discovery of microbial populations anaerobically oxidizing methane and thermogenic higher hydrocarbons at the depth of 1.6 km below the sea floor in 100-million-year-old marine sediments maintained at 60°C to 100°C suggests that microbial consortia capable of anaerobic oxidation of methane and higher hydrocarbons may dominate deep and hot sediments, wherever there are thermogenic energy sources [246]. The remarkable metabolic versatility and adaptability of microorganisms, which allow them to thrive in conditions of extreme temperatures (from < -5°C to > 113°C), pressure (> 0.1 GPa), salt (> 1.5 M NaCl), pH (from < 0 to > 11), and radiation, continue to bring surprises, pushing the physicochemical limits of life to previously unimaginable extremes [247]. Interestingly, some microorganisms are able to grow continuously at 6,000 rads/hour or to survive acute irradiation doses of 1,500,000 rads [248], and other microorganisms exhibit "radiotropism," i.e., directional growth toward sources of ionizing radiation [99,100]. These observations present an apparent evolutionary paradox for conventional biology because, with the exception of a few natural uranium deposits, the radiation levels on the Earth's surface, including waters containing dissolved radionuclides, have provided only about 0.05 to 20 rads/year over the last 4 billion years [248].

It is also important to keep in mind that, due to the technological and methodological difficulties associated with studying microorganisms in the Earth's depths and/or in extreme conditions, the actual diversity and extent of the "hot, deep biosphere" will necessarily remain grossly underappreciated until appropriate research technologies and methods are developed and introduced. Even in the case of readily accessible marine environments, the true extent of marine microbial life and its contribution to biogeochemical cycles is only now becoming appreciated, as many highly abundant microbes, being extremely small and uncultivable, avoided detection and study for a long time, awaiting the introduction of novel research technologies and methods [236,249]. Based on the 16S rRNA sequence and metagenomic analyses, it is estimated that approximately 99% of the whole gene pool of prokaryotes remains unknown [247] and that only 0.1% of the existing prokaryotes have been cultured so far [250]. Pertinently, a major factor responsible for the low efficiencies of standard cultivation techniques (estimated to be between 0.001% and 1%) is the disruption of both biotic and abiotic interactions of microorganisms upon their isolation from their natural environments for culturing in the laboratory [250], i.e., their uncoupling from the global network of energy/matter flow/circulation. This fact suggests that culturing microorganisms in the laboratory faces difficulties that are conceptually analogous to the difficulties of culturing differentiated cells isolated from a multicellular organism.

To summarize, experimental reality in various research fields, including biochemistry, molecular and cell biology, microbial ecology, oceanography, biogeochemistry, and geophysics, is consistent with the idea that the living state/cell may have originated in the depths of the Earth. Because, according to the SOFT-NET theory, energy/matter evolves via condensation and self-organization of microstructures-processes into macrostructures-processes and because, physicochemically speaking, the living cell represents a condensed multicomponent phase, animated and held together by moving electrons, the *de novo *emergence of the living state/cell from nonliving molecules should involve condensation processes (phase transitions) and cooperative phenomena taking place in conditions of relative plasticity in electronic structures of reactants and in electronic transitions to new ground states. Fittingly, such phenomena are observed in materials at ultrahigh pressures.

High-pressure chemistry is a relatively young research field and a promising technology for generating new unusual materials of potentially high technological and commercial interest, including high-temperature superconductors, materials with non-linear optical properties, and superhard materials. High-pressure chemistry also represents a valued research tool in physics and chemistry for exploring unusual phases and states of matter (for reviews, see [251-253]). By lowering volume, pressure changes intermolecular and interatomic distances in compressed materials and, thus, can drastically alter pathways of chemical transformations and the physicochemical properties of compressed materials. At the fundamental level, compression changes the energetics of electrons, forcing them into states of lower kinetic energy, as their kinetic energy rises steeply upon compression. In general, this leads to the destabilization of intramolecular bonds and to electronic rearrangement of compressed molecules, thus opening reaction routes inaccessible at ambient conditions and promoting phase transitions to unusual states of matter [251]. The evolution of molecular bonds toward their ultimate destruction at sufficiently high density is a complex and poorly understood process. It can occur over a wide range of pressures and is often accompanied by unexpected intermediate states, including plasma-like phases. High pressure can also be used as a form of activation energy to promote molecular recombination and polymerization [254,255]. Pertinently, the drastic pressures required for spontaneous chemical transformations can often be lowered by photoactivation of reactions using relatively low irradiation energies because of the red shift of the electronic transitions with pressure [255-257]. Even more pertinently, water appears to be a powerful high-pressure photoactivatable reactant and radical initiator, able to trigger chemical reactions even with such stable molecules as N_2 _at pressures of a few tenths of a GPa upon near-UV irradiation at room temperature [255,257]. Altogether, since high-pressure chemistries can i) generate highly diverse organic materials from inorganic matter, including virtually all compound classes present in living cells, ii) enable a great variety of pathways for chemical transformation, and iii) morph materials into diverse organizational states/phases, high pressure may play a key role in the spontaneous emergence of the special organizational state/phase of energy/matter commonly recognized as living matter.

It is generally underappreciated that life on Earth has been evolving within a pressure gradient spanning more than seven orders of magnitude, from 0.03 MPa at the summit of Mt. Everest to the estimated 360 GPa at the center of the Earth. Pressure increases by about 10 MPa per kilometer in water column, reaching > 0.1 GPa in the deepest regions of the ocean. Likewise, pressure increases by about 30 MPa per kilometer within the Earth's crust, reaching > 2 GPa under the thickest crust. Whereas most of the deep ocean is cold (around 3°C below the thermocline at 30-100 meters of depth), the temperature in the Earth's crust increases on average by 25°C per every kilometer in depth [258]. Assuming that the high temperature limit for living cells is in the range of 100-150°C, the deepest environments compatible with life, as we know it, are at the depth of 5 to 10 km, i.e., under pressures and temperatures conducive to a variety of high-pressure chemistries. Moreover, it should be pointed out that, although averages, linear approximations, and the layered-cake-like diagrams of the Earth's structure are somewhat helpful, they are tools and representations from the province of classical physics, and thus are ultimately misleading, especially when used as assumptions for making inferences. In reality, being a nonequilibrium physicochemical system, the Earth's mantle-crust-ocean layer is expected to exist as a highly heterogeneous and relatively structured, multiscale spatiotemporal system of energy/matter circulation, a convection flow pattern, where diverse environments of widely different pressures, temperatures, and chemistries co-exist and co-evolve as relatively isolated yet interacting and interdependent compartments (not unlike microcompartments inside a cell). Therefore, it is reasonable to suggest that living matter may have been evolving on our planet in accordance with the following scenario.

In the course of its formation, the planet Earth inherited certain high-pressure, high-temperature, and high-radiation chemistries, together with the corresponding reactants and catalysts, from the preceding stages of energy/matter evolution, i.e., from evolving interstellar medium and protostellar and protoplanetary disks. Some of the inherited chemistries, reactants, and catalysts "died off," while others adapted, survived, and evolved in the depths of the planet. There, they continue to operate within permissive environments/compartments, generating diverse chemical species and organizational states/phases. The corresponding environments/compartments continuously produce and shed organic and inorganic materials in various forms and organizational states/phases, including living matter, somewhere along the borders separating the areas of high pressure, temperature, and radiation and those of low pressure, temperature, and radiation. The shed living matter, feeding on the planetary redox gradient and chemical disequilibrium, ventures outward to face new physicochemical environments as well as the struggle for survival with nonliving elements and the living forms that came in earlier. Metaphorically speaking, there is a fire of life bursting inside the planet that sheds off fire swirls. Most of the swirls vanish, but those that survive and thrive coalesce and expand the fire of life.

### On the nature of consciousness

**Figure 4:**
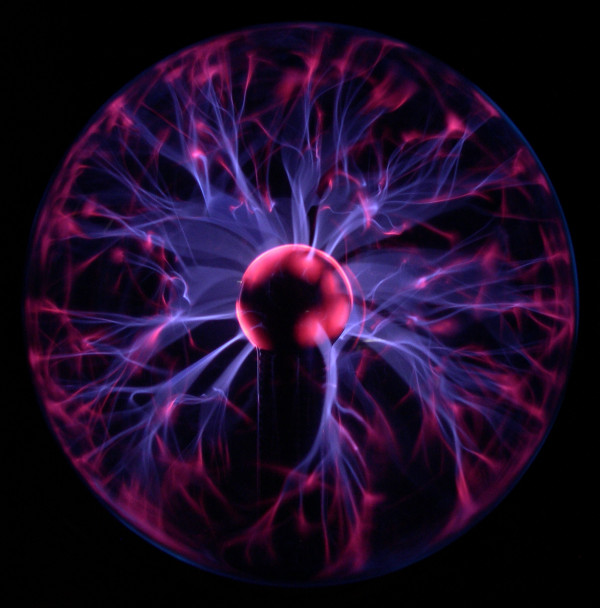
**Plasma lamp**. The SOFT-NET theory postulates scale-invariance of the self-organizational dynamics of energy/matter at all levels of the organizational hierarchy, from elementary particles through cells and organisms to the Universe as a whole. The presented analysis of empirical data, together with the inferences made on the basis of scale symmetry, suggest that electrons moving between ionized centers in far-from-equilibrium conditions are responsible for key properties of living matter. The plasma lamp conveniently illustrates some of the physical processes and self-organizational patterns that appear to be shared by proteins, cells, organisms, living planets, and plasmas. It should also be mentioned that the hydrated electron was recently proposed to have a similar spatial organization [271]. The image is courtesy of Luc Viatour [272]; see also [260] for more details on plasmas.

It is tempting to speculate that, at the fundamental level, living matter originates from a plasma-like organizational state/phase of energy/matter and that it preserves essential properties of the parental plasma-like state/phase as it expands over increasingly larger spatiotemporal scales in the form of an evolving multiscale hierarchical organization of energy/matter flow/circulation. Indeed, plasmas and the electron transport networks comprising living matter share a remarkable number of essential physical processes, structural elements, and organizational patterns (Figure 4). In both cases, electrons move between ionized centers, and in both cases, ionized centers are situated close enough to influence many nearby neighbors, a property that leads to collective effects and behaviors, a distinguished feature of both plasmas and living matter. Both plasmas and living matter spontaneously form spatiotemporal structures on a wide range of scales. Interestingly enough, plasmas form spatiotemporal structures that are highly reminiscent of cellular structures, such as, for example, filaments and double layers. The latter involve localized charge separation, which causes a large potential difference across the double layer but does not generate an electric field outside it. Double layers separate adjacent plasma regions with different physical characteristics. In fact, there are so many similarities between double layers in plasmas and cellular membrane bilayers in terms of the organizational behavior of electrons and ions that these similarities are unlikely to be coincidental [259-261]. This means that the physics of double layers in plasmas may provide a fresh and powerful paradigm for understanding the biophysics of cellular membranes and associated phenomena, such as membrane permeability, membrane potential, ion partitioning, and action potential, which, contrary to common belief, are far from understood and remain the subject of a hidden, yet longstanding controversy (e.g., see [262-264]). It is uncanny that the cellular membrane is also called the plasma membrane, while the original term for the contents of living cells is "protoplasm," coming from the Greek *protos *for *first *and *plasma *for *a thing formed/molded *[265]. Both plasmas and living matter are quasineutral. Quasineutrality in plasma requires that plasma currents close on themselves in electric circuits (electron flow/circulation). These circuits form a strongly coupled system, with the behavior in each plasma region dependent on the entire circuit. Strong coupling between system elements, together with instabilities and nonlinearities, leads to the emergence of complex behaviors in both plasmas and living matter [260]. Most importantly, although the parameters of disparate plasmas can vary by many orders of amplitude, certain basic properties and behaviors of plasmas are scale-invariant, allowing the prediction of behaviors of disparate plasmas using similarity transformations [266]. Plasmas are by far the most common phase of matter in the Universe, both by mass and by volume, and one of the most important discoveries in cosmology has been the cellular structure of space, i.e., the spontaneous self-organization of space into regions with different physical characteristics separated by "cell walls" in the form of sheets of electric currents [260,261].

There are, of course, differences between conventional plasmas and living matter. Plasmas are ionized gases, whereas living matter can be conceptualized as an ionized fluid-like condensed phase. Plasmas are electrically conductive, whereas living matter appears to exhibit both insulating and conductive properties and, thus, can be conceptualized as a disordered conductor. Another major difference between plasmas and living matter is that electrons within living matter, unlike electrons in plasmas, build, sustain, and shape metastable and increasingly longer-lived macromolecular and higher-order structures and organizations, within which they propagate, circulate, and exist. In the context of developing living matter, these structures act essentially as memories (old and/or continuously reproduced structures and behaviors) and ideas (new structures and behaviors), which allow for and facilitate the expansion of living matter in space and time by means of biological evolution and self-organization (see also [6,9]). Notice that in certain essential ways, humans and electrons are not that different: they both build, sustain, and shape higher-order organizations that in turn sustain and shape them, and they both work to shape and control their environments, which in turn shape and control them, with the overall result being the expansion of life in space and time.

It is reasonable to suggest, therefore, that individual living cells and organisms represent islands of a plasma-like organizational state/phase of energy/matter that are bounded and coordinated by electrons into functional, conscious wholes. These islands operate within, organize, and ultimately assimilate nonliving matter, thus expanding living matter/phase in space and time. Electrons have a number of physical properties that make them well suited to function as bounding and coordinating agents. Indeed, electronic wave functions can extend over and be correlated within a disordered electronic system of any size, provided that the system is in a critical state, poised between conducting and nonconducting phases [267]. Moreover, electron-electron interactions in disordered conductors tuned at the metal-insulator phase transition lead to self-organizational electronic phenomena manifested as quasiparticle excitations, which also exhibit diverging correlation length [268]. Because, as captured by the concept of self-organized criticality [269], living matter tends to spontaneously achieve and maintain criticality, which is, incidentally, reflected in the fractal organization (a signature of a critical state) pervading all levels of biological organizational hierarchy [8], the phenomenon of consciousness may have something to do with the interactions and collective self-organization of electronic states that are spread over, bind, and coordinate individual parts within and across scales into a multiscale self-conscious whole. In other words, consciousness is likely a self-organizational phenomenon as well and, thus, a natural consequence of the evolution and self-organization of nonliving matter. Notice that such an interpretation renders the notion of the spirit entering, inhabiting, animating, and leaving organisms simply a question of semantics. It also implies the existence of multiple levels of consciousness as well as deep-running physical connections, both existing and potential, between everything that comes into existence.

One of the important ramifications of the SOFT-NET interpretation is that living matter and consciousness, both being natural consequences of the evolution and self-organization of nonliving matter, may turn out to be highly abundant in the Universe at large. Moreover, because, as tacitly implied but left unaddressed, the currently accepted interpretations of space, time, energy, and matter are inaccurate, alien life forms may be not as far off as we currently believe. It is worth pointing out that, according to the SOFT-NET interpretation, the physicochemical composition and metabolism of alien life forms are expected to be similar to our own, since we share with all alien living matter a common physicochemical and metabolic ancestry in the form of the energy/matter composition and space chemistries that we have inherited and carry in our cells. At the same time, locality-specific variations in physicochemical environments and evolutionary histories across the Universe most likely give rise to a great diversity of alien life forms. Because the principles of self-organization of energy/matter are universal, the basic intentions and purposes of all aliens will be the same as those of humans, namely, the perpetuation and expansion of alien-specific organizational forms. In fact, the future relationships between humans and aliens can be inferred by reviewing the emergence and evolution of biological species and human races on the planet Earth. Whether we humans will become the Universe's "*Homo sapiens*" or another "species," "modern humans" or "Neanderthals," "colonists" or "natives," is up to our will and intelligence, for we and all other alien life forms have a common origin, inhabit the same environment, and draw on the same sources of energy and matter.

## Competing interests

The author declares that they have no competing interests.

## Authors' contributions

AK is the sole author of this paper and is responsible for developing the concepts and for writing and revising the manuscript.

## References

[B1] Leigh EG Jr Vermeij GJ Does natural selection organize ecosystems for the maintenance of high productivity and diversity? Philos Trans R Soc Lond B Biol Sci 2002 357 709 718 10.1098/rstb.2001.0990 12079531 PMC1692970

[B2] Vermeij GJ Nature: An Economic History 2004 Princeton, NJ: Princeton University Press

[B3] Nicolis G Prigogine I Self-organization in Nonequilibrium Systems 1977 New York: John Wiley & Sons, Inc

[B4] Prigogine I Stengers I Order out of Chaos. Man's New Dialogue With Nature 1984 New York: Bantam Books

[B5] Winfree AT Spatial and temporal organization in the Zhabotinsky reaction Adv Biol Med Phys 1977 16 115 136 551714 10.1016/b978-0-12-005216-5.50011-6

[B6] Kurakin A The universal principles of self-organization and the unity of Nature and knowledge 2007 http://www.alexeikurakin.org/text/thesoft.pdf

[B7] Kurakin A The 5th dimension 2008 http://www.youtube.com/watch?v=ZwyfYsw9VZ8

[B8] Kurakin A Scale-free flow of life: on the biology, economics, and physics of the cell Theor Biol Med Model 2009 6 6 10.1186/1742-4682-6-6 19416527 PMC2683819

[B9] Kurakin A Order without design Theor Biol Med Model 7 12 10.1186/1742-4682-7-12 20398287 PMC2867811

[B10] DeVault D Chance B Studies of photosynthesis using a pulsed laser. I. Temperature dependence of cytochrome oxidation rate in chromatium. Evidence for tunneling Biophys J 1966 6 825 847 10.1016/S0006-3495(66)86698-5 5972381 PMC1368046

[B11] DeVault D Parkes JH Chance B Electron tunnelling in cytochromes Nature 1967 215 642 644 10.1038/215642a0 6050223

[B12] Marcus RA Sutin N Electron transfers in chemistry and biology Biochim Biophys Acta 1985 811 265 322

[B13] Gray HB Winkler JR Electron tunneling through proteins Q Rev Biophys 2003 36 341 372 10.1017/S0033583503003913 15029828

[B14] Curry WB Grabe MD Kurnikov IV Skourtis SS Beratan DN Regan JJ Aquino AJ Beroza P Onuchic JN Pathways, pathway tubes, pathway docking, and propagators in electron transfer proteins J Bioenerg Biomembr 1995 27 285 293 10.1007/BF02110098 8847342

[B15] Farver O Pecht I Electron transfer in proteins: in search of preferential pathways FASEB J 1991 5 2554 2559 1868979 10.1096/fasebj.5.11.1868979

[B16] Henzler-Wildman K Kern D Dynamic personalities of proteins Nature 2007 450 964 972 10.1038/nature06522 18075575

[B17] Csermely P Palotai R Nussinov R Induced fit, conformational selection and independent dynamic segments: an extended view of binding events Trends Biochem Sci 2010 35 539 546 10.1016/j.tibs.2010.04.009 20541943 PMC3018770

[B18] Lockless SW Ranganathan R Evolutionarily conserved pathways of energetic connectivity in protein families Science 1999 286 295 299 10.1126/science.286.5438.295 10514373

[B19] Suel GM Lockless SW Wall MA Ranganathan R Evolutionarily conserved networks of residues mediate allosteric communication in proteins Nat Struct Biol 2003 10 59 69 10.1038/nsb881 12483203

[B20] Ota N Agard DA Intramolecular signaling pathways revealed by modeling anisotropic thermal diffusion J Mol Biol 2005 351 345 354 10.1016/j.jmb.2005.05.043 16005893

[B21] Agarwal PK Billeter SR Rajagopalan PT Benkovic SJ Hammes-Schiffer S Network of coupled promoting motions in enzyme catalysis Proc Natl Acad Sci USA 2002 99 2794 2799 10.1073/pnas.052005999 11867722 PMC122427

[B22] Goodey NM Benkovic SJ Allosteric regulation and catalysis emerge via a common route Nat Chem Biol 2008 4 474 482 10.1038/nchembio.98 18641628

[B23] Bogan AA Thorn KS Anatomy of hot spots in protein interfaces J Mol Biol 1998 280 1 9 10.1006/jmbi.1998.1843 9653027

[B24] Stites WE Protein-Protein Interactions: Interface Structure, Binding Thermodynamics, and Mutational Analysis Chemical Reviews 1997 97 1233 1250 10.1021/cr960387h 11851449

[B25] Lin J Balabin IA Beratan DN The nature of aqueous tunneling pathways between electron-transfer proteins Science 2005 310 1311 1313 10.1126/science.1118316 16311331 PMC3613566

[B26] de la Lande A Babcock NS Rezac J Sanders BC Salahub DR Surface residues dynamically organize water bridges to enhance electron transfer between proteins Proc Natl Acad Sci USA 2010 107 11799 11804 10.1073/pnas.0914457107 20547871 PMC2900646

[B27] Szent-Gyorgyi A Towards a New Biochemistry? Science 1941 93 609 611 10.1126/science.93.2426.609 17841996

[B28] Winkler JR Nocera DG Yocom KM Bordignon E Gray HB Electron-transfer kinetics of pentaammineruthenium(III)(histidine-33)-ferricytochrome c. Measurement of the rate of intramolecular electron transfer between redox centers separated by 15 A in a protein Journal of the American Chemical Society 1982 104 5798 5800 10.1021/ja00385a047

[B29] Nagel ZD Klinman JP Tunneling and dynamics in enzymatic hydride transfer Chem Rev 2006 106 3095 3118 10.1021/cr050301x 16895320

[B30] Hammes-Schiffer S Hydrogen tunneling and protein motion in enzyme reactions Acc Chem Res 2006 39 93 100 10.1021/ar040199a 16489728

[B31] Vendruscolo M Dobson CM Structural biology. Dynamic visions of enzymatic reactions Science 2006 313 1586 1587 10.1126/science.1132851 16973868

[B32] Antoniou D Caratzoulas S Kalyanaraman C Mincer JS Schwartz SD Barrier passage and protein dynamics in enzymatically catalyzed reactions Eur J Biochem 2002 269 3103 3112 10.1046/j.1432-1033.2002.03021.x 12084050

[B33] Eisenmesser EZ Millet O Labeikovsky W Korzhnev DM Wolf-Watz M Bosco DA Skalicky JJ Kay LE Kern D Intrinsic dynamics of an enzyme underlies catalysis Nature 2005 438 117 121 10.1038/nature04105 16267559

[B34] Henzler-Wildman KA Lei M Thai V Kerns SJ Karplus M Kern D A hierarchy of timescales in protein dynamics is linked to enzyme catalysis Nature 2007 450 913 916 10.1038/nature06407 18026087

[B35] Henzler-Wildman KA Thai V Lei M Ott M Wolf-Watz M Fenn T Pozharski E Wilson MA Petsko GA Karplus M Intrinsic motions along an enzymatic reaction trajectory Nature 2007 450 838 844 10.1038/nature06410 18026086

[B36] Cordes M Giese B Electron transfer in peptides and proteins Chem Soc Rev 2009 38 892 901 10.1039/b805743p 19421569

[B37] Chang CJ Chang MC Damrauer NH Nocera DG Proton-coupled electron transfer: a unifying mechanism for biological charge transport, amino acid radical initiation and propagation, and bond making/breaking reactions of water and oxygen Biochim Biophys Acta 2004 1655 13 28 10.1016/j.bbabio.2003.08.010 15100012

[B38] Frey PA Hegeman AD Reed GH Free radical mechanisms in enzymology Chem Rev 2006 106 3302 3316 10.1021/cr050292s 16895329

[B39] Murphy CJ Arkin MR Jenkins Y Ghatlia ND Bossmann SH Turro NJ Barton JK Long-range photoinduced electron transfer through a DNA helix Science 1993 262 1025 1029 10.1126/science.7802858 7802858

[B40] Boal AK Yavin E Barton JK DNA repair glycosylases with a [4Fe-4S] cluster: a redox cofactor for DNA-mediated charge transport? J Inorg Biochem 2007 101 1913 1921 10.1016/j.jinorgbio.2007.05.001 17599416 PMC2094209

[B41] Delaney S Barton JK Long-range DNA charge transport J Org Chem 2003 68 6475 6483 10.1021/jo030095y 12919006

[B42] Henderson PT Jones D Hampikian G Kan Y Schuster GB Long-distance charge transport in duplex DNA: the phonon-assisted polaron-like hopping mechanism Proc Natl Acad Sci USA 1999 96 8353 8358 10.1073/pnas.96.15.8353 10411879 PMC17521

[B43] Giese B Long-distance charge transport in DNA: the hopping mechanism Acc Chem Res 2000 33 631 636 10.1021/ar990040b 10995201

[B44] Seidel CAM Schulz A Sauer MHM Nucleobase-Specific Quenching of Fluorescent Dyes. 1. Nucleobase One-Electron Redox Potentials and Their Correlation with Static and Dynamic Quenching Efficiencies J Phys Chem 1996 100 5541 5553 10.1021/jp951507c

[B45] Sugiyama H Saito I Theoretical Studies of GG-Specific Photocleavage of DNA via Electron Transfer: Significant Lowering of Ionization Potential and 5'-Localization of HOMO of Stacked GG Bases in B-Form DNA J Am Chem Soc 1996 118 7063 7068 10.1021/ja9609821

[B46] Burrows C Muller J Oxidative Nucleobase Modifications Leading to Strand Scission Chem Rev 1998 98 1109 1152 10.1021/cr960421s 11848927

[B47] Merino EJ Barton JK DNA oxidation by charge transport in mitochondria Biochemistry 2008 47 1511 1517 10.1021/bi701775s 18189417

[B48] Merino EJ Davis ML Barton JK Common mitochondrial DNA mutations generated through DNA-mediated charge transport Biochemistry 2009 48 660 666 10.1021/bi801570j 19128037 PMC2668510

[B49] Merino EJ Boal AK Barton JK Biological contexts for DNA charge transport chemistry Curr Opin Chem Biol 2008 12 229 237 10.1016/j.cbpa.2008.01.046 18314014 PMC3227530

[B50] Friedman K Heller A On the Non-Uniform Distribution of Guanine in Introns of Human Genes: Possible Protection of Exons against Oxidation by Proximal Intron Poly-G Sequences J Phys Chem B 2001 105 11859 11865 10.1021/jp012043n

[B51] Friedman K Heller A Guanosine Distribution and Oxidation Resistance in Eight Eukaryotic Genomes J Am Chem Soc 2004 126 2368 2371 10.1021/ja038217r 14982441

[B52] Boal AK Genereux JC Sontz PA Gralnick JA Newman DK Barton JK Redox signaling between DNA repair proteins for efficient lesion detection Proc Natl Acad Sci USA 2009 106 15237 15242 10.1073/pnas.0908059106 19720997 PMC2741234

[B53] Augustyn KE Merino EJ Barton JK A role for DNA-mediated charge transport in regulating p53: Oxidation of the DNA-bound protein from a distance Proc Natl Acad Sci USA 2007 104 18907 18912 10.1073/pnas.0709326104 18025460 PMC2141881

[B54] Rajski SR Barton JK How different DNA-binding proteins affect long-range oxidative damage to DNA Biochemistry 2001 40 5556 5564 10.1021/bi002684t 11331021

[B55] Boon EM Salas JE Barton JK An electrical probe of protein-DNA interactions on DNA-modified surfaces Nat Biotechnol 2002 20 282 286 10.1038/nbt0302-282 11875430

[B56] Gorodetsky AA Ebrahim A Barton JK Electrical detection of TATA binding protein at DNA-modified microelectrodes J Am Chem Soc 2008 130 2924 2925 10.1021/ja7106756 18271589 PMC2747583

[B57] Hidalgo E Demple B An iron-sulfur center essential for transcriptional activation by the redox-sensing SoxR protein Embo J 1994 13 138 146 8306957 10.1002/j.1460-2075.1994.tb06243.x PMC394787

[B58] Lee PE Demple B Barton JK DNA-mediated redox signaling for transcriptional activation of SoxR Proc Natl Acad Sci USA 2009 106 13164 13168 10.1073/pnas.0906429106 19651620 PMC2726364

[B59] Lieberman-Aiden E van Berkum NL Williams L Imakaev M Ragoczy T Telling A Amit I Lajoie BR Sabo PJ Dorschner MO Comprehensive mapping of long-range interactions reveals folding principles of the human genome Science 2009 326 289 293 10.1126/science.1181369 19815776 PMC2858594

[B60] West GB Brown JH The origin of allometric scaling laws in biology from genomes to ecosystems: towards a quantitative unifying theory of biological structure and organization J Exp Biol 2005 208 1575 1592 10.1242/jeb.01589 15855389

[B61] Mandelbrot BB The Fractal Geometry of Nature 2006 New York: W.H. Freeman and Company

[B62] West GB Brown JH Enquist BJ A general model for the origin of allometric scaling laws in biology Science 1997 276 122 126 10.1126/science.276.5309.122 9082983

[B63] West GB Woodruff WH Brown JH Allometric scaling of metabolic rate from molecules and mitochondria to cells and mammals Proc Natl Acad Sci USA 2002 99 Suppl 1 2473 2478 10.1073/pnas.012579799 11875197 PMC128563

[B64] Branden M Sanden T Brzezinski P Widengren J Localized proton microcircuits at the biological membrane-water interface Proc Natl Acad Sci USA 2006 103 19766 19770 10.1073/pnas.0605909103 17172452 PMC1750901

[B65] Haines TH Anionic lipid headgroups as a proton-conducting pathway along the surface of membranes: a hypothesis Proc Natl Acad Sci USA 1983 80 160 164 10.1073/pnas.80.1.160 6296863 PMC393330

[B66] Haines TH Dencher NA Cardiolipin: a proton trap for oxidative phosphorylation FEBS Lett 2002 528 35 39 10.1016/S0014-5793(02)03292-1 12297275

[B67] Mulkidjanian AY Heberle J Cherepanov DA Protons @ interfaces: implications for biological energy conversion Biochim Biophys Acta 2006 1757 913 930 10.1016/j.bbabio.2006.02.015 16624250

[B68] Friedman R Nachliel E Gutman M Molecular dynamics of a protein surface: ion-residues interactions Biophys J 2005 89 768 781 10.1529/biophysj.105.058917 15894639 PMC1366628

[B69] Gutman M Nachliel E Friedman R The mechanism of proton transfer between adjacent sites on the molecular surface Biochim Biophys Acta 2006 1757 931 941 10.1016/j.bbabio.2006.01.012 16581015

[B70] Gopta OA Cherepanov DA Junge W Mulkidjanian AY Proton transfer from the bulk to the bound ubiquinone Q(B) of the reaction center in chromatophores of Rhodobacter sphaeroides: retarded conveyance by neutral water Proc Natl Acad Sci USA 1999 96 13159 13164 10.1073/pnas.96.23.13159 10557290 PMC23917

[B71] Reece SY Hodgkiss JM Stubbe J Nocera DG Proton-coupled electron transfer: the mechanistic underpinning for radical transport and catalysis in biology Philos Trans R Soc Lond B Biol Sci 2006 361 1351 1364 10.1098/rstb.2006.1874 16873123 PMC1647304

[B72] Stubbe J van Der Donk WA Protein Radicals in Enzyme Catalysis Chem Rev 1998 98 705 762 10.1021/cr9400875 11848913

[B73] Liang ZX Klinman JP Structural bases of hydrogen tunneling in enzymes: progress and puzzles Curr Opin Struct Biol 2004 14 648 655 10.1016/j.sbi.2004.10.008 15582387

[B74] Cukier RI A Theory for the Rate Constant of a Dissociative Proton-Coupled Electron-Transfer Reaction J Phys Chem A 1999 103 5989 5995 10.1021/jp990329a

[B75] Cukier RI A Theory that Connects Proton-Coupled Electron-Transfer and Hydrogen-Atom Transfer Reactions J Phys Chem B 2002 106 1746 1757 10.1021/jp012396m

[B76] Weatherly SC Yang IV Thorp HH Proton-Coupled Electron Transfer in Duplex DNA: Driving Force Dependence and Isotope Effects on Electrocatalytic Oxidation of Guanine J Am Chem Soc 2001 123 1236 1237 10.1021/ja003788u 11456681

[B77] Raytchev M Mayer E Amann N Wagenknecht HA Fiebig T Ultrafast proton-coupled electron-transfer dynamics in pyrene-modified pyrimidine nucleosides: model studies towards an understanding of reductive electron transport in DNA Chemphyschem 2004 5 706 712 10.1002/cphc.200301205 15179723

[B78] Gervasio FL Boero M Parrinello M Double proton coupled charge transfer in DNA Angew Chem Int Ed Engl 2006 45 5606 5609 10.1002/anie.200602106 16888729

[B79] Kumar A Sevilla MD Sugar Radical Formation by a Proton Coupled Hole Transfer in 2 2-Deoxyguanosine Radical Cation (2 2-dG "+): A Theoretical Treatment J Phys Chem B 2009 113 13374 13380 10.1021/jp9058593 19754084 PMC2765868

[B80] Krivokapic A Herak JN Sagstuen E Proton-coupled hole transfer in X-irradiated doped crystalline cytosine.H2O J Phys Chem A 2008 112 3597 3606 10.1021/jp709855c 18341308

[B81] Steenken S Electron transfer in DNA? Competition by ultra-fast proton transfer? Biol Chem 1997 378 1293 1297 9426189

[B82] Wikipedia Cosmic ray 2010 http://en.wikipedia.org/wiki/Cosmic_ray

[B83] Wikipedia Hydrogen 2010 http://en.wikipedia.org/wiki/Hydrogen

[B84] Wächtershäuser G Pyrite formation, the first energy source for life: a hypothesis System Appl Microbiol 1988 10 207 210

[B85] Fischer F Zillig W Stetter KO Schreiber G Chemolithoautotrophic metabolism of anaerobic extremely thermophilic archaebacteria Nature 1983 301 511 513 10.1038/301511a0 6401847

[B86] Ellis RJ Macromolecular crowding: obvious but underappreciated Trends Biochem Sci 2001 26 597 604 10.1016/S0968-0004(01)01938-7 11590012

[B87] Kurakin A Stochastic cell IUBMB Life 2005 57 59 63 10.1080/15216540400024314 16036564

[B88] Kurakin A Self-organization vs Watchmaker: stochastic gene expression and cell differentiation Dev Genes Evol 2005 215 46 52 10.1007/s00427-004-0448-7 15645318

[B89] Finney LA O'Halloran TV Transition metal speciation in the cell: insights from the chemistry of metal ion receptors Science 2003 300 931 936 10.1126/science.1085049 12738850

[B90] Halliwell B Gutteridge JM Oxygen toxicity, oxygen radicals, transition metals and disease Biochem J 1984 219 1 14 6326753 10.1042/bj2190001 PMC1153442

[B91] Johnson DC Dean DR Smith AD Johnson MK Structure, function, and formation of biological iron-sulfur clusters Annu Rev Biochem 2005 74 247 281 10.1146/annurev.biochem.74.082803.133518 15952888

[B92] Glaser T Hedman B Hodgson KO Solomon EI Ligand K-edge X-ray absorption spectroscopy: a direct probe of ligand-metal covalency Acc Chem Res 2000 33 859 868 10.1021/ar990125c 11123885

[B93] Wikipedia Riboflavin 2010 http://en.wikipedia.org/wiki/Riboflavin

[B94] Hill HZ The function of melanin or six blind people examine an elephant Bioessays 1992 14 49 56 10.1002/bies.950140111 1546980

[B95] Meredith P Sarna T The physical and chemical properties of eumelanin Pigment Cell Res 2006 19 572 594 10.1111/j.1600-0749.2006.00345.x 17083485

[B96] d'Ischia M Napolitano A Pezzella A Meredith P Sarna T Chemical and structural diversity in eumelanins: unexplored bio-optoelectronic materials Angew Chem Int Ed Engl 2009 48 3914 3921 19294706 10.1002/anie.200803786 PMC2799031

[B97] Olsen S Riesz J Mahadevan I Coutts A Bothma JP Powell BJ McKenzie RH Smith SC Meredith P Convergent proton-transfer photocycles violate mirror-image symmetry in a key melanin monomer J Am Chem Soc 2007 129 6672 6673 10.1021/ja069280u 17489592

[B98] Kono R Yamaoka T Yoshizaki H McGinness J Anomalous absorption and dispersion of sound waves in diethylamine melanin J Appl Phys 1979 50 1236 1244 10.1063/1.326143

[B99] Dadachova E Bryan RA Huang X Moadel T Schweitzer AD Aisen P Nosanchuk JD Casadevall A Ionizing radiation changes the electronic properties of melanin and enhances the growth of melanized fungi PLoS One 2007 2 e457 10.1371/journal.pone.0000457 17520016 PMC1866175

[B100] Dadachova E Casadevall A Ionizing radiation: how fungi cope, adapt, and exploit with the help of melanin Curr Opin Microbiol 2008 11 525 531 10.1016/j.mib.2008.09.013 18848901 PMC2677413

[B101] Giles NM Giles GI Jacob C Multiple roles of cysteine in biocatalysis Biochem Biophys Res Commun 2003 300 1 4 10.1016/S0006-291X(02)02770-5 12480511

[B102] Giles GI Jacob C Reactive sulfur species: an emerging concept in oxidative stress Biol Chem 2002 383 375 388 10.1515/BC.2002.042 12033429

[B103] Giles NM Watts AB Giles GI Fry FH Littlechild JA Jacob C Metal and redox modulation of cysteine protein function Chem Biol 2003 10 677 693 10.1016/S1074-5521(03)00174-1 12954327

[B104] Wu G Fang YZ Yang S Lupton JR Turner ND Glutathione metabolism and its implications for health J Nutr 2004 134 489 492 14988435 10.1093/jn/134.3.489

[B105] Griffith OW Biologic and pharmacologic regulation of mammalian glutathione synthesis Free Radic Biol Med 1999 27 922 935 10.1016/S0891-5849(99)00176-8 10569625

[B106] Danon A Redox reactions of regulatory proteins: do kinetics promote specificity? Trends Biochem Sci 2002 27 197 203 10.1016/S0968-0004(02)02066-2 11943547

[B107] Lillig CH Holmgren A Thioredoxin and related molecules--from biology to health and disease Antioxid Redox Signal 2007 9 25 47 10.1089/ars.2007.9.25 17115886

[B108] Aslund F Zheng M Beckwith J Storz G Regulation of the OxyR transcription factor by hydrogen peroxide and the cellular thiol-disulfide status Proc Natl Acad Sci USA 1999 96 6161 6165 10.1073/pnas.96.11.6161 10339558 PMC26852

[B109] Trebitsh T Levitan A Sofer A Danon A Translation of chloroplast psbA mRNA is modulated in the light by counteracting oxidizing and reducing activities Mol Cell Biol 2000 20 1116 1123 10.1128/MCB.20.4.1116-1123.2000 10648596 PMC85229

[B110] Mikkelsen RB Wardman P Biological chemistry of reactive oxygen and nitrogen and radiation-induced signal transduction mechanisms Oncogene 2003 22 5734 5754 10.1038/sj.onc.1206663 12947383

[B111] Hawkins BJ Madesh M Kirkpatrick CJ Fisher AB Superoxide flux in endothelial cells via the chloride channel-3 mediates intracellular signaling Mol Biol Cell 2007 18 2002 2012 10.1091/mbc.E06-09-0830 17360969 PMC1877121

[B112] Krinsky NI Deneke SM Interaction of oxygen and oxy-radicals with carotenoids J Natl Cancer Inst 1982 69 205 210 6285060

[B113] Stubbe J Nocera DG Yee CS Chang MC Radical initiation in the class I ribonucleotide reductase: long-range proton-coupled electron transfer? Chem Rev 2003 103 2167 2201 10.1021/cr020421u 12797828

[B114] Reece SY Seyedsayamdost MR Stubbe J Nocera DG Direct observation of a transient tyrosine radical competent for initiating turnover in a photochemical ribonucleotide reductase J Am Chem Soc 2007 129 13828 13830 10.1021/ja074452o 17944464 PMC3274171

[B115] Seyedsayamdost MR Xie J Chan CT Schultz PG Stubbe J Site-specific insertion of 3-aminotyrosine into subunit alpha2 of E. coli ribonucleotide reductase: direct evidence for involvement of Y730 and Y731 in radical propagation J Am Chem Soc 2007 129 15060 15071 10.1021/ja076043y 17990884

[B116] Rouzer CA Marnett LJ Mechanism of free radical oxygenation of polyunsaturated fatty acids by cyclooxygenases Chem Rev 2003 103 2239 2304 10.1021/cr000068x 12797830

[B117] Whittaker JW Free radical catalysis by galactose oxidase Chem Rev 2003 103 2347 2363 10.1021/cr020425z 12797833

[B118] Aubert C Vos MH Mathis P Eker AP Brettel K Intraprotein radical transfer during photoactivation of DNA photolyase Nature 2000 405 586 590 10.1038/35014644 10850720

[B119] Marquet A Bui BT Smith AG Warren MJ Iron-sulfur proteins as initiators of radical chemistry Nat Prod Rep 2007 24 1027 1040 10.1039/b703109m 17898896

[B120] Jensen RA Enzyme recruitment in evolution of new function Annu Rev Microbiol 1976 30 409 425 10.1146/annurev.mi.30.100176.002205 791073

[B121] O'Brien PJ Herschlag D Catalytic promiscuity and the evolution of new enzymatic activities Chem Biol 1999 6 R91 R105 10099128 10.1016/S1074-5521(99)80033-7

[B122] Copley SD Enzymes with extra talents: moonlighting functions and catalytic promiscuity Curr Opin Chem Biol 2003 7 265 272 10.1016/S1367-5931(03)00032-2 12714060

[B123] Kurakin A Self-organization versus Watchmaker: ambiguity of molecular recognition and design charts of cellular circuitry J Mol Recognit 2007 20 205 214 10.1002/jmr.839 17847050

[B124] Nicholson JK Wilson ID Opinion: understanding 'global' systems biology: metabonomics and the continuum of metabolism Nat Rev Drug Discov 2003 2 668 676 10.1038/nrd1157 12904817

[B125] Wilson ID Nicholson JK Topics in Xenobiochemistry: do metabolic pathways exist for xenobiotics? The micro-metabolism hypothesis Xenobiotica 2003 33 887 901 10.1080/00498250310001598221 14514439

[B126] Jeffery CJ Moonlighting proteins: old proteins learning new tricks Trends Genet 2003 19 415 417 10.1016/S0168-9525(03)00167-7 12902157

[B127] Sriram G Martinez JA McCabe ER Liao JC Dipple KM Single-gene disorders: what role could moonlighting enzymes play? Am J Hum Genet 2005 76 911 924 10.1086/430799 15877277 PMC1196451

[B128] Lovley DR Coates JD Novel forms of anaerobic respiration of environmental relevance Curr Opin Microbiol 2000 3 252 256 10.1016/S1369-5274(00)00085-0 10851154

[B129] Vargas M Kashefi K Blunt-Harris EL Lovley DR Microbiological evidence for Fe(III) reduction on early Earth Nature 1998 395 65 67 10.1038/25720 9738498

[B130] Lovley DR Coates JD Blunt-Harris EL Phillips EJP Woodward JC Humic substances as electron acceptors for microbial respiration Nature 1996 382 445 448 10.1038/382445a0

[B131] Newman DK Kolter R A role for excreted quinones in extracellular electron transfer Nature 2000 405 94 97 10.1038/35011098 10811225

[B132] Leang C Coppi MV Lovley DR OmcB, a c-type polyheme cytochrome, involved in Fe(III) reduction in Geobacter sulfurreducens J Bacteriol 2003 185 2096 2103 10.1128/JB.185.7.2096-2103.2003 12644478 PMC151516

[B133] Richardson DJ Bacterial respiration: a flexible process for a changing environment Microbiology 2000 146 Pt 3 551 571 10746759 10.1099/00221287-146-3-551

[B134] Reguera G McCarthy KD Mehta T Nicoll JS Tuominen MT Lovley DR Extracellular electron transfer via microbial nanowires Nature 2005 435 1098 1101 10.1038/nature03661 15973408

[B135] Gorby YA Yanina S McLean JS Rosso KM Moyles D Dohnalkova A Beveridge TJ Chang IS Kim BH Kim KS Electrically conductive bacterial nanowires produced by Shewanella oneidensis strain MR-1 and other microorganisms Proc Natl Acad Sci USA 2006 103 11358 11363 10.1073/pnas.0604517103 16849424 PMC1544091

[B136] Stams AJ de Bok FA Plugge CM van Eekert MH Dolfing J Schraa G Exocellular electron transfer in anaerobic microbial communities Environ Microbiol 2006 8 371 382 10.1111/j.1462-2920.2006.00989.x 16478444

[B137] Lovley DR Holmes DE Nevin KP Dissimilatory Fe(III) and Mn(IV) reduction Adv Microb Physiol 2004 49 219 286 10.1016/S0065-2911(04)49005-5 15518832

[B138] Kashefi K Lovley DR Reduction of Fe(III), Mn(IV), and toxic metals at 100 degrees C by Pyrobaculum islandicum Appl Environ Microbiol 2000 66 1050 1056 10.1128/AEM.66.3.1050-1056.2000 10698770 PMC91941

[B139] Kaden J A SG Schink B Cysteine-mediated electron transfer in syntrophic acetate oxidation by cocultures of Geobacter sulfurreducens and Wolinella succinogenes Arch Microbiol 2002 178 53 58 10.1007/s00203-002-0425-3 12070769

[B140] Chapelle FH O'Neill K Bradley PM Methe BA Ciufo SA Knobel LL Lovley DR A hydrogen-based subsurface microbial community dominated by methanogens Nature 2002 415 312 315 10.1038/415312a 11797006

[B141] Nealson KH Geomicrobiology: Sediment reactions defy dogma Nature 2010 463 1033 1034 10.1038/4631033a 20182504

[B142] Nielsen LP Risgaard-Petersen N Fossing H Christensen PB Sayama M Electric currents couple spatially separated biogeochemical processes in marine sediment Nature 2010 463 1071 1074 10.1038/nature08790 20182510

[B143] Stams AJ Plugge CM Electron transfer in syntrophic communities of anaerobic bacteria and archaea Nat Rev Microbiol 2009 7 568 577 10.1038/nrmicro2166 19609258

[B144] An S Kumar R Sheets ED Benkovic SJ Reversible compartmentalization of de novo purine biosynthetic complexes in living cells Science 2008 320 103 106 10.1126/science.1152241 18388293

[B145] Bobik TA Polyhedral organelles compartmenting bacterial metabolic processes Appl Microbiol Biotechnol 2006 70 517 525 10.1007/s00253-005-0295-0 16525780

[B146] Magistretti PJ Neuron-glia metabolic coupling and plasticity J Exp Biol 2006 209 2304 2311 10.1242/jeb.02208 16731806

[B147] Magistretti PJ Role of glutamate in neuron-glia metabolic coupling Am J Clin Nutr 2009 90 875S 880S 10.3945/ajcn.2009.27462CC 19571222

[B148] Serres S Raffard G Franconi JM Merle M Close coupling between astrocytic and neuronal metabolisms to fulfill anaplerotic and energy needs in the rat brain J Cereb Blood Flow Metab 2008 28 712 724 10.1038/sj.jcbfm.9600568 17940539

[B149] Champe PC Harvey RA Ferrier DR Lippincott's Illustrated Reviews: Biochemistry 2008 4 Baltimore, MD: Lippincott Williams & Wilkins

[B150] Sridharan V Guichard J Li CY Muise-Helmericks R Beeson CC Wright GL O(2)-sensing signal cascade: clamping of O(2) respiration, reduced ATP utilization, and inducible fumarate respiration Am J Physiol Cell Physiol 2008 295 C29 37 10.1152/ajpcell.00466.2007 18463229 PMC2493545

[B151] Weinberg JM Venkatachalam MA Roeser NF Nissim I Mitochondrial dysfunction during hypoxia/reoxygenation and its correction by anaerobic metabolism of citric acid cycle intermediates Proc Natl Acad Sci USA 2000 97 2826 2831 10.1073/pnas.97.6.2826 10717001 PMC16014

[B152] Hochachka PW Storey KB Metabolic consequences of diving in animals and man Science 1975 187 613 621 10.1126/science.163485 163485

[B153] Segerer A Stetter KO Klink F Two contrary modes of chemolithotrophy in the same archaebacterium Nature 1985 313 787 789 10.1038/313787a0 3919307

[B154] Simon MC Keith B The role of oxygen availability in embryonic development and stem cell function Nat Rev Mol Cell Biol 2008 9 285 296 10.1038/nrm2354 18285802 PMC2876333

[B155] Webster KA Evolution of the coordinate regulation of glycolytic enzyme genes by hypoxia J Exp Biol 2003 206 2911 2922 10.1242/jeb.00516 12878660

[B156] Sun PD Davies DR The cystine-knot growth-factor superfamily Annu Rev Biophys Biomol Struct 1995 24 269 291 10.1146/annurev.bb.24.060195.001413 7663117

[B157] Graham JW Williams TC Morgan M Fernie AR Ratcliffe RG Sweetlove LJ Glycolytic enzymes associate dynamically with mitochondria in response to respiratory demand and support substrate channeling Plant Cell 2007 19 3723 3738 10.1105/tpc.107.053371 17981998 PMC2174870

[B158] Winter SE Thiennimitr P Winter MG Butler BP Huseby DL Crawford RW Russell JM Bevins CL Adams LG Tsolis RM Gut inflammation provides a respiratory electron acceptor for Salmonella Nature 2010 467 426 429 10.1038/nature09415 20864996 PMC2946174

[B159] DeYulia GJ Jr Carcamo JM Borquez-Ojeda O Shelton CC Golde DW Hydrogen peroxide generated extracellularly by receptor-ligand interaction facilitates cell signaling Proc Natl Acad Sci USA 2005 102 5044 5049 10.1073/pnas.0501154102 15795385 PMC556007

[B160] Wentworth AD Jones LH Wentworth P Jr Janda KD Lerner RA Antibodies have the intrinsic capacity to destroy antigens Proc Natl Acad Sci USA 2000 97 10930 10935 10.1073/pnas.97.20.10930 11005865 PMC27126

[B161] Wentworth P Jr Jones LH Wentworth AD Zhu X Larsen NA Wilson IA Xu X Goddard WA Janda KD Eschenmoser A Lerner RA Antibody catalysis of the oxidation of water Science 2001 293 1806 1811 10.1126/science.1062722 11546867

[B162] Reth M Hydrogen peroxide as second messenger in lymphocyte activation Nat Immunol 2002 3 1129 1134 10.1038/ni1202-1129 12447370

[B163] Klyubin IV Kirpichnikova KM Gamaley IA Hydrogen peroxide-induced chemotaxis of mouse peritoneal neutrophils Eur J Cell Biol 1996 70 347 351 8864663

[B164] Niethammer P Grabher C Look AT Mitchison TJ A tissue-scale gradient of hydrogen peroxide mediates rapid wound detection in zebrafish Nature 2009 459 996 999 10.1038/nature08119 19494811 PMC2803098

[B165] Yoo SK Huttenlocher A Innate immunity: wounds burst H2O2 signals to leukocytes Curr Biol 2009 19 R553 555 10.1016/j.cub.2009.06.025 19640490

[B166] Bae YS Choi MK Lee WJ Dual oxidase in mucosal immunity and host-microbe homeostasis Trends Immunol 2010 31 278 287 10.1016/j.it.2010.05.003 20579935

[B167] Ushio-Fukai M Alexander RW Reactive oxygen species as mediators of angiogenesis signaling: role of NAD(P)H oxidase Mol Cell Biochem 2004 264 85 97 10.1023/B:MCBI.0000044378.09409.b5 15544038

[B168] Bedard K Krause KH The NOX family of ROS-generating NADPH oxidases: physiology and pathophysiology Physiol Rev 2007 87 245 313 10.1152/physrev.00044.2005 17237347

[B169] Ciobanu M Taylor DE Jr Wilburn JP Cliffel DE Glucose and lactate biosensors for scanning electrochemical microscopy imaging of single live cells Anal Chem 2008 80 2717 2727 10.1021/ac7021184 18345647 PMC2836715

[B170] Hochachka PW The metabolic implications of intracellular circulation Proc Natl Acad Sci USA 1999 96 12233 12239 10.1073/pnas.96.22.12233 10535904 PMC34257

[B171] Wikipedia Stigmergy 2010 http://en.wikipedia.org/wiki/Stigmergy

[B172] Wikipedia Qi 2010 http://en.wikipedia.org/wiki/Qi

[B173] Wikipedia Hormesis 2010 http://en.wikipedia.org/wiki/Hormesis

[B174] Agutter PS Cell mechanics and stress: from molecular details to the 'universal cell reaction' and hormesis Bioessays 2007 29 324 333 10.1002/bies.20550 17373655

[B175] Matveev VV Protoreaction of protoplasm Cell Mol Biol (Noisy-le-grand) 2005 51 715 723 16359621

[B176] Voeikov V Beloussov L, Voeikov V, Martynyuk V Fundamental role of water in bioenergetics Biophotonics and Coherent Systems in Biology 2007 New York: Springer 89 104

[B177] Gregory TR Macroevolution, hierarchy theory, and the C-value enigma Paleobiology 2004 30 179 202 10.1666/0094-8373(2004)030<0179:MHTATC>2.0.CO;2

[B178] Imlay JA Cellular defenses against superoxide and hydrogen peroxide Annu Rev Biochem 2008 77 755 776 10.1146/annurev.biochem.77.061606.161055 18173371 PMC3057177

[B179] Venugopalan M Jones RA Chemistry of Dissociated Water Vapor and Related Systems 1968 New York: Wiley

[B180] Kasting JF Earth's early atmosphere Science 1993 259 920 926 10.1126/science.11536547 11536547

[B181] Nisbet EG Sleep NH The habitat and nature of early life Nature 2001 409 1083 1091 10.1038/35059210 11234022

[B182] Yung Y Wen JS Moses JI Landry BM Allen M Hydrogen and deuterium loss from the terrestrial atmosphere: a quantitative assessment of non-thermal escape fluxes J Geophys Res 1989 94 14971 14989 10.1029/JD094iD12p14971 11538865

[B183] Nieva J Kerwin L Wentworth AD Lerner RA Wentworth P Jr Immunoglobulins can utilize riboflavin (Vitamin B2) to activate the antibody-catalyzed water oxidation pathway Immunol Lett 2006 103 33 38 10.1016/j.imlet.2005.11.020 16386801

[B184] Nieva J Wentworth P Jr The antibody-catalyzed water oxidation pathway--a new chemical arm to immune defense? Trends Biochem Sci 2004 29 274 278 10.1016/j.tibs.2004.03.009 15130564

[B185] Xu X Muller RP Goddard WA The gas phase reaction of singlet dioxygen with water: a water-catalyzed mechanism Proc Natl Acad Sci USA 2002 99 3376 3381 10.1073/pnas.052710099 11891316 PMC122531

[B186] Plesnicar B Progress in the Chemistry of Dihydrogen Trioxide (HOOOH) Acta Chim Slov 2005 52 1 12

[B187] Yamashita K Miyoshi T Arai T Endo N Itoh H Makino K Mizugishi K Uchiyama T Sasada M Ozone production by amino acids contributes to killing of bacteria Proc Natl Acad Sci USA 2008 105 16912 16917 10.1073/pnas.0807952105 18971328 PMC2579352

[B188] Woutersen S Bakker HJ Resonant intermolecular transfer of vibrational energy in liquid water Nature 1999 402 507 509 10.1038/990058

[B189] Chaplin M Do we underestimate the importance of water in cell biology? Nat Rev Mol Cell Biol 2006 7 861 866 10.1038/nrm2021 16955076

[B190] Friedman R Fischer S Nachliel E Scheiner S Gutman M Minimum energy pathways for proton transfer between adjacent sites exposed to water J Phys Chem B 2007 111 6059 6070 10.1021/jp070781r 17488114

[B191] Laage D Hynes JT A molecular jump mechanism of water reorientation Science 2006 311 832 835 10.1126/science.1122154 16439623

[B192] Laage D Hynes JT On the molecular mechanism of water reorientation J Phys Chem B 2008 112 14230 14242 10.1021/jp805217u 18942871

[B193] Fenn EE Wong DB Fayer MD Water dynamics at neutral and ionic interfaces Proc Natl Acad Sci USA 2009 106 15243 15248 10.1073/pnas.0907875106 19706895 PMC2741235

[B194] Higgins MJ Polcik M Fukuma T Sader JE Nakayama Y Jarvis SP Structured water layers adjacent to biological membranes Biophys J 2006 91 2532 2542 10.1529/biophysj.106.085688 16798815 PMC1562391

[B195] Luby-Phelps K Cytoarchitecture and physical properties of cytoplasm: volume, viscosity, diffusion, intracellular surface area Int Rev Cytol 2000 192 189 221 full_text 10553280 10.1016/s0074-7696(08)60527-6

[B196] Damadian R Tumor detection by nuclear magnetic resonance Science 1971 171 1151 1153 10.1126/science.171.3976.1151 5544870

[B197] Halpern HJ Chandramouli GV Barth ED Yu C Peric M Grdina DJ Teicher BA Diminished aqueous microviscosity of tumors in murine models measured with in vivo radiofrequency electron paramagnetic resonance Cancer Res 1999 59 5836 5841 10582707

[B198] Nisini B ASTRONOMY: Enhanced: Water's Role in Making Stars Science 2000 290 1513 1514 10.1126/science.290.5496.1513 17771224

[B199] Bethell T Bergin E Formation and survival of water vapor in the terrestrial planet-forming region Science 2009 326 1675 1677 10.1126/science.1176879 20019283

[B200] Vohringer-Martinez E Hansmann B Hernandez-Soto H Francisco JS Troe J Abel B Water catalysis of a radical-molecule gas-phase reaction Science 2007 315 497 501 10.1126/science.1134494 17255507

[B201] Mordaunt DH Ashford MNR Dixon RN Dissociation dynamics of H2O(D2O) following photoexcitation at the Lyman-alpha wavelength (121.6 nm) J Chem Phys 1994 100 7360 10.1063/1.466880

[B202] Tappe A Lada CJ Black JH Muench AA Discovery of Superthermal Hydroxyl (OH) in the HH 211 Outflow Astrophys J 2008 680 117 120 10.1086/589998

[B203] Wooden DH Charnley SB Ehrenfreund P Composition and Evolution of Interstellar Clouds 2004 Tucson AZ: University of Arizona Press

[B204] Carr JS Najita JR Organic molecules and water in the planet formation region of young circumstellar disks Science 2008 319 1504 1506 10.1126/science.1153807 18339932

[B205] Dworkin J Deamer D Sandford S Allamandola L Self-assembling amphiphilic molecules: Synthesis in simulated interstellar/precometary ices Proc Natl Acad Sci USA 2001 98 815 819 10.1073/pnas.98.3.815 11158552 PMC14665

[B206] Sandford SA Aleon J Alexander CM Araki T Bajt S Baratta GA Borg J Bradley JP Brownlee DE Brucato JR Organics captured from comet 81P/Wild 2 by the Stardust spacecraft Science 2006 314 1720 1724 10.1126/science.1135841 17170291

[B207] Cronin JR Pizzarello S Cruikshank DP Organic matter in carbonaceous chondrites, planetary satellites, asteroids and comets 1988 Tucson AZ: University of Arizona Press

[B208] Cronin JR Chang S Organic matter in meteorites: molecular and isotopic analyses of the Murchison meteorite 1993 Dordrecht, Netherlands: Kluwer Academic Publishers

[B209] Sephton MA Organic matter in carbonaceous meteorites: past, present and future research Philos Transact A Math Phys Eng Sci 2005 363 2729 2742 10.1098/rsta.2005.1670 16286287

[B210] Ehrenfreund P Sephton MA Carbon molecules in space: from astrochemistry to astrobiology Faraday Discuss 2006 133 277 288 discussion 347-274, 449-252 10.1039/b517676j 17191452

[B211] Wikipedia Murchison meteorite 2010 http://en.wikipedia.org/wiki/Murchison_meteorite

[B212] Studier MH Hayatsu R Anders E Origin of organic matter in early solar system; I. Hydrocarbons Geochim Cosmochim Acta 1968 32 151 173 10.1016/S0016-7037(68)80002-X

[B213] Hodgson GW Baker BL Porphyrin abiogenesis from pyrrole and formaldehyde under simulated geochemical conditions Nature 1967 216 29 32 10.1038/216029a0 6050667

[B214] Kenney JF Kutcherov VA Bendeliani NA Alekseev VA The evolution of multicomponent systems at high pressures: VI. The thermodynamic stability of the hydrogen-carbon system: the genesis of hydrocarbons and the origin of petroleum Proc Natl Acad Sci USA 2002 99 10976 10981 10.1073/pnas.172376899 12177438 PMC123195

[B215] Wikipedia Fischer-Tropsch process 2010 http://en.wikipedia.org/wiki/Fischer%E2%80%93Tropsch_process

[B216] Anderson RB The Fischer-Tropsch Synthesis 1984 Orlando, FL: Academic Press

[B217] Anders E Hayatsu R Studier MH Organic Compounds in Meteorites: They may have formed in the solar nebula, by catalytic reactions of carbon monoxide, hydrogen, and ammonia Science 1973 182 781 790 10.1126/science.182.4114.781 17772148

[B218] Studier MH Hayatsu R Anders E Origin of organic matter in early solar system-V. Further studies of meteoritic hydrocarbons and a discussion of their origin Geochim Cosmochim Acta 1972 36 189 215 10.1016/0016-7037(72)90006-3

[B219] Kenney JF Shnyukov YF Krayushkin VA Karpov IK Kutcherov VG Plotnikova IN Dismissal of Claims of a Biological Connection for Natural Petroleum Energia 2001 22 26 34

[B220] Wikipedia Abiogenic petroleum origin 2010 http://en.wikipedia.org/wiki/Abiogenic_petroleum_origin

[B221] Hill HG Nuth JA The catalytic potential of cosmic dust: implications for prebiotic chemistry in the solar nebula and other protoplanetary systems Astrobiology 2003 3 291 304 10.1089/153110703769016389 14577878

[B222] Takahashi J Masuda H Kaneko T Kobayashi K Saito T Hosokawa T Photochemical abiotic synthesis of amino-acid precursors from simulated planetary atmospheres by vacuum ultraviolet light J Appl Phys 2005 98 024907 10.1063/1.1968438

[B223] Proskurowski G Lilley MD Seewald JS Fruh-Green GL Olson EJ Lupton JE Sylva SP Kelley DS Abiogenic hydrocarbon production at lost city hydrothermal field Science 2008 319 604 607 10.1126/science.1151194 18239121

[B224] Miller SL A production of amino acids under possible primitive earth conditions Science 1953 117 528 529 10.1126/science.117.3046.528 13056598

[B225] Johnson AP Cleaves HJ Dworkin JP Glavin DP Lazcano A Bada JL The Miller volcanic spark discharge experiment Science 2008 322 404 10.1126/science.1161527 18927386

[B226] Kasting JF Eggler DH Raeburn SP Mantle redox evolution and the oxidation state of the Archean atmosphere J Geol 1993 101 245 257 10.1086/648219 11537741

[B227] Gold T The deep, hot biosphere Proc Natl Acad Sci USA 1992 89 6045 6049 10.1073/pnas.89.13.6045 1631089 PMC49434

[B228] Falkowski PG Barber RT Smetacek VV Biogeochemical Controls and Feedbacks on Ocean Primary Production Science 1998 281 200 207 10.1126/science.281.5374.200 9660741

[B229] Field CB Behrenfeld MJ Randerson JT Falkowski P Primary production of the biosphere: integrating terrestrial and oceanic components Science 1998 281 237 240 10.1126/science.281.5374.237 9657713

[B230] Zehr JP Ward BB Nitrogen cycling in the ocean: new perspectives on processes and paradigms Appl Environ Microbiol 2002 68 1015 1024 10.1128/AEM.68.3.1015-1024.2002 11872445 PMC123768

[B231] Villareal TA Pilskaln C Brzezinski M Lipschultz F Dennett M Gardner GB Upward transport of oceanic nitrate by migrating diatom mats Nature 1999 397 423 425 10.1038/17103 29667969

[B232] Redfield AC The biological control of chemical factors in the environment Am Sci 1958 46 205 221 24545739

[B233] Falkowski PG Evolution of the nitrogen cycle and its influence on the biological sequestration of CO2 in the ocean Nature 1997 387 272 275 10.1038/387272a0

[B234] Holland HD The Chemical Evolution of the Atmosphere and Oceans 1984 Princeton, NJ: Princeton Univ. Press

[B235] Kolber ZS Barber RT Coale KH Fitzwateri SE Greene RM Johnson KS Lindley S Falkowski PG Iron limitation of phytoplankton photosynthesis in the equatorial Pacific Ocean Nature 1994 371 145 149 10.1038/371145a0

[B236] Azam F Microbial Control of Oceanic Carbon Flux: The Plot Thickens Science 1998 280 694 696 10.1126/science.280.5364.694

[B237] Martin W Baross J Kelley D Russell MJ Hydrothermal vents and the origin of life Nat Rev Microbiol 2008 6 805 814 18820700 10.1038/nrmicro1991

[B238] Rasmussen B Filamentous microfossils in a 3,235-million-year-old volcanogenic massive sulphide deposit Nature 2000 405 676 679 10.1038/35015063 10864322

[B239] Kelley DS Karson JA Fruh-Green GL Yoerger DR Shank TM Butterfield DA Hayes JM Schrenk MO Olson EJ Proskurowski G A serpentinite-hosted ecosystem: the Lost City hydrothermal field Science 2005 307 1428 1434 10.1126/science.1102556 15746419

[B240] Beatty JT Overmann J Lince MT Manske AK Lang AS Blankenship RE Van Dover CL Martinson TA Plumley FG An obligately photosynthetic bacterial anaerobe from a deep-sea hydrothermal vent Proc Natl Acad Sci USA 2005 102 9306 9310 10.1073/pnas.0503674102 15967984 PMC1166624

[B241] Nisbet EG Cann JR Van Dover CL Origins of photosynthesis Nature 1995 373 479 480 10.1038/373479a0

[B242] Larkum AW Kuhl M Chlorophyll d: the puzzle resolved Trends Plant Sci 2005 10 355 357 10.1016/j.tplants.2005.06.005 16019251

[B243] Chen M Schliep M Willows RD Cai ZL Neilan BA Scheer H A red-shifted chlorophyll Science 2010 329 1318 1319 10.1126/science.1191127 20724585

[B244] Ettwig KF Butler MK Le Paslier D Pelletier E Mangenot S Kuypers MM Schreiber F Dutilh BE Zedelius J de Beer D Nitrite-driven anaerobic methane oxidation by oxygenic bacteria Nature 2010 464 543 548 10.1038/nature08883 20336137

[B245] Ducluzeau AL van Lis R Duval S Schoepp-Cothenet B Russell MJ Nitschke W Was nitric oxide the first deep electron sink? Trends Biochem Sci 2009 34 9 15 10.1016/j.tibs.2008.10.005 19008107

[B246] Roussel EG Bonavita MA Querellou J Cragg BA Webster G Prieur D Parkes RJ Extending the sub-sea-floor biosphere Science 2008 320 1046 10.1126/science.1154545 18497290

[B247] Pikuta EV Hoover RB Tang J Microbial extremophiles at the limits of life Crit Rev Microbiol 2007 33 183 209 10.1080/10408410701451948 17653987

[B248] Makarova KS Aravind L Wolf YI Tatusov RL Minton KW Koonin EV Daly MJ Genome of the extremely radiation-resistant bacterium Deinococcus radiodurans viewed from the perspective of comparative genomics Microbiol Mol Biol Rev 2001 65 44 79 10.1128/MMBR.65.1.44-79.2001 11238985 PMC99018

[B249] Azam F Worden AZ Oceanography. Microbes, molecules, and marine ecosystems Science 2004 303 1622 1624 10.1126/science.1093892 15016987

[B250] Alain K Querellou J Cultivating the uncultured: limits, advances and future challenges Extremophiles 2009 13 583 594 10.1007/s00792-009-0261-3 19548063

[B251] Hemley RJ Effects of high pressure on molecules Annu Rev Phys Chem 2000 51 763 800 10.1146/annurev.physchem.51.1.763 11031299

[B252] Schettino V Bini R Ceppatelli M Ciabini L Citroni M Chemical reactions at very high pressure Adv Chem Phys 2005 131 105 242 full_text

[B253] Drickamer HG Frank CW Electronic structure, electronic transitions, and the high pressure chemistry and physics of solids Annu Rev Phys Chem 1972 23 39 64 10.1146/annurev.pc.23.100172.000351

[B254] Aoki K Usuba S Yoshida M Kakudate Y Tanaka K Fujiwara S Raman study of the solid-state polymerization of acetylene at high pressure J Phys Chem 1988 89 529 534 10.1063/1.455441

[B255] Ceppatelli M Bini R Schettino V High-pressure photodissociation of water as a tool for hydrogen synthesis and fundamental chemistry Proc Natl Acad Sci USA 2009 106 11454 11459 10.1073/pnas.0901836106 19581572 PMC2710632

[B256] Ceppatelli M Bini R Schettino V High-pressure reactivity of model hydrocarbons driven by near-UV photodissociation of water J Phys Chem B 2009 113 14640 14647 10.1021/jp9070482 19824614

[B257] Chelazzi D Ceppatelli M Santoro M Bini R Schettino V High-pressure synthesis of crystalline polyethylene using optical catalysis Nat Mater 2004 3 470 475 10.1038/nmat1147 15184892

[B258] Daniel I Oger P Winter R Origins of life and biochemistry under high-pressure conditions Chem Soc Rev 2006 35 858 875 10.1039/b517766a 17003893

[B259] Wikipedia Double layer (plasma) 2010 http://en.wikipedia.org/wiki/Double_layer_%28plasma%29

[B260] Wikipedia Plasma (physics) 2010 http://en.wikipedia.org/wiki/Plasma_%28physics%29

[B261] Alfvén H Cosmic plasma 1981 Dordrecht, Holland: D. Reidel Publishing Company

[B262] Ling GN Debunking the alleged resurrection of the sodium pump hypothesis Physiol Chem Phys Med NMR 1997 29 123 198 9654772

[B263] Ling GN Life at the Cell and Below-Cell Level 2001 New York: Pacific Press

[B264] Pollack GH Cells, Gels and the Engines of Life 2001 Seattle: Ebner & Sons

[B265] Wikipedia Protoplasm 2010 http://en.wikipedia.org/wiki/Protoplasm

[B266] Wikipedia Plasma scaling 2010 http://en.wikipedia.org/wiki/Plasma_scaling

[B267] Evers F Mirlin AD Anderson transitions Reviews of Modern Physics 2008 80 1355 1417 10.1103/RevModPhys.80.1355

[B268] Richardella A Roushan P Mack S Zhou B Huse DA Awschalom DD Yazdani A Visualizing critical correlations near the metal-insulator transition in Ga(1-x)Mn(x)As Science 2010 327 665 669 10.1126/science.1183640 20133566

[B269] Bak P How nature works: the science of self-organized criticality 1996 New York: Copernicus, Springer-Verlag New York, Inc

[B270] Bollinger JM Jr Biochemistry. Electron relay in proteins Science 2008 320 1730 1731 10.1126/science.1160001 18583602

[B271] Larsen RE Glover WJ Schwartz BJ Does the hydrated electron occupy a cavity? Science 2010 329 65 69 10.1126/science.1189588 20595609

[B272] Wikipedia File:Plasma-lamp 2.jpg 2010 http://en.wikipedia.org/wiki/File:Plasma-lamp_2.jpg

